# Structural and biophysical insights into the mode of covalent binding of rationally designed potent BMX inhibitors†

**DOI:** 10.1039/d0cb00033g

**Published:** 2020-08-28

**Authors:** João D. Seixas, Bárbara B. Sousa, Marta C. Marques, Ana Guerreiro, Rui Traquete, Tiago Rodrigues, Inês S. Albuquerque, Marcos F. Q. Sousa, Ana R. Lemos, Pedro M. F. Sousa, Tiago M. Bandeiras, Di Wu, Shelby K. Doyle, Carol V. Robinson, Angela N. Koehler, Francisco Corzana, Pedro M. Matias, Gonçalo J. L. Bernardes

**Affiliations:** Instituto de Medicina Molecular João Lobo Antunes, Faculdade de Medicina, Universidade de Lisboa Avenida Professor Egas Moniz 1649-028 Lisboa Portugal gbernardes@medicina.ulisboa.pt joaoseixas@medicina.ulisboa.pt; Instituto de Tecnologia Química e Biológica António Xavier, Universidade Nova de Lisboa Av. da República EAN 2780-157 Oeiras Portugal; IBET - Instituto de Biologia Experimental e Tecnológica Av. da República EAN 2780-157 Oeiras Portugal; Department of Chemistry, University of Oxford South Parks Road Oxford OX1 3QZ UK; David H. Koch Institute for Integrative Cancer Research, Massachusetts Institute of Technology Cambridge MA 02142 USA; Departamento de Química, Universidad de La Rioja, Centro de Investigación en Síntesis Química 26006 Logroño Spain; Department of Chemistry, University of Cambridge Lensfield Road Cambridge CB2 1EW UK gb453@cam.ac.uk

## Abstract

The bone marrow tyrosine kinase in chromosome X (BMX) is pursued as a drug target because of its role in various pathophysiological processes. We designed BMX covalent inhibitors with single-digit nanomolar potency with unexploited topological pharmacophore patterns. Importantly, we reveal the first X-ray crystal structure of covalently inhibited BMX at Cys496, which displays key interactions with Lys445, responsible for hampering ATP catalysis and the DFG-out-like motif, typical of an inactive conformation. Molecular dynamic simulations also showed this interaction for two ligand/BMX complexes. Kinome selectivity profiling showed that the most potent compound is the strongest binder, displays intracellular target engagement in BMX-transfected cells with two-digit nanomolar inhibitory potency, and leads to BMX degradation PC3 in cells. The new inhibitors displayed anti-proliferative effects in androgen-receptor positive prostate cancer cells that where further increased when combined with known inhibitors of related signaling pathways, such as PI3K, AKT and Androgen Receptor. We expect these findings to guide development of new selective BMX therapeutic approaches.

## Abstract 2

We identified potent, functionalisable BMX inhibitors and revealed their covalent mode of binding to BMX by X-ray crystallography.

## Graphical abstract

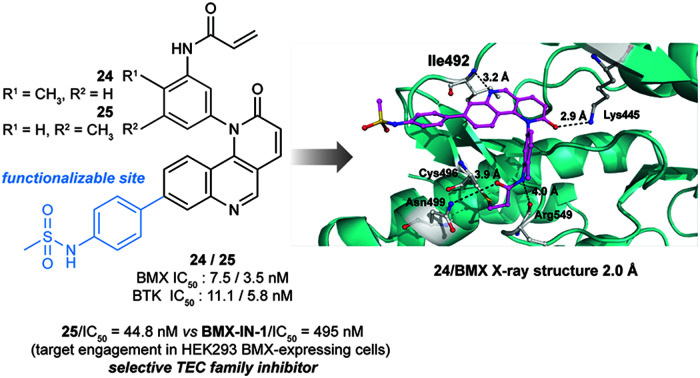

## Introduction

Over recent years, the development of covalent kinase inhibitors has gained more traction both in academia and pharmaceutical industry.^1–3^ Historically, irreversible covalent inhibitors were considered unsafe because of their lack of selectivity and concomitant undesired engagement of off-targets. However, these potential liabilities can be overcome and the development of covalent small molecule kinase inhibitors has recently seen renewed interest. Irreversible covalent inhibitors can display higher efficacy, since they achieve high target occupancy and a prolonged pharmacodynamic effect, depending on the *de novo* re-synthesis rate of the target protein.^4,5^ Supporting the value and “renaissance” of covalent inhibitors, since October 2018 six kinase-targeting small molecule covalent inhibitors^6^ were approved by the FDA for clinical use: the EGFR inhibitors Afatinib®, Neratinib®, Osimertinib® and Dacomitinib® and the BTK inhibitors Ibrutinib® and Acalabrutinib®.^7–11^ However, not all kinases are accessible for covalent binding since the covalent bond formation depends on the nature and positioning of the target amino acid.^12–15^ One such kinase of interest is the epithelial and endothelial tyrosine kinase, commonly known as bone marrow tyrosine kinase in chromosome X (BMX). BMX is a major member of the TEC family of non-receptor tyrosine kinases, together with ITK, TEC, BTK and TXK [reviewed in ref. 16 and 17]. TEC kinases are activated by many cell-surface receptor-associated signaling complexes and are recruited to the plasma membrane or specific micro-environments by a variety of lipids and proteins. Through this mechanism, they are involved in signal transduction in response to a myriad of extracellular stimuli, including those mediated by growth-factor receptors, cytokine receptors, G-protein coupled receptors, antigen-receptors, integrins and death receptors. Moreover, TEC kinases regulate many of the major signaling pathways, such as those of PI3K, PKC, PLCγ, AKT, STAT3 and p21-activated kinase 1 (PAK1)^18,19^ and are responsible for a variety of cell processes, including regulation of gene expression, calcium mobilization, actin reorganization/motility and survival/apoptosis.^16,17^

BMX is widely expressed in granulocytes, monocytes, cells of epithelial and endothelial lineages, as well as brain, prostate, lung and heart.^19–22^ It is specifically involved in tumorigenicity, adhesion, motility, angiogenesis, proliferation and differentiation. Moreover, it has been found to be overexpressed in numerous cancer types, such as breast,^23–25^ prostate,^26,27^ colon^28^ and cervical carcinoma,^29^ which suggests that elevated levels of BMX increase cancer-cell survival. BMX is also required for stem-cell maintenance and survival^22^ and its up-regulation provides a survival benefit to both primary tumors and cancer stem cells that are highly resistant to apoptosis and many chemotherapeutic agents.

Homozygous BMX knockout mice have a normal life span without any obvious altered phenotype, which suggests that therapies based on BMX inhibition might have few side effects^30^ and although BMX is a key regulator it might not represent a fundamental effector. Therefore, by considering the existence of multiple downstream target proteins, the integration in multiple and diverse signaling pathways, and the fact that it regulates proliferation, migration and has an anti-apoptotic effect, BMX emerges as a potential target for multiple aspects of cancer therapy. Recent studies also highlighted that modulation of BMX activity sensitizes cells to therapeutic agents to improve response to chemotherapy DNA damaging agents or radiation. These studies show strong evidence that both direct inhibition of BMX and modulation of related pathways result in increased therapeutic efficacy.^28,31,32^

BMX-IN-1 is one of the most potent BMX inhibitors (IC_50_: 8.0 nM) reported in the literature, which also binds to BTK with very high affinity (IC_50_: 10.4 nM).^33^ Like other BMX covalent inhibitors, it reacts with a cysteine residue (Cys496) in the ATP binding site. This residue is a unique occurrence found in the ATP binding pocket and is present in all five members of the TEC family kinase members. Therefore, by virtue of structural homology these compounds could also be covalent inhibitors of the other kinases in the TEC family.

In this study, we describe the discovery of **JS24–JS27**, which are among the most potent covalent inhibitors of BMX reported to date and possess topological pharmacophoric features not exploited in the BMX inhibitors’ chemical space. We asserted the selectivity against a panel of 36 kinases that possess an equally placed cysteine or up- and downstream regulators of the BMX signaling pathway. We further demonstrated that the lead compounds have the potential to inhibit proliferation of androgen-receptor-positive prostate-cancer cells (LNCaP) and their inhibitory potential is enhanced in a co-treatment regimen with known PI3K, AKT and androgen receptor inhibitors (LY294002, AKT1/2 and Flutamide, respectively). As part of our efforts to explore this scaffold to identify regions of the molecule amenable to conjugation we also report the first X-ray structure of BMX with a covalent inhibitor as well as MD simulations on two complexes with this receptor, which provide insight into the mode of binding and will contribute towards the future development of inhibitors with improved efficacy and selectivity.

## Results and discussion

### Discovery of a single-digit nanomolar BMX inhibitor

**Fig. 1:**
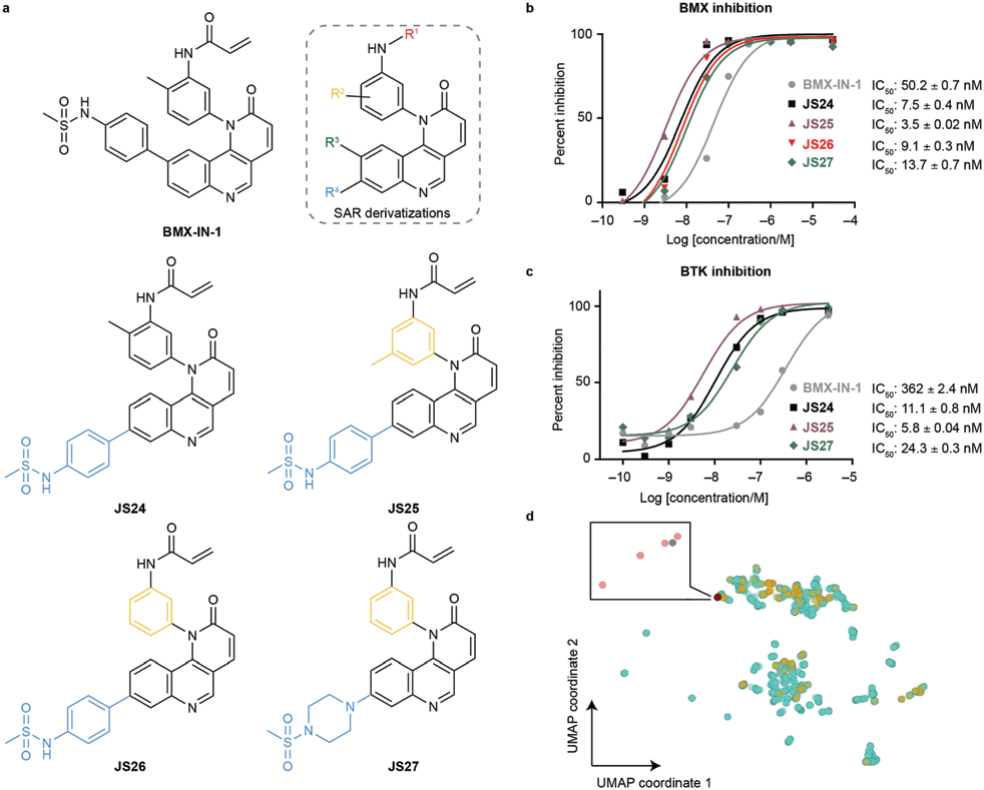
Compound structures and biochemical characterization of their inhibitory effects on BMX and BTK. (a) The structures of **BMX-IN-1**, the SAR explored (shown in Fig. S1, ESI†) and **JS24–JS27** leads generated in this study. (b) Eurofins DiscoverX *in vitro* BMX activity evaluation by measuring the phosphorylation of a biotinylated peptide with human recombinant enzyme expressed in insect cells and HTRF detection method, tested in duplicate, showing mean ± S.D. Cells were treated for 1 h and IC_50_ values were calculated and plotted by using GraphPad Prism 8 based on a sigmoidal dose response curve. (c) Eurofins DiscoverX *in vitro* BTK activity evaluation by measuring scintillation count with a radiometric assay tested in duplicate, showing mean ± S.D. Cells were treated for 1 h and IC_50_ values were calculated and plotted by using GraphPad Prism 8 based on a sigmoidal dose response curve. (d) Projection of BMX and BTK inhibitor space with a multidimensional scaling algorithm. Green: BTK inhibitors; orange: BMX inhibitors; grey: **BMX-IN-1**; red: **JS24–J27**.

To evaluate substituent tolerability at each position and to establish an optimal vector through positioning of different functionalities, a structure–activity relationship (SAR) study was used to establish the limitations of the tool chemotype **BMX-IN-1**. A total of 24 analogues were synthesized in an attempt to both enhance potency and optimize physicochemical properties within the allowable SAR study (Fig. S1, ESI†). Upon systematic evaluation, we found that analogues with substituents in position R^3^ (Fig. 1a) of the quinoline moiety had only marginal effects on BMX inhibition Furthermore, any change of the electrophilic warhead (position R^1^, Fig. 1a) for cysteine covalent ligation resulted in loss of potency as illustrated for instance by the introduction of the enamide substituents in **JS10** and **JS11** (Fig. S1, ESI†). The substituents in the aromatic ring bearing the amide functionality play an unexpected relevant role for the activity, affording different reactivity patterns arising from non-covalent interactions. Introducing a strong electron-donating group such as a methoxy group (OMe) (**JS9E**) in R^2^ decreases potency by 4-fold. In contrast, the use of a weak electron-donating group, such as a methyl substituent, has different effects depending on the positioning. Thus, moving the methyl group to the ortho position abolishes target inhibition (**JS9C**), while a methyl positioning in the meta position slightly improved inhibition by 2-fold. Even more striking is the effect of no substituent in the ring (**JS9D**), which increased inhibition by 6-fold. Since the electronic influence of the methyl substituents in the different positions is not expected to account for these differences, we consider that a conformational effect may play an important role. The ortho substitution may increase the constraints for fitting into the pocket, while the removal of the methyl groups affords less spatial restriction.

Interestingly, substituents in position R^4^ (Fig. 1a and Fig. S2, ESI†) were found to significantly enhance BMX inhibition relative to **BMX-IN-1** (Fig. 1a and Fig. S2, ESI†). With this SAR information in hand, we decided to prepare **JS24–JS27** that were designed to include substituents at position R^4^ of the quinoline and incorporated features from previous analogues that afforded a preferred overall profile, varying methyl positioning in R^2^ and retaining the acrylamide electrophilic warhead for cysteine covalent binding. Details for the synthesis of **JS24–JS27** can be found in the ESI† (Schemes S1–S4). Compound **JS24**, which features a methyl sulfonamide at position 7, showed considerable gain of inhibition potency from IC_50_ 50 to 7.5 nM relative to the parent molecule **BMX-IN-1** (Fig. 1a and b). Further derivatives that presented changes in the aniline core (R^2^) with a methyl in the *meta* position (**JS25**) and without any substituent in this position (**JS26**), but that features the same methyl sulfonamide at R^4^, were designed and prepared. Both compounds showed significantly improved activity (IC_50_ of 3.5 and 9.1 nM, respectively; Fig. 1a and 1b).

Similarly, in **JS27** we installed a substituted piperazine in the R^4^ position that affords less restraint relative to the aromatic phenyl-sulfonamide ring, but which renders it the least active analogue of the series (13.7 nM) albeit considerably more potent (≈4-fold) relative to **BMX-IN-1** (Fig. 1a and b).

### Modulation of physicochemical profile, BTK inhibition and pharmacophore diversity

Taking into account that **BMX-IN-1** does not exhibit an optimal physicochemical profile, we aimed at lowering the lipophilicity and increasing water solubility within the established SAR. Initial investigations showed an improved profile when the sulfonamide aromatic ring was replaced by cyclic aliphatic amines (Table S1, ESI†). These observations prompted us to use 1-(methylsulfonyl)piperazine in compound **JS27**. Consequently, we were able to obtain the analogue with the best *in silico* lipophilicity and water solubility profile (*c* log *P* = 2.32 and log *S* = −4.36; Table S1, ESI†). Interestingly, compound **JS26** shows a slightly improved reduction of *c* log *P*. The presence of a methyl group (compounds **JS24** and **JS25**) increases hydrophobicity and, consequently, removal of this group (**JS26–JS27**) decreases the hydrophobicity and the partition coefficient.

We anticipated that some analogues could have limited membrane permeability, which is of utmost importance for any drug, in particular if a molecule is targeting cytoplasmic proteins. For assessment of drug permeability, we relied on parallel artificial membrane permeability assay (PAMPA) performed at Pion Inc. with the PAMPA Evolution™ instrument (Table S1, ESI†). We observed that compounds **JS24** (6.8 × 10^−6^ cm s^−1^) and **JS25** (3.8 × 10^−6^ cm s^−1^) show a lower PAMPA permeability relative to **BMX-IN-1** (8.9 × 10^−6^ cm s^−1^), whereas compounds with the unsubstituted backbone aniline (**JS26** and **JS27**) show increased permeability (19 × 10^−6^ cm s^−1^ and 12 × 10^−6^ cm s^−1^, respectively). As shown in Fig. 1a, the four leads share a similar scaffold and only compound **JS27** displays a distinct structural feature. Because other analogues display similar *c* log *P* values with improved PAMPA permeability (up to 45 × 10^−6^ cm s^−1^; Table S1, ESI†), the observed increased permeability may be mostly a result of intramolecular interactions, such as hydrogen bonding, more than the lipophilic contribution, because the algorithm for *c* log *P* calculation does not consider 3D conformations. In addition, we measured particle sizes by using dynamic light scattering (DLS). Up to 95% of false positive readouts in high-throughput screens originate from colloidal aggregation.^34^ This phenomenon is driven by the physicochemical properties of the small molecule and buffer conditions. Generally, aggregates bind non-specifically to proteins, sequestering and denaturating them. Our data shows that, regardless of their limited solubility, compounds **JS24–JS27** do not form aggregates at the relevant inhibitory concentrations, which rules out unspecific binding to BMX (Table S1, ESI†).

To date, all the reported BMX inhibitors also display the ability to inhibit Bruton's tyrosine kinase (BTK). To determine if our leads were selective binders of BMX, we evaluated inhibitory capacity of our compounds against BTK. For the BTK IC_50_ assay (KinaseProfiler by Eurofins DiscoverX), we selected **BMX-IN-1**, the two analogues with higher BMX inhibitory capacity (**JS24** and **JS25**) and **JS27**, which presents the best *in silico* physicochemical profile, and also offers the possibility of derivatization. The results showed that all the leads are also potent BTK inhibitors, in the low nanomolar range (Fig. 1c). The same inhibitory trend is observed with an increase of 62-, 33- and 15-fold potency gain with **JS25**, **JS24** and **JS27**, respectively, relative to **BMX-IN-1**. Interestingly, in this assay **BMX-IN-1** displays 7-fold higher IC_50_ against BTK than BMX.

We aimed to modulate the physicochemical properties of the molecules to enhance the overall “drug-likeness” profile of the ligands. Ligand efficiency (LE) and lipophilic efficiency (LipE) are two important metrics that are associated with improved prospects for good drug properties (*e.g.* bioavailability) and are used as criteria for progression of the most promising candidates across drug discovery pipelines.^35^ LE is used to compare binding efficacy of inhibitors/ligands relative to their size, and LipE is used to compare binding efficacy by taking into consideration the lipophilicity of the molecules. With regards to BMX inhibition, compound **JS27** displayed a major LipE improvement relative to **BMX-IN-1** (5.54 *versus* 3.36) empowered by the drastic reduction in *c* log *P* as a result of the introduction of the aliphatic sulfonamide. Analogues **JS24**–**JS26** displayed mild LipE improvement (4.17, 4.43 and 4.45), which is mainly due to their structural similarity with **BMX-IN-1**. However, the LE improvement is mostly driven by the increased potency of all analogues rather than a decrease in the molecules’ size (Table 1). Similarly, the designed ligands offer a greater improvement of LE and LipE metrics with respect to BTK binding, relative to **BMX-IN-1**, driven also by a drastic potency gain.

**Table 1:** Ligand efficiency and lipophilic efficiency of **BMX-IN-1** and **JS24–JS27** against BMX and BTK

Compound	**LE (BMX)**	**LipE (BMX)**	**LE (BTK)**	**LipE (BTK)**
**BMX-IN-1**	0.26	3.36	0.23	2.50
**JS24**	0.29	4.17	0.29	4.00
**JS25**	0.30	4.43	0.30	4.22
**JS26**	0.30	4.45	Nd	Nd
**JS27**	0.30	5.54	0.29	5.29

Finally, we analyzed the pharmacophore diversity of the designed ligands against that of known BMX and BTK inhibitors. Ligand data was collected from ChEMBL v24, pre-processed as previously described^36^ and projected to the plane by means of a learning algorithm (Fig. 1d). It is apparent that BTK has been more often interrogated with small molecules (green) and that the studied chemotypes are more diverse in regard to topological pharmacophore arrangements relative to previously described BMX modulators (blue). Compounds **JS24–JS27** (yellow) focus on unexplored regions in BMX inhibitor space but overlaps with previously studied BTK chemotypes. Indeed, our compounds have shown potent activities against BTK, which is fully in line with the output of the learning algorithm. Altogether, our data shows that compounds **JS24–JS27** explore a new chemical space and provides a rationale to re-investigate and potentially repurpose BTK inhibitors as leads for future development of BMX ligands and *vice versa*.

### **JS24–JS27** show strong binding interactions with BMX

We further characterized the binding interaction between **JS24–JS27** and BMX by using differential scanning fluorimetry (DSF) and surface plasmon resonance (SPR). Purified recombinant human BMX protein (hBMX) was first subjected to thermal scanning in the absence and presence of **JS24–JS27**, and the respective protein melting temperature (*T*_m_) calculated. As shown in Table 2, **BMX-IN-1** increases the *T*_m_ value by 8.04 °C. Among the lead inhibitors, **JS24** displayed the highest stabilization, with an increase in Tm of 11.34 °C. Compounds **JS27** (10.81 °C), **JS26** (9.34 °C) and **JS25** (9.30 °C) showed high stabilization of the protein, which also suggests direct binding to BMX with a higher affinity relative to parent scaffold **BMX-IN-1** (Table 2 and Table S2, ESI†). The binding of **JS24–JS27** to BMX immobilized surfaces was then monitored in real-time by SPR assays (Table 2 and Fig. S3, Table S3, ESI†). **BMX-IN-1** was shown to bind to BMX with an affinity of *K*_D_ = 69 nM. It is important to note that it was not possible to accurately fit the dissociation rate constant of **BMX-IN-1** interaction with BMX because of the initial non-covalent binding event. In contrast, **JS24–JS27***K*_D_ values could not be determined due to the even higher prolonged off-rates, which were outside the range of the instrument specifications. The results suggest that compounds **JS24–JS27** have higher affinity interactions with BMX, showing comparable association rates (*k*_on_ from 5.4 × 10^4^–1.4 × 10^5^ M^−1^s^−1^) but, most importantly, very slow dissociation rates (*k*_off_ < 1 × 10^−4^ s^−1^), which is in agreement with the covalent nature of the interaction (Table 2).

**Table 2:** Melting temperature (*T*_m_) shift calculated with a DSF assay and kinetic constants calculated from SPR

Compound	Aveg. *T*_m_ (°C)	apo-BMX *T*_m_ (°C)	Δ*T*_m_ (°C)	*k*_on_/M^−1^ s^−1^	*k*_off_/s^−1^	*K*_D_/M
**BMX-IN-1**	60.17 ± 0.32	52.13 ± 0.11	8.04 ± 0.32	7.4 × 10^3^	5.10 × 10^−4^	6.9 × 10^−8^
**JS24**	63.57 ± 0.01	52.23 ± 0.06	11.34 ± 0.01	1.4 × 10^5^	<1 × 10^−4^	Nd^a^
**JS25**	61.43 ± 0.48	52.13 ± 0.11	9.30 ± 0.48	5.4 × 10^4^	<1 × 10^−4^	Nd^a^
**JS26**	61.47 ± 0.21	52.13 ± 0.11	9.34 ± 0.21	7.2 × 10^4^	<1 × 10^−4^	Nd^a^
**JS27**	62.94 ± 0.06	52.13 ± 0.11	10.81 ± 0.06	9.9 × 10^4^	<1 × 10^−4^	Nd^a^

a *K*_D_ not measured due to very prolonged off-rates (outside instrument specifications).

### **JS24–JS27** displays greater irreversible binding efficacy relative to BMX-IN-1

The inactivation of BMX occurs in a two-step process that is governed by two parameters: the affinity of the initial non-covalent binding, *K*_I_, and the rate of the subsequent covalent bond-forming reaction with the thiol of the cysteine residue, *k*_inact_. The rate of inactivation (*k*_inact_/*K*_I_) is a second-order event, which describes the efficiency of covalent bond formation,.^37^ Therefore, we evaluated the irreversible binding efficiency of our rationally designed compounds, as previously described.^38^ The kinetic analysis presented in Table 3, reveals that compound **JS25** exhibits the best binding fit with the target, with an inhibition rate constant of 323 pM. This represents an increase in excess of 10-fold relative to **BMX-IN-1** (*K*_I_: 4.07 nM). The other leads display a similar binding affinity among themselves (1.93–2.52 nM), lower than **JS25** and approximately 2-fold higher than **BMX-IN-1**. However, the rate of covalent bond formation of the bound inhibitor (determined by *k*_inact_) shows that compounds **JS24**, **JS25**, and **JS26** showed slightly improved efficiency (0.335, 0.378 and 0.443 min^−1^, respectively) in comparison to **BMX-IN-1** (0.217 min^−1^) and **JS27** (0.166 min^−1^). Consequently, the irreversible binding efficiency of **JS25** (19.4 μM^−1^ s^−1^) is the highest of the series, whereas **BMX-IN-1** shows the lowest result (0.89 μM^−1^ s^−1^) relative to the remaining inhibitors. Overall, these results provide quantitative evidence that the improved activity is mostly driven by changes in the binding complementarity between the compound and target rather than faster rate of covalent binding. Thus, taking into account that all the analogues have the same Michael acceptor moiety, the enhanced activity must be a result of the structural modifications introduced in the scaffold.

**Table 3:** Determination of the kinetic parameters *K*_I_, *k*_inact_, *k*_inact_/*K*_I_^a^

Compound	*K*_I_ [nM]	*k*_inact_ [min^−1^]	*k*_inact_/*K*_I_ [μM^−1^ s^−1^]
**JS25**	0.32 ± 0.05	0.378 ± 0.034	19.4 ± 1.55
**JS26**	1.93 ± 0.18	0.443 ± 0.003	3.86 ± 0.34
**JS24**	2.52 ± 0.01	0.335 ± 0.001	2.22 ± 0.01
**JS27**	2.15 ± 0.13	0.166 ± 0.003	1.29 ± 0.10
**BMX-IN-1**	4.07 ± 0.06	0.217 ± 0.005	0.89 ± 0.20^b^

a Results tested in duplicate, showing mean ± S.D.

b Value with a 0.06 μM^−1^ s^−1^ deviation from published results.^38^

### **JS24** covalently modifies cysteine 496 in BMX

**Fig. 2:**
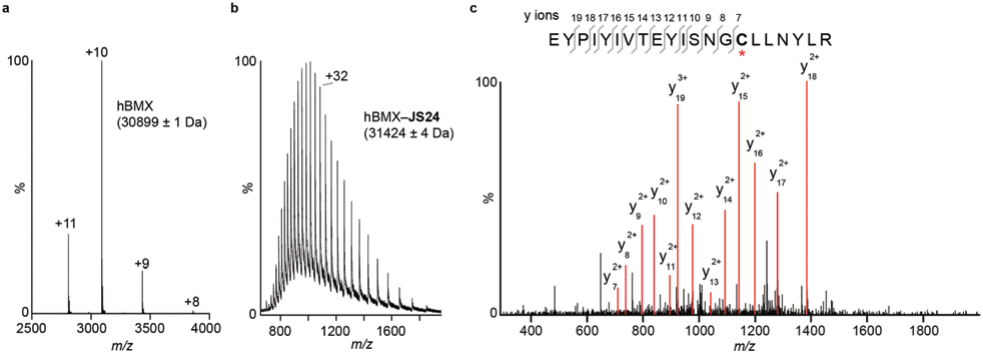
Mass spectrometric analysis of BMX and **JS24** conjugated to BMX. (a) Native MS analysis of hBMX. The measured molecular weight is indicated. (b) Denaturing MS analysis of drug-conjugated hBMX. The measured molecular weight is indicated. (c) Tandem MS analysis of the drug conjugated tryptic peptide of hBMX labelled on the sequence (top) and MS/MS mass spectrum (bottom). The red asterisk indicates the Cys site to which **JS24** is covalently linked.

Mass spectrometry (MS) studies confirmed the covalent binding of **JS24** at cysteine 496 of BMX. The truncated hBMX was analyzed by native MS and the protein mass found was 30899 Da (Fig. 2a). The protein was then treated with 2.5 mM of **JS24** at room temperature for 30 min in PBS pH 7.4 and directly analyzed by denaturing MS. The protein was fully denatured and cleaned on a reverse phase column, discarding any non-specific binding, to retain only any covalently linked compound. The mass found upon incubation with **JS24** is 31 424 Da, which is 525 Da larger than the apo-form of hBMX (Fig. 2b). This result suggests covalent conjugation of a single molecule of **JS24** to hBMX. Furthermore, MS/MS analysis after digestion of the drug conjugated hBMX indicates the covalent modification at cysteine residue 496 (Fig. 2c).

### The first X-ray structure of BMX with covalent inhibitor

**Fig. 3:**
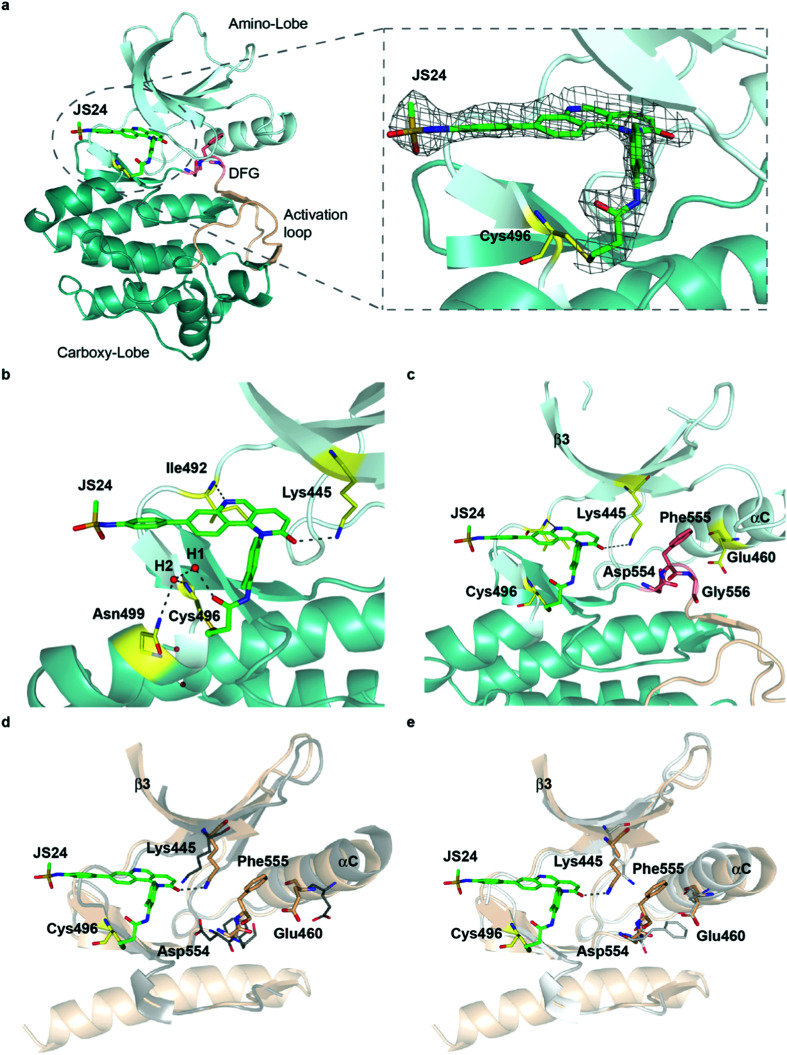
Crystal structure of the BMX-**JS24** complex (a). Representation of the co-crystal structure of BMX catalytic domain in complex with **JS24** (PDB code 6I99). The panel shows the well-defined electron density around the inhibitor, which is bound to BMX ATP binding pocket through a covalent bond with a cysteine residue (Cys496). (b) Non-bonding interactions of **JS24** in the ATP binding pocket. (c) BMX DFG-motif adopting the out-like conformation. (d) Analysis of the BMX-DFG^out-like^ motif conformation and BTK DFG^in^. (e) Analysis of the BMX DFG^out-like^ conformation with BTK DFG^out^.

To characterize the inhibition mechanism and binding mode of **JS24** to BMX at the atomic level, we tested a variety of commercial crystallization screens to obtain a protein crystal suitable for X-ray diffraction. Crystals were grown through co-crystallization of BMX protein at a final concentration of 10 mg mL^−1^, with a 2-fold molar excess concentration of inhibitor **JS24**, in a lead condition that consists of 0.2 M imidazole-malate buffer, pH 5.5, and 42% v/v PEG 600. The X-ray crystal structure of BMX in complex with inhibitor **JS24** was determined at 2.0 Å resolution (PDB ID: 6I99) with a well-defined electron density map around the BMX ATP binding pocket in which the inhibitor is bound (Fig. 3a). The values of the equivalent isotropic atomic displacement parameters for the ligand atoms within the pocket are comparable to those of the protein atoms they are interacting with, an indication of full ligand occupancy of the binding site. Not surprisingly, an increase is observed in the sulfonamide aromatic ring because this group is more exposed to the solvent and hence more mobile.

The crystal structure shows the expected covalent binding between the acrylamide warhead and Cys496. Other major interactions of the inhibitor with the enzyme active site are mediated through hydrogen bonds between the nitrogen in the quinoline ring and Ile492, and between the Lys445 and the oxygen located in the fused pyridinone ring (Fig. 3b). The hydrogen bond between **JS24** and Lys445 is actually one of the key points to regulate BMX activity. The conserved β3 Lys interacts with αC-helix Glu residue to form a salt bridge required for ATP catalysis. The binding of **JS24** to Lys445 alters this interaction between the β3 Lys and the αC-helix Glu and consequently inactivates BMX. The aromatic rings of **JS24** are engaged in CH/π interactions with the side chains of Tyr491, Ala443, Val431, and Leu543 (Fig. S4, ESI†). Compound **JS24** is further stabilized by a hydrogen bond between a water molecule (W1) and the carbonyl oxygen of the acrylamide group. A second water molecule (W2) stabilizes the first (W1) through a hydrogen bond, and forms hydrogen bonds with the peptide nitrogen of Cys496 and the terminal amide group of Asn499 (Fig. 3b).

The crystal structure also shows that the DFG-motif adopts an out-like conformation (Fig. 3c) in which the Asp554 side chain is positioned in the back cleft, away from the ATP binding pocket, and the Phe555 aromatic ring points up into the gatekeeper region blocking the β3 Lys445-αC Glu460 ion pair formation. Both the activation loop and the DFG-out-like conformation are similar to what is observed in the only reported BMX crystal structure with non-covalent inhibitors Dasatinib and PP2.^39^ The positioning of the BMX DFG-motif is reminiscent of an inactive conformation or DFG-out, typically found in BTK and other kinases inactive structures,^40^ and it is also commonly observed in type II inhibitor complexes.^41^ In the apo-BTK (PDB: 3P08), the DFG-in Asp539 rotates towards the ATP binding pocket to chelate magnesium and the DFG-in Phe540 is positioned in the back cleft to allow the formation of the β Lys430-αC Glu445 ion pair, which is important for catalysis. In BMX/**JS24** complex the DFG-out-like Asp554 points down and away from the ATP binding pocket and the Phe555 swings up to block the ion pair formation (Fig. 3d). However, relative to the DFG-out-like structure in BMX/**JS24** complex with a BTK DFG-out structure (PDB: 3PIY), both structures display complete rotation of the DFG-aspartate residue away from the ATP binding pocket. Only the BTK DFG-out Phe540 residue rotates away from the core of the protein and towards the ATP binding pocket to create a back pocket capable of accepting an aromatic moiety (Fig. 3e).

Finally, the positioning of the sulfonamide aromatic ring is also of utmost importance. This group does not interact with any important residue and it is in fact pointing out of the ATP pocket (Fig. S4, ESI†). Interestingly, this feature would allow for the installation of a linker or chemical handle in this region of the molecule without altering significantly the inhibitor binding capacity of the lead compound.

### Molecular dynamics (MD) simulations on BMX covalently linked to **JS24** and **JS27**

**Fig. 4:**
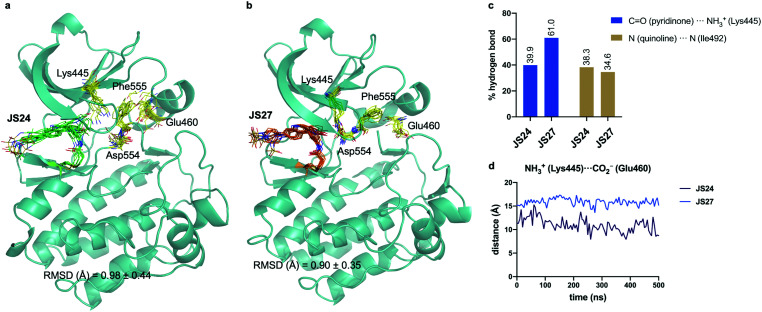
(a and b) Structural ensembles derived from 0.5 μs MD simulations on BMX covalently linked to **JS24** and **JS27**. (c) Population of the most relevant hydrogen bonds between the ligands and BMX derived from the MD simulations. (d) Evolution of the Lys445–Glu460 distance along the MD simulations of both complexes. See also Videos 1 and 2 (ESI†). **JS24**, **JS27**, Lys445, Glu460, Asp554 and Phe555 are shown as sticks. The protein is shown as ribbons and only the first conformer (time = 0 ns) is shown for clarity.

We performed then 0.5 μs MD simulations on BMX covalently linked to **JS24** (Fig. 4a and b). Computational details can be found in the ESI.† According to these calculations, the binding mode found in the X-ray structure is retained in solution. Both hydrogen bonds releveled by the crystallographic studies, one between the backbone of Ile492 and the nitrogen atom of the quinoline and the other one between the side chain of Lys445 and the carbonyl oxygen of the quinoline ring, are populated in the complex (Fig. 4c). Also, as it occurs in the X-ray structure, **JS24** is engaged in CH/π interactions with the side chains of Val431, Ala443, Tyr491 and Leu543 (Fig. S5, ESI†). The MD calculations show that the DFG-motif also adopts an out-like conformation in solution (Fig. 4a). Thus, Phe555 precludes the ion pair formation between Lys445 and Glu460, which is supported by the distance between the side chains of Lys455 and Glu460 (Fig. 4d). Asp554 side chain is located away from the ATP binding pocket (Fig. 4a). The good agreement between the X-ray and the structure proposed by the simulations prompted us to propose a 3D-model for BMX linked to ligand **JS27**. To this purpose, **JS27** covalently linked to Cys496 was superimpose on the X-ray structure of the BMX/**JS24** complex. and used as starting structure in the simulations. The simulations show that **JS27** adopts a similar pose in the binding site than **JS24** (Fig. 4b). Thus, **JS27** is engaged in the same hydrogen bonds with the receptor as in BMX/**JS24** complex, with populations close to 61.0% and 34.6% for the interactions involving Lys445 and Ile492, respectively. The aromatic system of the ligand interacts with the hydrophobic residues Val431, Ala443, Tyr491 and Leu543 through hydrophobic interactions (Fig. S5, ESI†). As in BMX/**JS24** complex, the binding of **JS27** impedes the ion pair Lys445-Gl460 formation (Fig. 4d). Significantly, in both complexes, the sulfonamide group of the ligands does not stablish any contact with the receptor and is exposed to the solvent.

### **JS25** is a selective TEC family inhibitor

Most of BMX inhibitors reported to date offer poor selectivity because they are both BMX and BTK inhibitors. Their cellular effect is often attributed to off-target activity either upstream or downstream of BMX signaling pathways. To investigate in which targets the new inhibitors could have an effect we tested potent inhibitor **JS25** against a panel of 36 BMX-related kinases in the Eurofins DiscoverX's KINOMEscan™ platform at a concentration of 1 μM.

It is important to note that from the extensive number of accessible cysteine residues distributed across the whole kinome not all are available for covalent modification.^12–14^ BMX belongs to a restricted group that includes 10 other kinases that share an equivalently placed cysteine in the ATP binding pocket. This group comprises members from the TEC family (BTK, ITK, TXK and TEC), the EGFR family (EGFR, Her2, Her4), JAK3, BLK and dual specificity mitogen-activated protein kinase 7 (MAP2K7). Therefore, we included the whole TEC, EGFR and JAK family in our screening, and the Src family and Lkb1, which also have a cysteine within the same sequence alignment. We also included kinases involved in upstream (Src, FAK, PI3K, mTOR, PDK1) and downstream (Akt, PAK1, TAM) regulation of BMX signaling pathway and non-receptor tyrosine protein kinase Abl. The KinomeScan platform is a binding assay and the screening showed that **JS25** displays a strong binding affinity against all the members of TEC family that share an equivalently placed cysteine and within these, higher affinity is observed towards BMX, BTK and TEC (Table 4).

**Table 4:** Kinase selectivity of **JS25** over 36 BMX-related kinases in the KINOMEscan™ platform. The results for primary screen binding interactions at 1 μM concentration are reported as % DMSO control

Family	Target	% Ctrl	Family	Target	% Ctrl
TEC	BMX	1.3	Src	FYN	99
	BTK	0		SRC	92
	ITK	4.7		YES1	85
	TEC	0.4		BLK	16
	TXK	3.4		FGR	93
EGFR	EGFR	87		LCK	80
	ERBB2	89		HCK	95
	ERBB3	91		LYN	100
	ERBB4	66	mTOR	MTOR	100
JAK	JAK1(JH1domain-catalytic)	93	Liver Kinase B1	STK11	52
	JAK2(JH1domain-catalytic)	81	Pkb	AKT1	100
	JAK3(JH1domain-catalytic)	21		AKT2	94
	TYK2(JH1domain-catalytic)	100		AKT3	99
FAK	PTK2	93	PAK1	PAK1	100
PI3K	PIK3CA	79	TAM	AXL	93
	PIK3CB	89		MERTK	89
	PIK3CG	64	Abl	ABL1-phosphorylated	100
	PIK3CD	100	PDPK1	PDPK1	92

As stated above, the TEC family has high sequence similarity and in particular residues in the ATP binding kinase domain share 40–65% identity and 60–80% similarity. The ATP binding sites are also highly conserved between the TEC and Src families with 14 identical residues out of 18 that comprise the ATP binding pocket. More specifically, BMX shares a 57% similarity to Src and most importantly, one of the key determinants of kinase selectivity – the gatekeeper residue – is a Thr in both the Src family and the TEC family members except ITK.^39^ It is therefore not surprising that **JS25** also binds Blk (and JAK3) whereas no affinity was observed with other potential targets. These results reveal **JS25** as a selective probe for TEC kinases and suggest that any cellular activity mediated by **JS25** is probably a result of inhibition of any of the TEC kinases rather than any off-target inhibition of upstream and downstream BMX regulators.

### Intracellular BMX inhibition and degradation by **JS25**

**Fig. 5:**
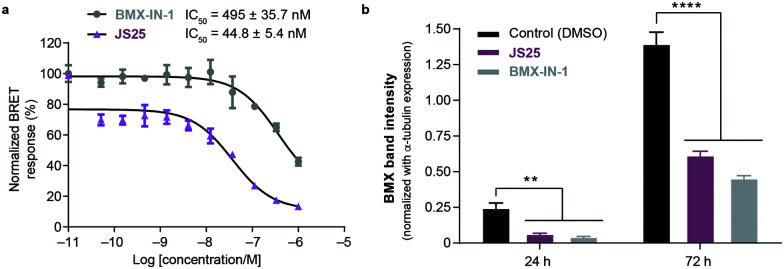
(a) Intracellular target engagement in HEK293 cells transiently transfected with BMX expressing NanoLuc®-BMX fusion vector with Promega's NanoBRET™ TE Intracellular Kinase Assay. Assay performed at Reaction Biology Corporation (USA), with concentrations tested in duplicate, showing mean ± S.D. Cells were treated for 1 h and IC_50_ values were calculated and plotted by using GraphPad Prism 8 based on a sigmoidal dose response curve. (b) **JS25** (10 μM) and **BMX-IN-1** (10 μM) induce degradation of wild-type BMX in PC3 cells. Sampling was taken after 24 h and 72 h of incubation with **JS25** and **BMX-IN-1**. Band intensity was measured using ImageJ and normalized with α-Tubulin band intensity. Differences between groups were revealed through 2way-ANOVA. Data are mean ± standard deviation obtained from at least three independent measurements (*n* = 3). See ESI† for additional data and analysis.

To validate target affinity and identification for **JS25**, we performed an intracellular target engagement kinase assay with HEK293 cells expressing NanoLuc®-BMX fusion vector with Promega's NanoBRET™ TE Intracellular Kinase Assay. The cell proliferation depends on BMX kinase activity that was used to monitor the cellular activity of the compounds (IC_50_). As shown in Fig. 5a, the IC_50_ determination showed the inhibitory capacity of **JS25** (IC_50_: 44.8 nM) is 10 times greater than **BMX-IN-1** (IC_50_: 495 nM), which aligns with the previous observations of an increased biochemical potency with similar activity difference. We next investigated whether the treatment of wild-type PC3 cells with **JS25**, as well as **BMX-IN-1** (as a control), would induce degradation of BMX. After 24 and 72 h, it was possible to verify that the level of expressed BMX protein in PC3 cells decreased upon treatment with **JS25** as well as with the control molecule (Fig. 5b and Fig. S11, ESI†). This data indicates that **JS25** is able both inhibit catalytic activity and degrade BMX it in cells.

### Cancer cell growth inhibition by BMX inhibitors

The role of BMX in different pathologies is not yet fully validated. Nevertheless, it has been implicated in many regulatory mechanisms and despite the absence of a BMX dependent disease model, prostate cancer cell lines have been used to evaluate anti-proliferative effects of the inhibitors in a cellular context. In a previous experiment (unpublished results) we screened several inhibitors from Table S1 (ESI†) in a collection of cell lines representing prostate, brain, blood, breast, ovary, lung, bone marrow and lymphoid tumour tissues. Compounds were incubated with cells for 72 h in a 386 well-plate format to monitor dose-dependent impact on viable cell growth by using the CellTiter-Glo® luminescent assay, which quantifies ATP and the presence of metabolically active cells. The study included **JS24**, **BMX-IN-1** and the structurally similar compounds **JS10** and **JS11** which do not bind to BMX (Fig. S1 and Table S1, ESI†).

The results presented in Table 5 show that **JS10** and **JS11** (non-binders) have little or no effect on viable cell growth of the majority of the tested cell lines. **BMX-IN-1** demonstrated more potent inhibitory effects relative to **JS24** in the four prostate cancer cell lines that were included in the panel, 22RV1, PC3, LNCaP and DU145, particularly in those dependent on androgen receptor signaling (LNCaP and 22RV1). In contrast, androgen receptor negative cells (DU145 and PC3) were overall more resistant to treatment. In addition, **JS24** showed potent inhibitory effects against LNCaP and 22RV1 but also against PC3, which are androgen receptor negative cells. Furthermore, both compounds were also potent inhibitors of viable cell growth for RS4 (11) (lymphoblast) and DAUDI (T-lymphoblast) cells, in which BTK is highly overexpressed. Altogether, these results demonstrate BMX inhibition impacts viable cell growth of prostate cancer cells and prompted us to investigate further the importance of the androgen receptor and related BMX pathways in these cell lines.

**Table 5:** Viable cell growth inhibition of compounds **BMX-IN-1**, **JS10**, **JS11** and **JS24** in a panel of prostate, brain, blood, breast, ovary, lung and lymphoid cancer cells^a^

Tissue	Cell line	**BMX-IN-1**	**JS24**	**JS10**	**JS11**
Prostate	LNCaP	1.81 ± 0.05	4.4 ± NC	9.7 ± NC	10.41 ± NC
	22 RV1	2.07 ± 0.06	6.66 ± 0.09	4.86 ± 0.11	7.3 ± NC
	PC3	10.98 ± 1.13	4.8 ± NC	ND	20.12 ± NC
	DU145	17.7 ± NC	ND	ND	ND
Brain	U-87MG	5.33 ± 0.19	5.04 ± 0.01	ND	ND
	SK-N-MC	2.36 ± NC	8.53 ± 0.44	11.19 ± NC	8.24 ± NC
Blood	Jurkat	5.99 ± NC	5.48 ± ND	9.71 ± 1.48	6.36 ± 0.17
	Kasumi	3.13 ± 0.06	5.12 ± 0.12	4.37 ± 0.04	10.14 ± 0.07
Breast	MDA-MB-231	23.61 ± 0.48	ND	ND	ND
Ovary	CAOV3	7.68 ± 0.13	8.56 ± NC	17.30 ± NC	19.31 ± NC
	OVCAR3	ND	ND	ND	ND
Bone marrow	H1299	ND	7.28 ± 0.37	19.42 ± NC	ND
Lung	RS4(11)	1.176 ± 0.06	2.09 ± NC	5.06 ± NC	6.66 ± NC
Lymphoid	DAUDI	1.68 ± 0.07	1.27 ± 0.05	2.57 ± 0.09	4.57 ± 0.12

a Viable cell growth was measured after 72 h incubation in 386 well-plate format. GI_50_ values were tested in triplicate and are reported as the mean ± SD in μM. ND, non-determined, no growth inhibition observed within the concentrations tested. NC, non-calculated. When ambiguous fit was observed curves were top (100%) and bottom (0%) constrained and GI_50_ was determined with 4-P least squares fit. In these cases, SD is not calculated by GraphPad Prism 8.

### Androgen-receptor positive prostate-cancer cells are sensitive to **JS24–JS27**

**Fig. 6:**
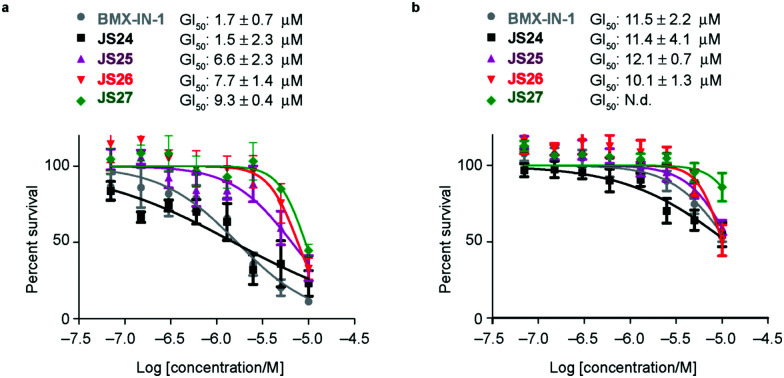
Anti-proliferative activity of compounds **BMX-IN-1** and **JS24–JS27** against LNCaP (a) and PC3 (b) prostate-cancer cell lines. Briefly, proliferation in LNCaP and PC3 cells was measured after 96 h incubation, in 96-well plates with **BMX-IN-1** and **JS24–JS27**. GI_50_ values are reported in μM and are the mean of three individual experiments performed in triplicate. N.d., non-determined, no growth inhibition observed within the concentrations tested.

Based on the results showed in the previous section, we tested the ability of compounds **JS24–JS27** to inhibit the proliferation of LNCaP and PC3 prostate cancer cell lines by using CellTiter-Glow®. The androgen-receptor negative PC3 cells are resistant to the treatment, with no significant anti-proliferative effect at the maximum concentration tested (10 μM). With androgen-receptor positive cells LNCaP a different profile was observed. **BMX-IN-1** and **JS24** showed a GI_50_ of 1.7 and 1.5 μM, respectively. Compound **JS27** was the least active (GI_50_ 9.3 μM), and **JS25** and **JS26** inhibited proliferation with a GI_50_ of 6.6 and 7.7 μM, respectively, as shown in Fig. 6.

To determine whether the growth inhibition was due to apoptosis, we carried out fluorescence-assisted cell sorting analysis with propidium iodide staining. LNCaP cells were incubated with **BMX-IN-1** and **JS24–JS27** for 64h, at 10 μM and results showed that no marked differences in the percentage of necrotic events relative to the vehicle control, which suggests that in these conditions these molecules do not enhance cell death (Fig. S6, ESI†). It is not surprising that all these compounds show only moderate proliferation inhibitory potential in prostate-cancer cell lines and it remains questionable whether modulation of BMX *per se* is relevant or not, towards anti-proliferative effects.^33^ In fact, a large body of evidence in the literature shows that selective or dual BMX/BTK inhibitors have poor anti-proliferative effects in BMX-dependent models, most probably from dynamic compensation of signaling mechanisms. Focus has been placed on the modulation of BMX activity to sensitize prostate-cancer cells to other therapeutic agents because anti-proliferative effects are only observed in combination with inhibitors of related pathways.^42^**BMX-IN-1** growth inhibition of RV-1 cells could only be potentiated with the Akt inhibitor MK2206;^33^ ABT-737, a non-covalent inhibitor only induces apoptosis upon co-treatment with PI3K inhibitors;^28^ the dual BMX/BTK inhibitor CTN06 requires co-treatment with autophagy inhibitor chloroquine (CQ) or docetaxel to inhibit PC3 cells growth^43^ and a similar profile is observed with the dual BMX/Src inhibitor CTA095 to synergize with CQ and paclitaxel.^44^

The activation of BMX in response to PI3K signaling is just one of the mechanisms through which the levels of BMX become increased in prostate cancer.^43,45^ A very recent study^46^ showed that BMX expression in prostate cancer is suppressed directly through androgen receptor as a result of binding to BMX. Consequently, BMX expression rapidly increases in response to androgen deprivation therapy which enhances tyrosine kinase signaling and the subsequent emergence of castration-resistant prostate cancer. This study further highlights the potential use of BMX inhibitors in combination therapy, in this case in combination with AR targeting. To further validate this hypothesis and assess the effect of our inhibitors with other drugs, we performed a co-treatment regimen with known inhibitors of related upstream and downstream pathways.

### Co-treatment of LNCaP cells with **JS24–JS26** and androgen receptor antagonist, PI3K and AKT inhibitors

**Fig. 7:**
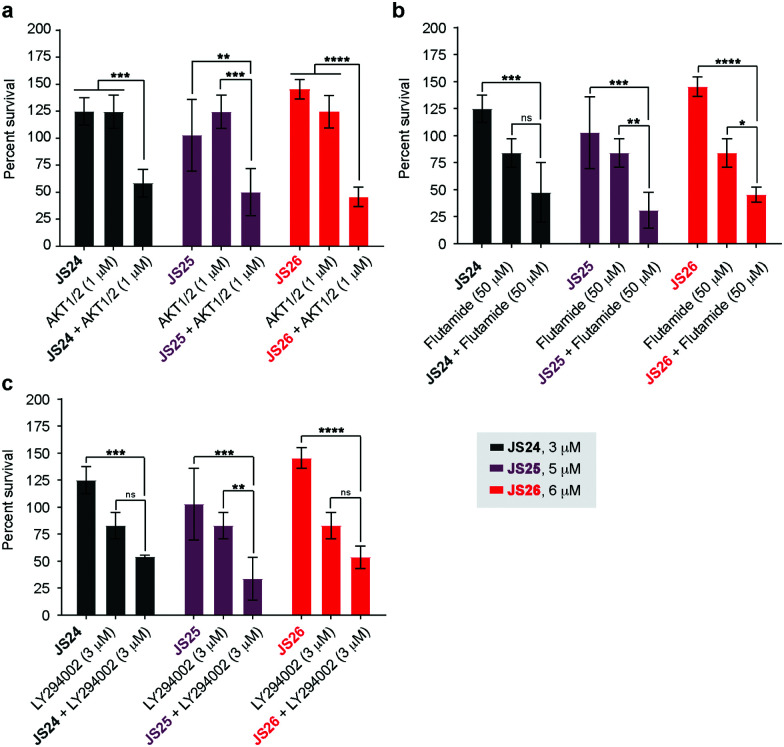
Anti-proliferative activity in LNCaP cells of compounds **JS24**–**JS26** in combination with **AKT1/2** (AKT inhibitor), **Flutamide** (androgen receptor antagonist) and **LY293002** (PI3K inhibitor). (a) Cells co-treated with **JS24** (3 μM), **JS25** (5 μM) and **JS26** (6 μM) with **AKT1/2** (1 μM); (b) **JS24** (3 μM), **JS25** (5 μM) and **JS26** (6 μM) with **Flutamide** (50 μM); (c) **JS24** (3 μM), **JS25** (5 μM) and **JS26** (6 μM) with **LY294002** (3 μM). Values are reported in % cell viability normalized to DMSO controls and are the mean of three individual experiments performed in triplicate. Determined *P*-values are illustrated as ns (*P* > 0.05), * (*P* ≤ 0.05), ** (*P* ≤ 0.01), *** (*P* ≤ 0.001) and **** (*P* ≤ 0.0001).

As shown above, BMX inhibition alone induces limited cell death in BMX-expressed cell lines owing to the existence of compensatory mechanisms in signaling pathways. To evaluate whether our BMX inhibitors could potentially be used in combination treatment regimens, we sought to look at the synergistic anti-proliferative effects of BMX inhibitors when combined with other therapeutic agents, which pre-sensitize prostate cancer cells. For this purpose, LNCaP cells were co-treated in a combinatorial fashion with compounds **JS24–26**, **AKT1/2** (AKT inhibitor), **Flutamide** (androgen receptor antagonist) and **LY294002** (PI3K inhibitor). Cell viability was evaluated after 5 days with CellTiter-Glo® and compared with the overall anti-proliferative effects of the compounds alone. An optimization study was performed by screening several concentrations (Fig. S7, ESI†) to determine the ideal conditions to obtain initial viability above 80% with the individual inhibitors alone (Fig. S8, ESI†). Based on these results, we tested **JS24** (at 3 μM), **JS25** (5 μM) and **JS26** (6 μM) with **AKT1/2** (1 μM), **Flutamide** (50 μM) and **LY294002** (3 μM). Results are shown in Fig. 7.

Although the control concentrations of **JS24–26** and the inhibitors did not have an effect on reducing cell viability upon co-treatment, a marked viability decrease was observed in all tested conditions. With **AKT1/2** a decrease in cell viability ranging from 48% (with **JS24**) to 63% (with **JS26**) was observed, relative to control **AKT1/2**. With **Flutamide**, the most effective combination was with compound **JS25** (63% cell viability reduction) and the least effective with **JS24** (44% reduction). Finally, co-treatment with **LY294002** decreased cell viability by 35% (with **JS24** and **JS26**) and 59% (with **JS25**). Overall, these results demonstrate a synergistic effect between **JS24-26** and **AKT1/2**, **Flutamide** and **LY294002** in cancer cell proliferation capable of overcoming the compensatory mechanisms of BMX inhibition, and open the possibility of becoming useful molecules for drug combination approaches.

## Conclusions

We explored a chemical scaffold that contains an archetypal tricyclic core of a quinoline with a fused pyridinone, which is present in BMX covalent inhibitor **BMX-IN-1**. We sought to introduce a chemical handle that may be used for further derivatization whilst simultaneously tuning the physicochemical properties. We found that rational modification introduced at the position 7 of the quinoline moiety leads to potent, single-digit nanomolar inhibition of BMX and BTK. Topological pharmacophoric features are outside the chemical space previously explored in BMX inhibition, and afford molecules with more favorable physicochemical profiles with a reduced *c* log *P* and increased permeability (**JS26** and **JS27**). We also unveiled the X-ray crystal structure of BMX with a covalent inhibitor (**JS24**), which shows the protein with the “DFG-out like” motif typical of an inactive conformation. The crystal structure also shows that the “tail substituent”, opposite to the acrylamide warhead points outside the ATP pocket, which suggests an exit vector for further derivatizations. A comparable pose for ligands **JS24** and **JS27** were proposed by extensive MD simulations performed on the complexes with BMX.

We determined the rate of covalent modification and, to our knowledge, this is the highest value reported in the literature. The kinetic analysis showed that this is mostly driven from the potency of the first reversible binding event (*K*_I_ = 323 pM) and shows that our rational design afforded a preferred fit for the BMX binding pocket. In a cellular context, most potent compound **JS25** also showed low nanomolar potency in a target engagement assay (45 nM) in BMX-dependent cells (transfected HEK293) which is 10-fold superior to the reference ligand. Treatment of PC3 cells with **JS25** also led to degradation BMX. Furthermore, all lead compounds displayed anti-proliferative effects in androgen-receptor positive prostate cancer cells that where further increased when combined with known inhibitors of related signaling pathways, further highlighting the potential of combinatorial effects with BMX-related pathways.

As stated above, selectivity among members of the TEC family is hard to achieve. Interestingly, available data shows that therapeutically active drugs are not selective molecules. Ibrutinib, developed as a covalent BTK inhibitor, has been approved by the FDA for the treatment of chronic lymphocytic leukemia, mantle cell leukemia (MCL) and Waldenström macroglobulinemia and is currently in multiple clinical trials because it has proved efficacy in different indications, such as non-small cell lung cancer and autoimmune diseases.^7,47,48^ With a broad selectivity profile, Ibrutinib inhibits the whole TEC family, EGFR, JAK3, Her2, Blk and Itk kinases. Acalabrutinib, a second generation BTK inhibitor that was also granted FDA approval for MCL is more selective, and only inhibits BTK, TEC, BMX and TXK.^49^ Other BTK inhibitors in clinical development (Spebrutinib, Zanubrutinib and Tirabrutinib) also display a broad selectivity for kinases with a cysteine as the Cys496 residue in BMX,^50^ consequently, it is reasonable to extrapolate that the compounds developed here can become therapeutically useful as BMX inhibitors and find application in other TEC-related B-cell malignancies. The most potent compound **JS25** also has a multi-target profile and is active against all five TEC kinases, JAK3 and BLK. As such, we are currently evaluating the utility of these new molecules in B-cell related lymphocytic diseases where TEC-kinases play a prominent role.^17,51^

## Conflicts of interest

J. D. S. and G. J. L. B. are inventors in a patent application related to the findings reported in this manuscript. Other authors declare no competing interests.

## Supplementary Material

CB-001-D0CB00033G-s001

CB-001-D0CB00033G-s002

CB-001-D0CB00033G-s003
