# Supplementary material for: Structural and biophysical insights into the mode of covalent binding of rationally designed potent BMX inhibitors
Source: RSC Chem Biol. 2020 Aug 28;1(4):251–62. doi: 10.1039/d0cb00033g (PMC8341910; doi:10.1039/d0cb00033g)
Supplement: CB-001-D0CB00033G-s001 [file CB-001-D0CB00033G-s001.pdf]

Electronic Supplementary Material (ESI) for RSC Chemical Biology.
This journal is © The Royal Society of Chemistry 2020

ELECTRONIC SUPPLEMENTARY INFORMATION for

# Structural and biophysical insights into the mode of covalent binding of rationally designed potent BMX inhibitors

João D. Seixas,[*a,#] Bárbara B. Sousa,[a,b#] Marta C. Marques,[a] Ana Guerreiro,[a] Rui Traquete,[a] Tiago Rodrigues,[a] Inês S. Albuquerque,[a] Marcos F. Q. Sousa,[b,c] Ana R. Lemos,[b,c] Pedro M. F. Sousa,[b,c] Tiago M. Bandeiras,[b,c] Di Wu,[d] Shelby K. Doyle,[e] Carol V. Robinson,[d] Angela N. Koehler,[e] Francisco Corzana,[f] Pedro M. Matias,[b,c] and Gonçalo J. L. Bernardes[*a,g]

Correspondence should be sent to: gb453@cam.ac.uk or joaoseixas@medicina.ulisboa.pt

**TABLE OF CONTENTS**

1. Supporting Figures, Schemes and Tables S2
2. Synthesis S20
3. Methods S63
4. References S74

# 1. SUPPORTING FIGURES, SCHEMES AND TABLES


| | | |
|---|---|---|
| **Figure S1** | Structure of the analogues prepared | S3 |
| **Figure S2** | Tanimoto coefficients | S4 |
| **Figure S3** | Biochemical IC50 determination of compounds **JS9–29** | S5 |
| **Figure S4** | Sensorgrams of SPR experiments with **BMX-IN-1**, **JS9C**, **JS9D**, **JS9E**, **JS10**, **JS12**, **JS24–27** | S6 |
| **Figure S5** | Co-crystal structure exhibiting the binding mode of **JS24** to BMX kinase catalytic domain and highlight of hydrophobic interactions | S7 |
| **Figure S6** | Distance distributions derived from MD simulations for ligand **JS24** and **JS27** with key residues of BMX | S8 |
| **Figure S7** | Flow Cytometry analysis of **BMX-IN-1** and analogues **JS24–27** on apoptosis in LNCaP prostate cancer cells | S9 |
| **Figure S8** | Anti-proliferative effects of **JS24-26**, AKT inhibitor **AKT1/2,** androgen receptor inhibitor **Flutamide** and PI3K inhibitor **LY294002** on LNCaP prostate cancer cells. | S9 |
| **Figure S9** | Synergistic anti-proliferative effects of **JS24-26** with AKT inhibitor **AKT1/2**, androgen receptor inhibitor **Flutamide** and PI3K inhibitor **LY294002** on the viability of LNCaP prostate cancer cells. | S10 |
| **Figure S10** | Kinase selectivity of compound **JS25** over 36 BMX-related kinases in the Eurofins DiscoverX's KINOMEscan platform | S11 |
| **Figure S11** | BMX degradation after treatment with **JS25** and **BMX-IN-1** in PC3 cells | S12 |
| **Scheme S1** | Synthetic route for the preparation of compounds **JS9a–e**, **JS10–13** and **JS14–23** | S13 |
| **Scheme S2** | Synthetic route for the preparation of compounds **JS24–27** | S14 |
| **Scheme S3** | Synthetic route for the preparation of compound **JS28** | S14 |
| **Scheme S4** | Synthetic route for the preparation of compound **JS29** | S14 |
| **Table S1** | *In silico* cLogP and LogS calculation and *in vitro* artificial membrane permeability (PAMPA) and colloidal aggregation (DLS) determination | S15 |
| **Table S2** | Melting temperature (Tm) shift calculated with a DSF assay | S16 |
| **Table S3** | Kinetic constants calculated from Surface Plasmon Resonance | S17 |
| **Table S4** | Data processing statistics for the crystallographic structure of BMX in complex with inhibitor **JS24** | S17 |
| **Table S5** | Refinement statistics for the crystallographic structure of BMX in complex with inhibitor **JS24** | S19 |

**Figure S1.** Structure of the analogues prepared. The modified regions in each analogue are highlighted in red.

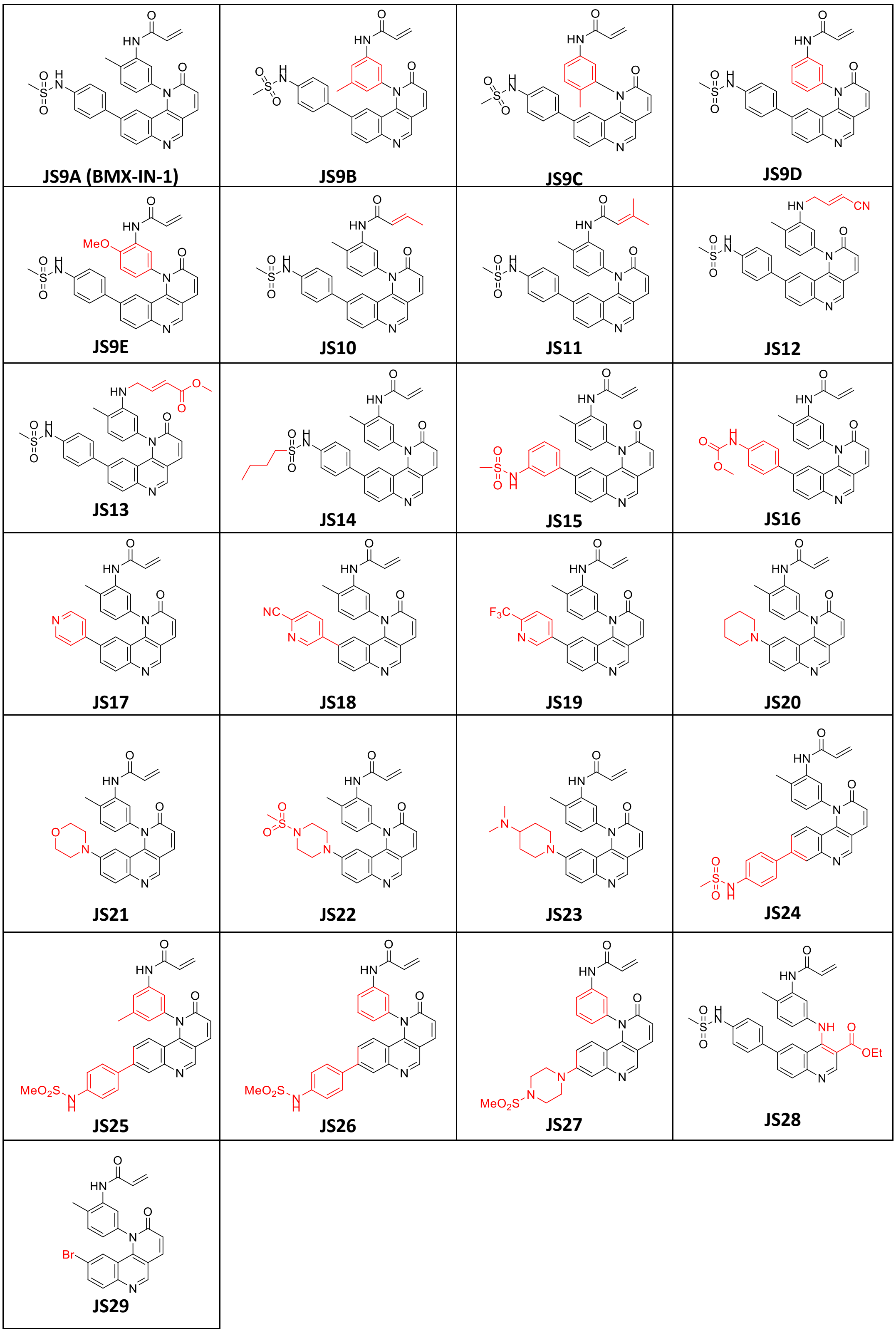

**Figure S2.** Tanimoto coefficient (ECFP4 fingerprint) between compound **JS24** and the **BMX** inhibitors in ChEMBL v27. Data show the general structural dissimilarity of a prototypical JS compound.

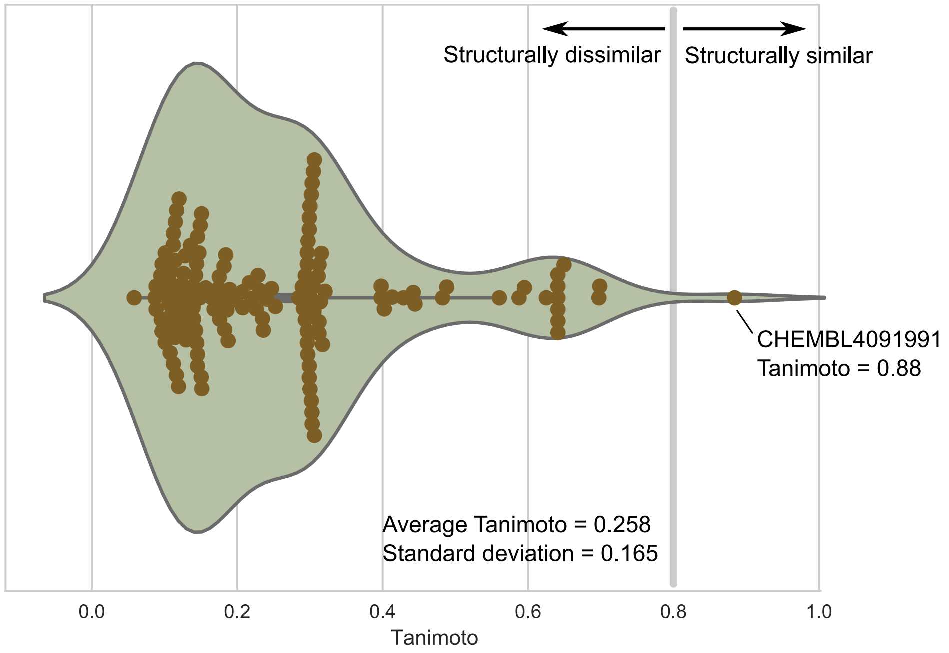

**Figure S3**. Biochemical $IC_{50}$ determination of compounds **JS9–29**. Human recombinant BMX was incubated with the compounds and phosphorylation of a biotinylated peptide measured by HTRF. **TOP**: The full library was tested in 4 different experiments, where **BMX-IN-1** was always used as control (JS9A was prepared in house and also tested as control; BMX-IN-1 indicates commercial source of the inhibitor). **BOTTOM**: Values expressed in potency gain or loss (fold) against control BMX-IN-1 used in each set of experiments. Compound **JS10** had a potency loss of 58-fold, while compounds **JS11–13** had a potency loss of more than 275-fold.

| Experiment 1 | | Experiment 2 | | Experiment 3 | | Experiment 4 | |
|---|---|---|---|---|---|---|---|
| **Cpd** | **$IC_{50}$ (nM)** | **Cpd** | **$IC_{50}$ (nM)** | **Cpd** | **$IC_{50}$ (nM)** | **Cpd** | **$IC_{50}$ (nM)** |
| JS9A | 360±24 | BMX-IN-1 | 55±35 | BMX-IN-1 | 230.2±34.5 | BMX-IN-1 | 50.2±0.7 |
| JS9B | 240±25 | JS14 | 890±27 | JS15 | 118.7±11.4 | JS24 | 7.5±0.4 |
| JS9C | 6900±2925 | JS16 | 50±62 | JS22 | 93.8±0.87 | JS25 | 3.5±0.02 |
| JS9D | 59±5.0 | JS17 | 45±10 | | | JS26 | 9.1±0.3 |
| JS9E | 1300±206 | JS18 | 140±12 | | | JS27 | 13.7±0.7 |
| JS10 | 21000±7250 | JS19 | 470±179 | | | | |
| JS11 | NC | JS20 | 42±13 | | | | |
| JS12 | NC | JS21 | 43±5 | | | | |
| JS13 | NC | JS23 | 41±6 | | | | |
| | | JS24 | 4.1±0.3 | | | | |
| | | JS28 | 410±35 | | | | |
| | | JS29 | 44±2 | | | | |

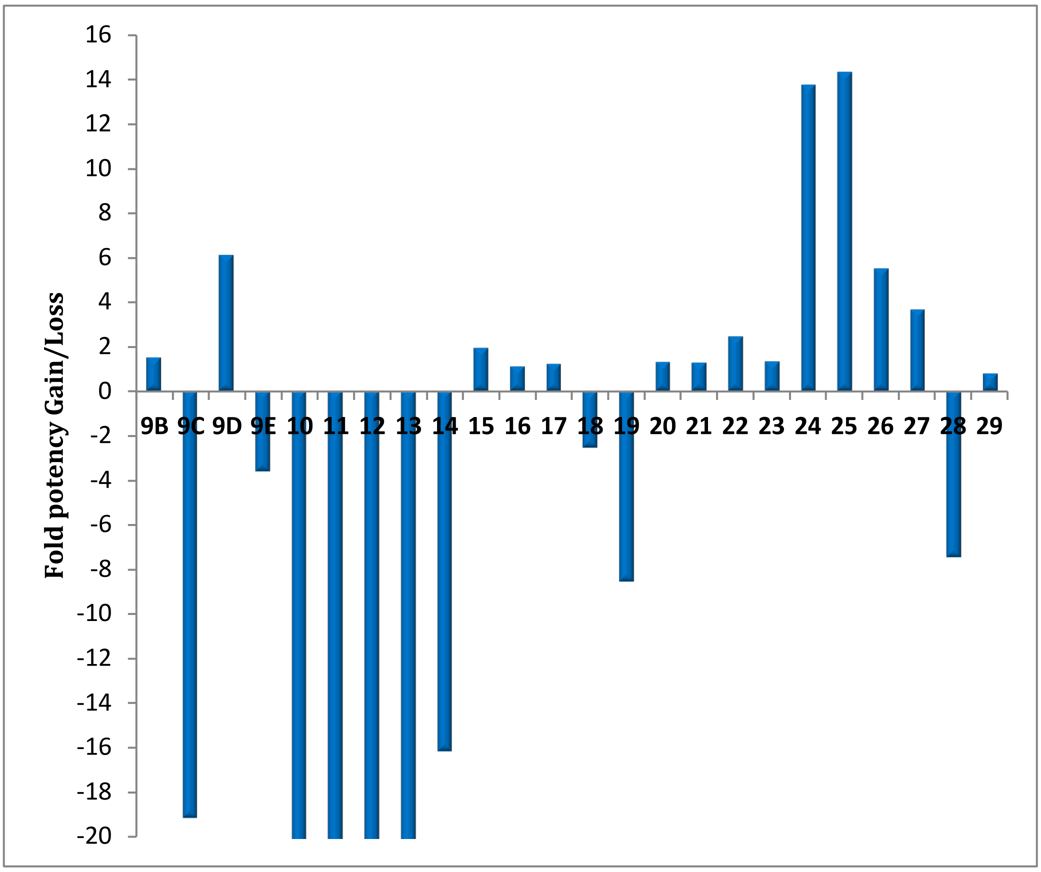

**Figure S4.** SPR binding assays of His$_6$-*hs*BMX with the covalent inhibitors **BMX-IN-1**, **JS9D**, **JS24–27** and the low affinity binders **JS9C**, **JS9E**, **JS10**, **JS12.** The sensorgrams show the response upon compound injection (t=0s), or upon washing (t=220s), on immobilized His6-hsBMX surfaces. Compound concentrations injected on already saturated surfaces were not included in the 1:1 Langmuir fitting model. X-axis: time (s); y-axis: response (arbitrary units). Inset plots [x-axis: compound concentration (M); y-axis: response (arbitrary units)] show the steady-state affinity ($K_D$ss) fit for the interactions wherein equilibrium was reached.

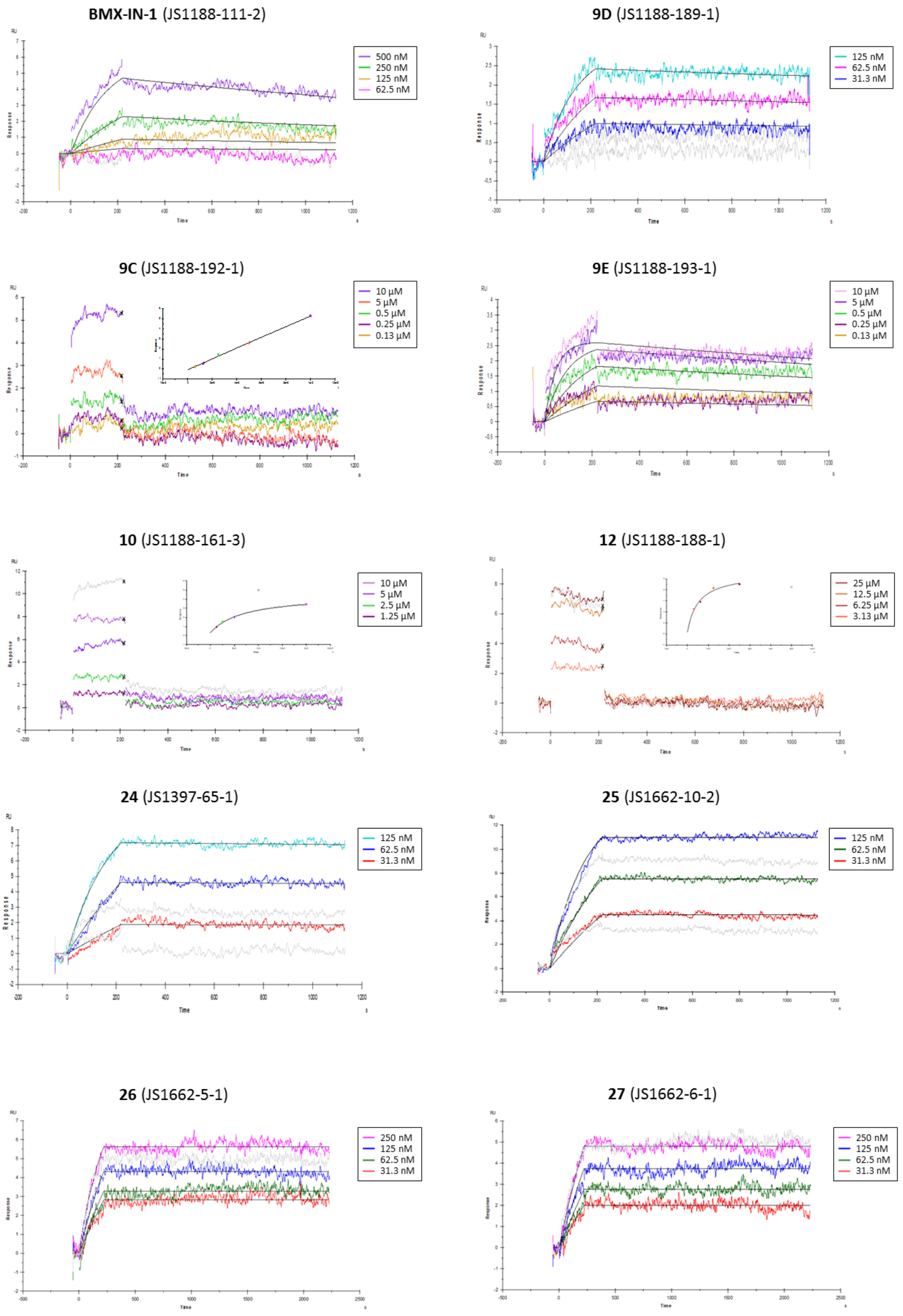

**Figure S5.** Co-crystal structure showing the binding mode of **JS24** to hBMX kinase catalytic domain. **a.** Hydrophobic interactions between the aromatic rings of **JS24** and the side chains of Tyr491, Ala443, Val431, and Leu543; **b.** Positioning of the sulfonamide aromatic ring pointing out of the ATP pocket.

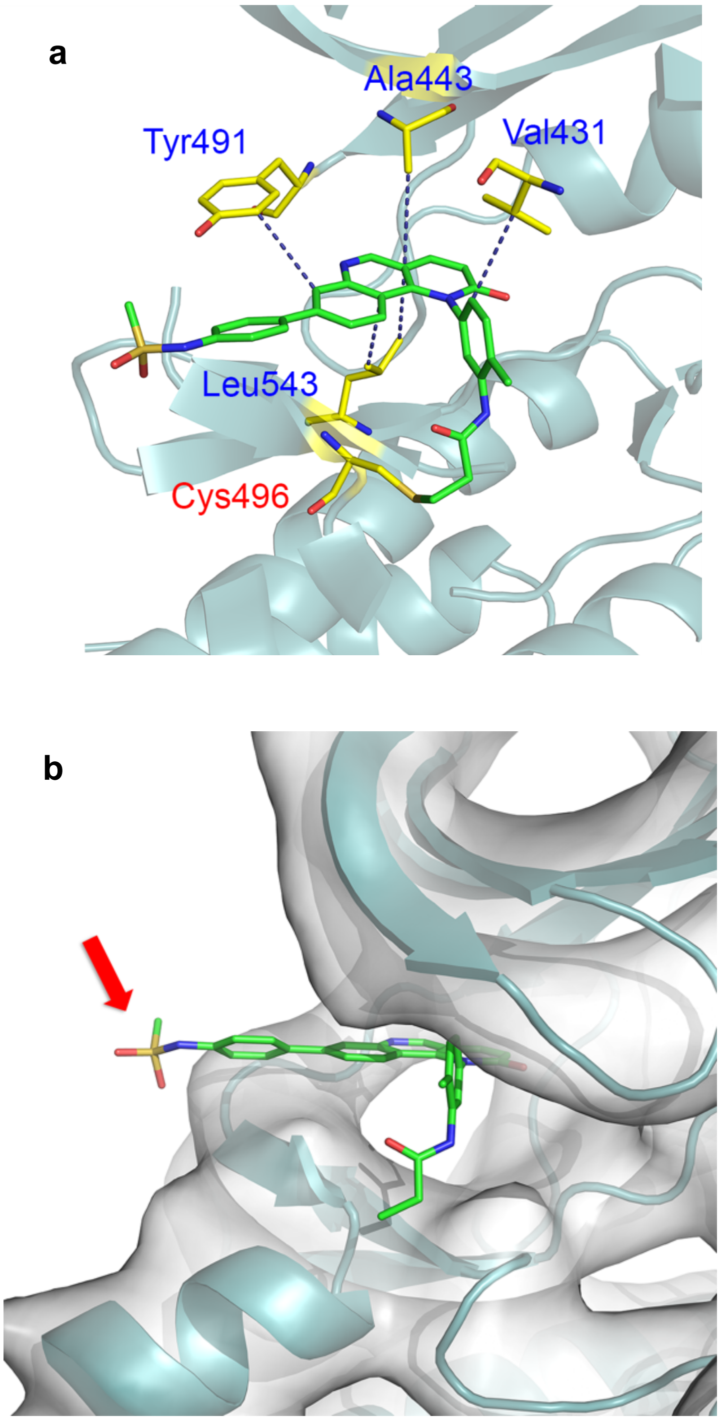

**Figure S6.** Distance evolution, derived from 0.5 μs MD simulations, between quinoline ring of **JS24** (a) and **JS27** (b) and methyl groups of Ala443, Val431, and Leu543 of the receptor. Distance evolution between the side chain of Tyr491 and the aromatic phenyl-sulfonamide ring (**JS24**) or piperazine ring (**JS27**) is also shown. In all cases, the centre of mass of the rings were considered in the calculations.

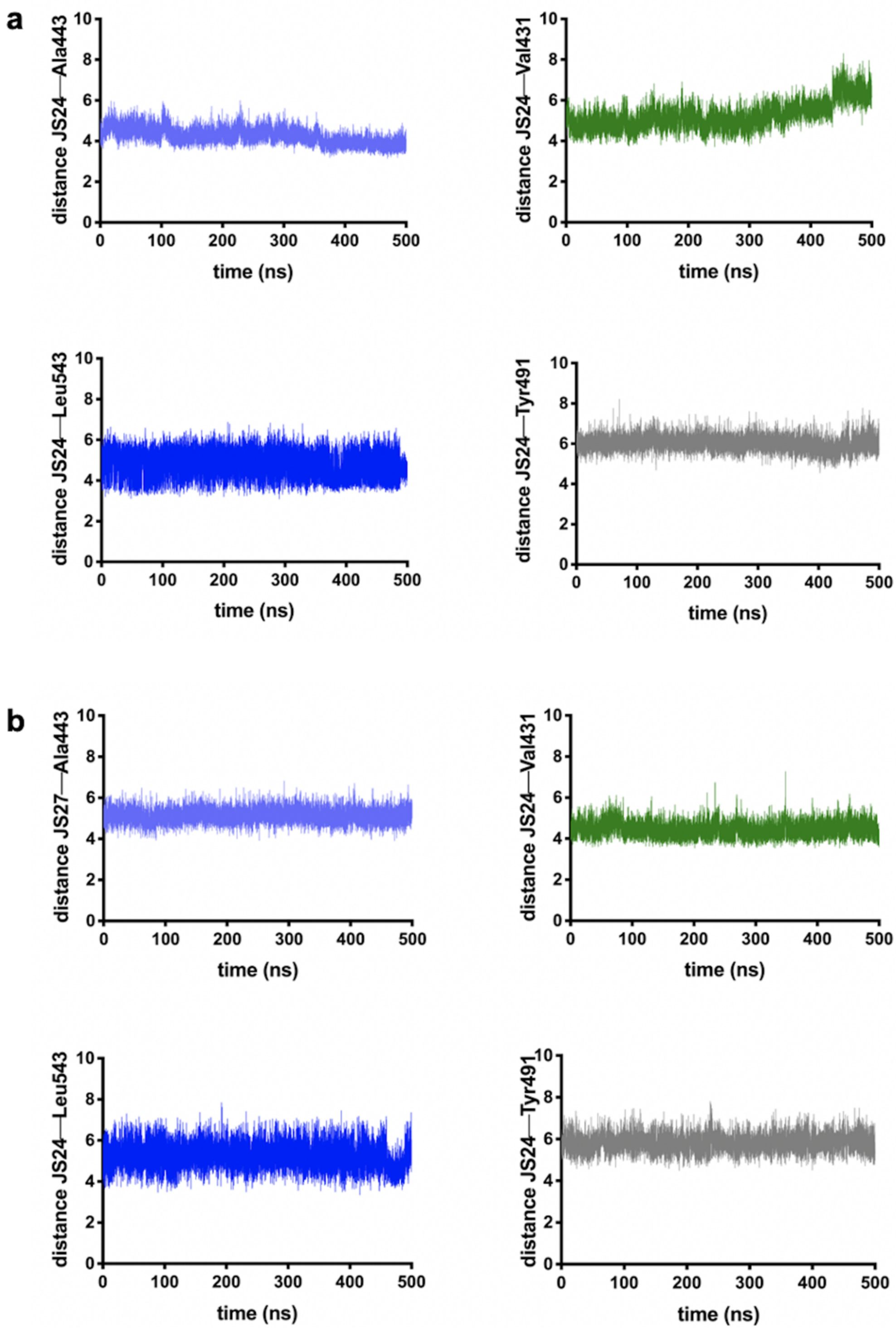

**Figure S7**. Effect of **BMX-IN-1** and analogues **JS24–27** on LNCaP prostate cancer cells. Flow Cytometry analysis of the compounds' effect on apoptosis.

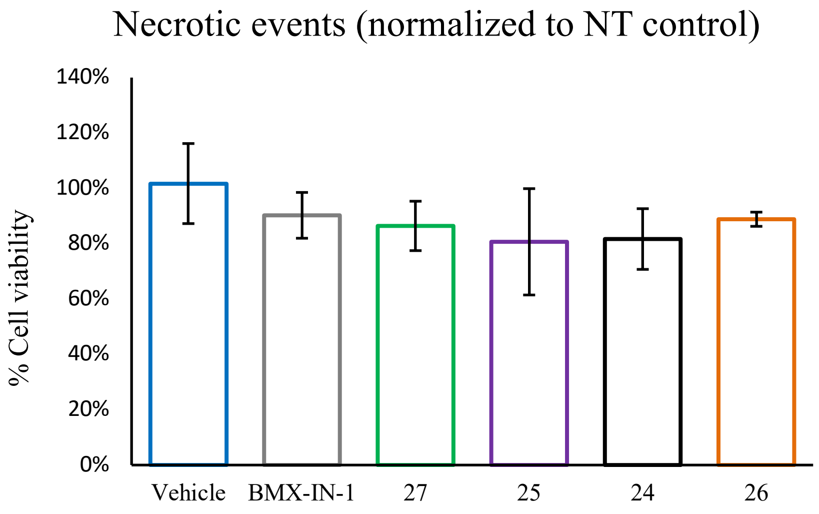


**Figure S8**. Anti-proliferative effects of **JS24-26**, AKT inhibitor **AKT1/2,** androgen receptor inhibitor **Flutamide** and PI3K inhibitor **LY294002** on LNCaP prostate cancer cells. Values are reported in % cell viability normalized to DMSO controls and are the mean±S.D. of one individual experiment performed in triplicate.

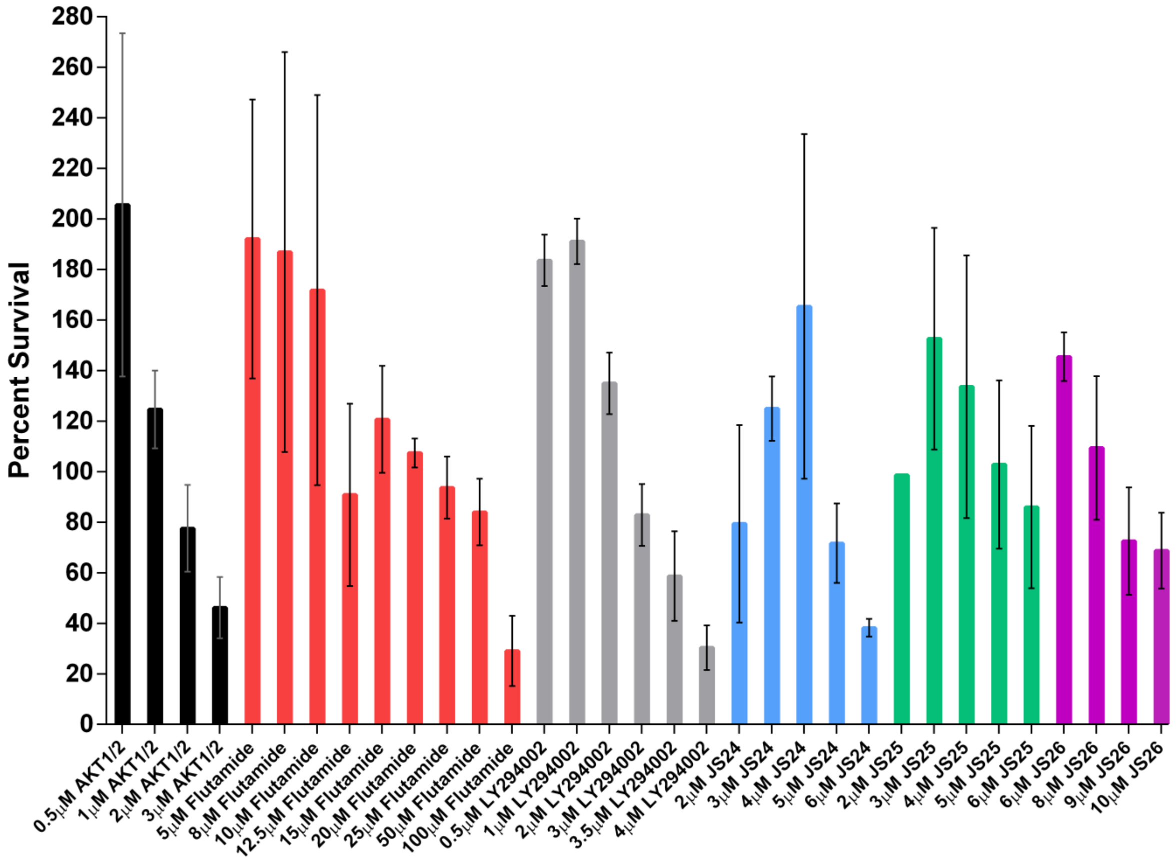

**Figure S9**. Synergistic anti-proliferative effects of **JS24-26** with AKT inhibitor **AKT1/2** (a), androgen receptor inhibitor **Flutamide** (b) and PI3K inhibitor **LY294002** (c) on the viability of LNCaP prostate cancer cells. Values are reported in % cell viability normalized to DMSO controls and are the mean±S.D. of three individual experiments performed in triplicate

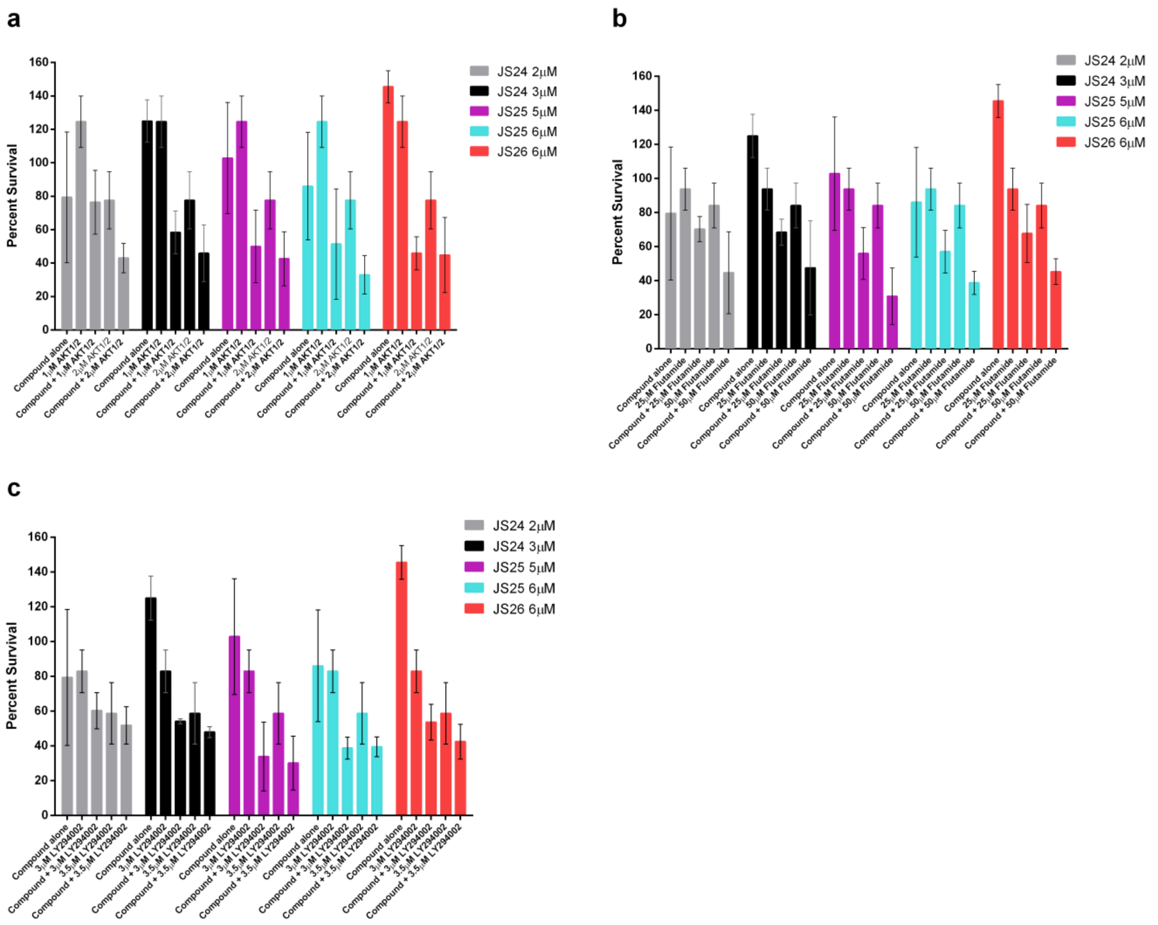

**Figure S10.** Kinase selectivity of compound **JS25** over 36 BMX-related kinases in the Eurofins DiscoveRx's KINOMEscan platform at the concentration of 1 μM.

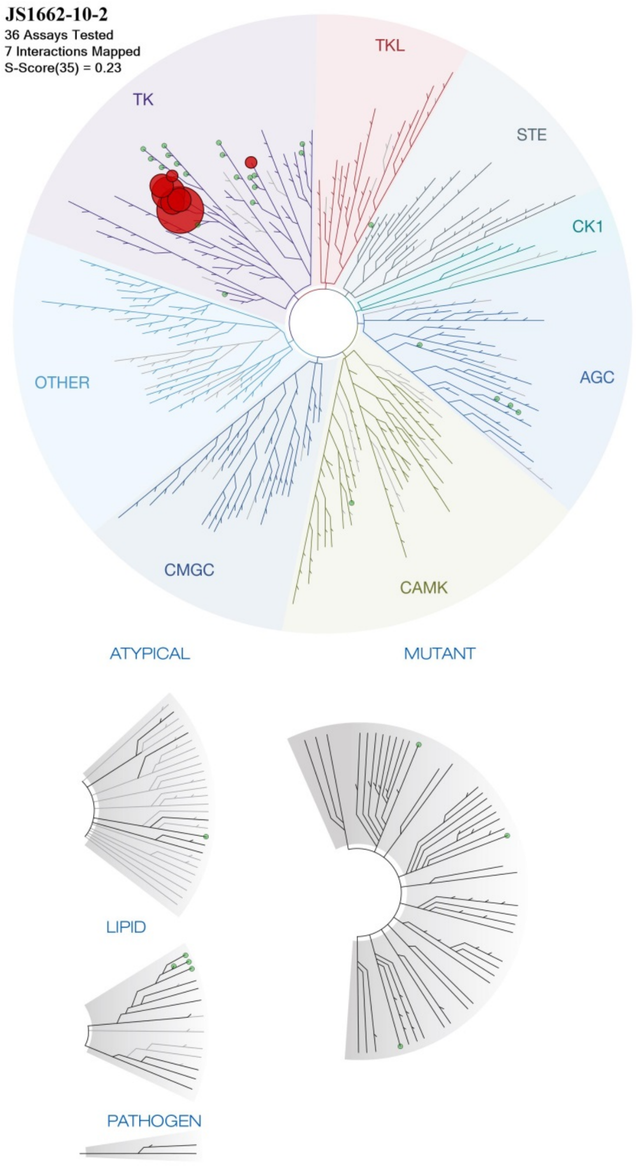

**Figure S11. BMX degradation after treatment with JS25 and BMX-IN-1 in PC3 cells.** (**a**) **JS25** (10 µM) and **BMX-IN-1** (10 µM) induce degradation of wild-type BMX in PC3 cells. Sampling was taken after 24 h and 72 h of incubation with **JS25**. (**b**) Raw data from BMX degradation assay. Protein band intensity normalized with α-Tubulin band intensity and quantified using ImageJ. Data was obtained from at least three independent measurements (*n*=3).

**a.**

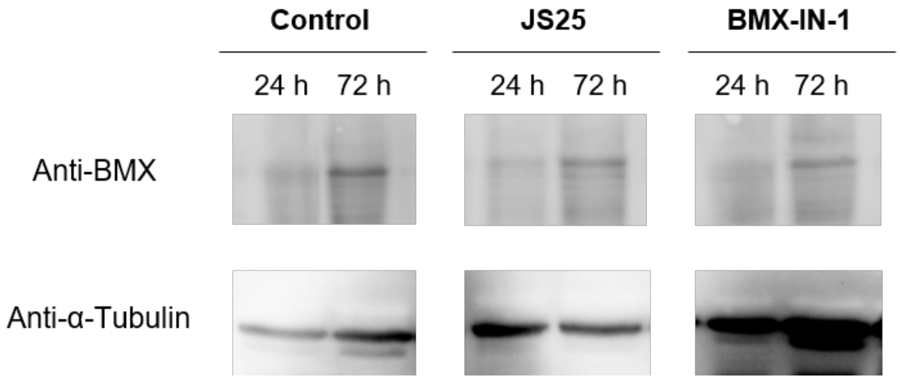


**b.**

| | 24 h (band intensity) | | | | | | 72 h (band intensity) | | | | | |
|---|---|---|---|---|---|---|---|---|---|---|---|---|
| | BMX | | | α-Tubulin | | | BMX | | | α-Tubulin | | |
| | **Control** | **JS25** | **BMX-IN-1** | **Control** | **JS25** | **BMX-IN-1** | **Control** | **JS25** | **BMX-IN-1** | **Control** | **JS25** | **BMX-IN-1** |
| Data 1 | 704 | 703 | 508 | 2789 | 8382 | 13575 | 14854 | 4159 | 4514 | 10293 | 6767 | 10638 |
| Data 2 | 767 | 486 | 806 | 2683 | 8330 | 13347 | 15728 | 4349 | 4840 | 12293 | 6546 | 9866 |
| Data 3 | 536 | 507 | 605 | 2611 | 8706 | 14523 | 15399 | 4809 | 4526 | 12585 | 6641 | 9965 |

**Scheme S1.** Synthetic route for the preparation of compounds **JS9a–e**, **JS10–13** and **JS14–23**.

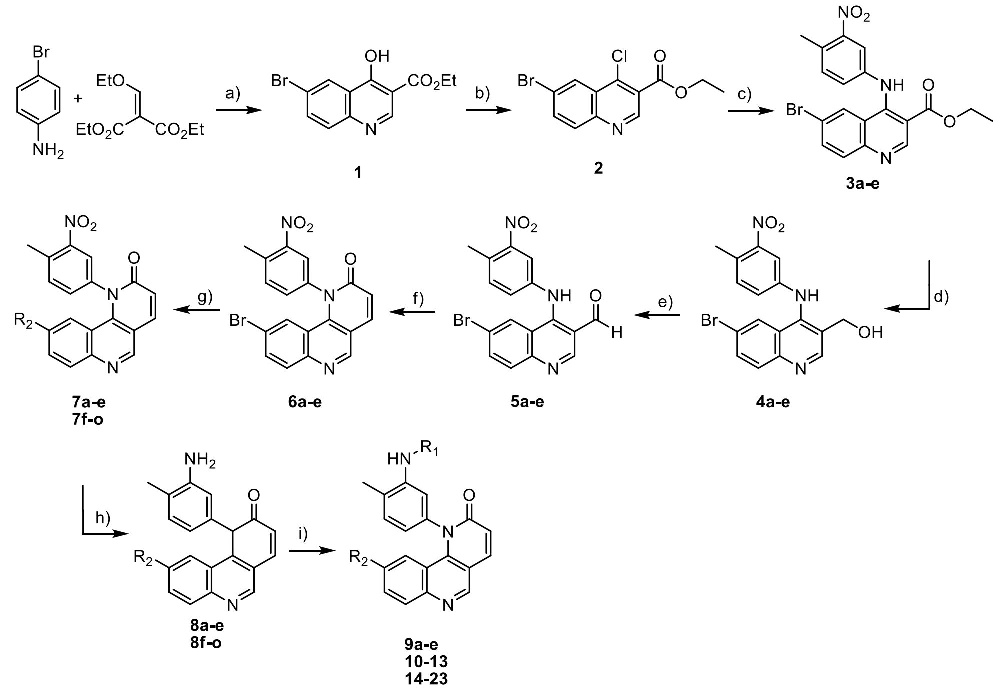


a) Reflux (neat), 24h, then $Ph_2O$, 5h at 240 ºC, 40%; b) $SOCl_2$, reflux, 5 h, 100%; c) aniline, dioxane, 90 ºC, 6 h, 90%; d) $NaBH_4$, EtOH, rt, overnight, 100%; e) Dess-Martin Periodinane, DCM, rt, 3 h, 75%; f) Triethylphosphonoacetate, $K_2CO_3$, EtOH, 100 ºC, overnight, 60%; g) boronic acid/ester, $PdCl_2(PPh_3)_2$, $Na_2CO_3$, dioxane, 90 ºC, overnight, 50% or amine, $Pd(OAc)_2$, R-BINAP, $Cs_2CO_3$, dioxane, 85 ºC, overnight, 80%; h) Fe, $NH_4Cl$, EtOH/$H_2O$, 80 ºC, 2 h, 100%; i) acyl chloride, DIPEA, THF, -10 ºC to rt 5 h, 25% or alkyl halide, $K_2CO_3$, DMF, rt, overnight, 35%.

**Scheme S2.** Synthetic route for the preparation of compounds **JS24–27**.

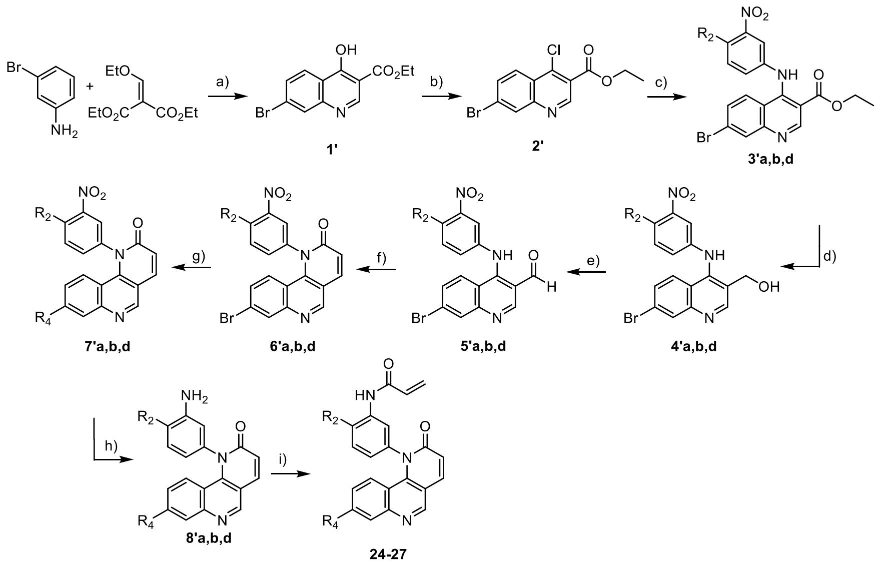

a) Reflux (neat), 24h, then $Ph_2O$, 5h at 240 ºC, 40%; b) $SOCl_2$, reflux, 5 h, 100%; c) aniline, dioxane, 90 ºC, overnight, 80%; d) $NaBH_4$, EtOH, rt, overnight, 90%; e) Dess-Martin Periodinane, DCM, rt, 3 h, 80%; f) Triethylphosphonoacetate, $K_2CO_3$, EtOH, 100 ºC, overnight, 70%; g) boronic acid/ester, $PdCl_2(PPh_3)_2$, $Na_2CO_3$, dioxane, 90 ºC, overnight, 20% or amine, $Pd(OAc)_2$, R-BINAP, $Cs_2CO_3$, dioxane, 85 ºC, overnight, 90%; h) Fe, $NH_4Cl$, $EtOH/H_2O$, 80 ºC, 2 h, 70%; i) acryloyl chloride, DIPEA, THF, -10 ºC to rt 5 h, 15%

**Scheme S3.** Synthetic route for the preparation of compound **JS28**.

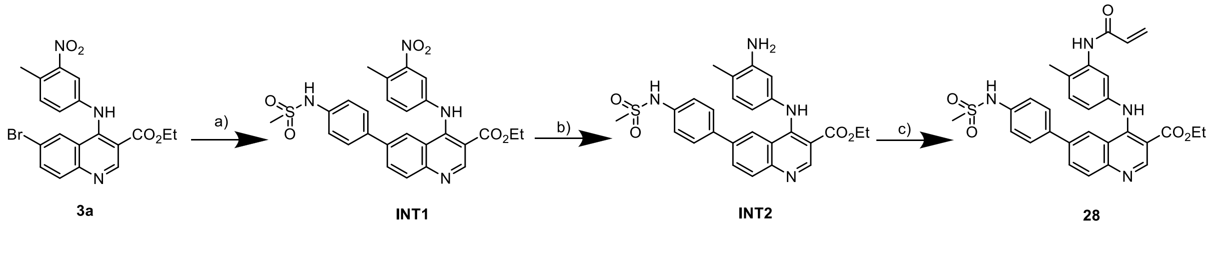

a) 4-(methanesulfonylamino) phenyl boronic acid pinacol ester, $PdCl_2(PPh_3)_2$, $Na_2CO_3$, dioxane, 90 ºC, overnight, 73%; b) Fe, $NH_4Cl$, $EtOH/H_2O$, 80 ºC, 2 h, 88%; c) acryloyl chloride, DIPEA, THF, -10 ºC to rt, 5 h, 10%.

**Scheme S4.** Synthetic route for the preparation of compound **JS29**.

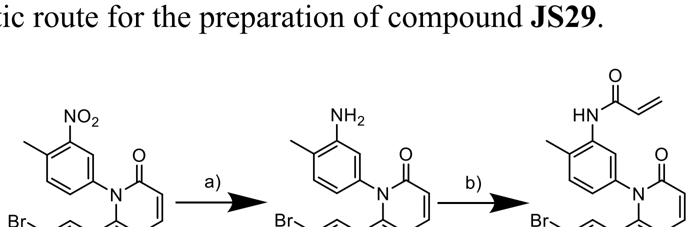

a) $SnCl_2$, EtOAc, 85 ºC, 2 h, 68%; b) acyl chloride, DIPEA, THF, -10 ºC to rt, 5 h, 95%.

**Table S1.** *In silico* cLogP and LogS calculation and *in vitro* artificial membrane permeability (PAMPA) and colloidal aggregation (DLS) determination.

| Compound | cLogP | LogS | PAMPA/Pe | PAMPA/%R | DLS |
|---|---|---|---|---|---|
| **BMX-IN-1** | 3.94 | -5.98 | 8.9±0.8 | 55±8 | 10 µM |
| **JS9B** | 3.88 | -5.98 | 7.1±2.3 | 35±7 | < 0.1 µM |
| **JS9C** | 3.87 | -5.98 | 11±1 | 51±6 | 1 µM |
| **JS9D** | 3.59 | -5.64 | 11±1 | 48±3 | 1 µM |
| **JS9E** | 3.60 | -5.76 | 40±5 | 55±8 | 1 µM |
| **JS10** | 4.19 | -6.21 | 28±4 | 33±4 | 0.1 µM |
| **JS11** | 4.51 | -6.77 | 31±4 | 54±9 | 0.1 µM |
| **JS12** | 4.06 | -6.27 | 45±3 | 39±2 | 0.1 µM |
| **JS13** | 4.22 | -6.43 | 45±9 | 30±16 | 0.1 µM |
| **JS14** | 4.92 | -7.04 | Und | --- | 0.1 µM |
| **JS15** | 3.92 | -5.98 | 30±18 | 17±24 | 1 µM |
| **JS16** | 4.43 | -6.23 | Und | --- | 10 µM |
| **JS17** | 3.98 | -5.33 | 24±1 | 10±3 | 10 µM |
| **JS18** | 3.83 | -5.69 | 24±2 | 18±5 | < 0.1 µM |
| **JS19** | 4.97 | -6.25 | Und | --- | < 0.1 µM |
| **JS20** | 4.00 | -5.52 | 30±2 | 18±1 | 10 µM |
| **JS21** | 3.22 | -4.61 | 22±1 | 19±3 | 10 µM |
| **JS22** | 2.56 | -4.71 | 18±1 | 19±6 | 10 µM |
| **JS23** | 3.72 | -5.43 | 2.2±0.2 | 39±5 | 10 µM |
| **JS24** | 3.96 | -5.98 | 6.8±0.4 | 13±1 | 1 µM |
| **JS25** | 4.02 | -5.98 | 3.8±1.6 | 10±18 | 0.1 µM |
| **JS26** | 3.59 | -5.64 | 19±13 | 8±14 | 10 µM |
| **JS27** | 2.32 | -4.36 | 12±2 | 42±14 | 10 µM |
| **JS28** | 4.33 | -7.01 | Und | --- | < 0.1 µM |
| **JS29** | 4.03 | -5.32 | 32±4 | 1±1 | 10 µM |

cLogP and LogS were calculated using SwissADME software. cLog P is a consensus value obtained as the arithmetic mean of five freely available predictive models (XLOGP3, WLOGP, MLOGP, SILICOS-IT and iLOGP) and LogS is the arithmetic mean of two topological methods (ESOL model[1] and Ali *et al.*[2]) PAMPA: $P_e$ is effective permeability (x$10^{-6}$ cm/sec) measured directly from assay at pH 6.8 and %R is membrane retention. All values are reported as the average of quadruplicates. Und label refers to compounds with extremely low solubility for which UV limits were below the detection limits therefore considered undetected. Compounds were labelled as high permeability (green), medium permeability (orange) or low permeability (red). DLS is measured at 10 µM, 1µM and 100 nM. The maximum soluble concentration – at which no aggregates are observed - is indicated and the colour code indicates if the compound forms aggregates at the $IC_{50}$ concentration (green – no aggregation at $IC_{50}$ concentration; red - aggregation at $IC_{50}$ concentration.

**Table S2.** Melting temperature (Tm) shift calculated with a DSF assay.

| Compound | Average Tm | apo-BMX Tm | ΔTm (ºC) |
|---|---|---|---|
| **BMX-IN-1** | 60.17±0.32 | 52.13±0.11 | 8.04±0.32 |
| **JS9B** | 59.68±0.11 | 51.93±0.22 | 7.75±0.11 |
| **JS9C** | 54.12±0.06 | 51.92±0.31 | 2.20±0.06 |
| **JS9D** | 59.66±0.01 | 51.92±0.31 | 7.74±0.01 |
| **JS9E** | 57.97±0.01 | 51.92±0.31 | 6.05±0.01 |
| **JS10** | 59.86±0.06 | 51.92±0.31 | 6.82±0.02 |
| **JS11** | 52.26±0.11 | 51.92±0.31 | 0.34±0.11 |
| **JS12** | 52.05±0.06 | 51.92±0.31 | 0.13±0.06 |
| **JS13** | 52.44±0.06 | 51.92±0.31 | 0.52±0.06 |
| **JS14** | 59.53±0.11 | 51.92±0.31 | 7.61±0.11 |
| **JS15** | 60.72±0.28 | 51.93±0.22 | 8.79±0.28 |
| **JS16** | 60.02±0.06 | 51.93±0.22 | 8.09±0.06 |
| **JS17** | 59.55±0.01 | 51.93±0.22 | 7.62±0.01 |
| **JS18** | 59.42±0.11 | 51.93±0.22 | 7.49±0.11 |
| **JS19** | 58.64±0.13 | 51.82±0.19 | 6.82±0.13 |
| **JS20** | 59.29±0.11 | 51.93±0.22 | 7.36±0.11 |
| **JS21** | 60.50±0.06 | 51.93±0.22 | 8.57±0.06 |
| **JS22** | 60.72±0.21 | 51.93±0.22 | 8.79±0.21 |
| **JS23** | 60.59±0.11 | 51.93±0.22 | 8.66±0.11 |
| **JS24** | 63.57±0.01 | 52.23±0.06 | 11.34±0.01 |
| **JS25** | 61.43±0.48 | 52.13±0.11 | 9.30±0.48 |
| **JS26** | 61.47±0.21 | 52.13±0.11 | 9.34±0.21 |
| **JS27** | 62.94±0.06 | 52.13±0.11 | 10.81±0.06 |

**Table S3.** Kinetic constants calculated from Surface Plasmon Resonance.

| Compound | $K_{Dss}$/M | $k_{on}$/$M^{-1}s^{-1}$ | $k_{off}$/$s^{-1}$ | $K_D$/M |
|---|---|---|---|---|
| **BMX-IN-1** | Nd | $7.4 \times 10^{3}$ | $5.10 \times 10^{-4}$ | $6.9 \times 10^{-8}$ |
| **JS9C** | $> 1 \times 10^{-6}$ | Nd | Nd | Nd |
| **JS9D** | Nd | $1.4 \times 10^{4}$ | $< 1 \times 10^{-4}$ | $< 1 \times 10^{-8}$ |
| **JS9E** | Nd | $1.9 \times 10^{3}$ | $2.4 \times 10^{-4}$ | $1.3 \times 10^{-7}$ |
| **JS10** | $3.9 \times 10^{-6}$ | Nd | Nd | Nd* |
| **JS12** | $7.4 \times 10^{-6}$ | Nd | Nd | Nd* |
| **JS24** | Nd | $1.4 \times 10^{5}$ | $< 1 \times 10^{-4}$ | Nd* |
| **JS25** | Nd | $5.4 \times 10^{4}$ | $< 1 \times 10^{-4}$ | Nd* |
| **JS26** | Nd | $7.2 \times 10^{4}$ | $< 1 \times 10^{-4}$ | Nd* |
| **JS27** | Nd | $9.9 \times 10^{4}$ | $< 1 \times 10^{-4}$ | Nd* |

Nd – not determined; *Immeasurable $K_D$ due to very prolonged off-rates (outside instrument specifications).

**Table S4.** Data processing statistics for the crystallographic structure of BMX in complex with inhibitor **JS24**.

| | |
|---|---|
| Wavelength (Å) | 0.9677 |
| Temperature (K) | 100 |
| Scan type | ω |
| Total no. of frames | 900 |
| Width (°) | 0.25 |
| Total angular range (°) | 225 |
| Exposure time per image (s) | 0.005 |
| Space group | $P\,2_1$ |
| Unit cell parameters | |
| *a*, *b*, *c* (Å) | 66.70, 63.29, 74.96 |
| β (°) | 104.55 |
| CCP4 TRUNCATE processing | |
| Resolution (°) [a] | 63.3-2.19 (2.22-2.19) |
| Nr. of observations | 133203 (4732) |
| Unique reflections | 30574 (1441) |
| Multiplicity | 4.4 (3.3) |
| Completeness (%) | 97.1 (91.7) |
| R-merge (%) [b] | 23.6 (165) |

| | |
|---|---|
| R-p.i.m. (%) [c] | 12.9 (108) |
| <I/σ (I)> | 4.8 (0.7) |
| CC½ | 0.990 (0.394) |
| autoPROC STARANISO processing | |
| Resolution limits of ellipsoid fitted to resolution cut-off surface (Å): | |
| | 1.98, 2.80, 2.25 |
| Resolution, spherical limits (°) [a] | 72.6-1.98 (2.24-1.98) |
| Nr. of observations | 99785 (3815) |
| Unique reflections | 23531 (1161) |
| Multiplicity | 4.2 (3.3) |
| Completeness, spherical (%) | 56.0 (9.1) |
| Completeness, ellipsoidal (%) | 87.6 (51.7) |
| R-merge (%) [b] | 18.1 (76.0) |
| R-p.i.m. (%) [c] | 10.1 (48.9) |
| <I/σ (I)> | 6.1 (1.5) |
| CC½ | 0.991 (0.586) |
| Wilson B (Å²) | 23.6 |
| Z [d] | 2 |
| Estimated $V_M$ [e] | 2.46 |
| Estimated solvent content (%) [e] | 50.1 |

[a]Values in parentheses refer to the highest resolution shell; [b] R-merge=merging R-factor, ($\Sigma_{hkl}$ $\Sigma_i$ I$I_i$ (hkl) - </(hkl)>I) / ($\Sigma_{hkl}$ $\Sigma_i$ $I$(hkl) x 100 % ; [c] R-p.i.m. = precision-independent R-factor, $\Sigma_{hkl}$ $[1/(N-1)]^{1/2}$ $\Sigma_i$ I$I_i$ (hkl) - <$I$(hkl) >I / ($\Sigma_{hkl}$ $\Sigma_i$ $I_i$ (hkl) x 100 %. For each unique Bragg reflection with indices (hkl), $I_i$ is the i-th observation of its intensity and N its multiplicity; [d] Nr. of molecules in the asymmetric unit; [e] According to Matthews coefficient.

**Table S5.** Refinement statistics for the crystallographic structure of BMX in complex with inhibitor **JS24**.

| | |
|---|---|
| Resolution limits (Å) [a] | 72.6 – 2.00 (2.09 – 2.00) |
| $R_{work}$ [b] | 23.2 (32.9) |
| $R_{free}$ [c] | 25.9 (44.6) |
| ML coordinate error estimate (Å) [d] | 0.26 |
| *Model composition* | |
| Non-hydrogen protein atoms | 4308 |
| Non-hydrogen ligand atoms | 76 |
| Solvent molecules | 126 |
| *Mean B values (Å$^2$)* [e] | |
| Protein main-chain | 32.4 |
| Protein side-chain | 35.5 |
| Ligands | 25.4 |
| Solvent | 23.2 |
| *Model r.m.s. deviations from ideality* | |
| Bond lengths (Å) | 0.002 |
| Bond angles (°) | 0.603 |
| Chiral centres (Å$^3$) | 0.041 |
| Planar groups (Å) | 0.003 |
| *Model validation* [i] | |
| % Ramachandran outliers | 0.0 |
| % Ramachandran favored | 98.3 |
| % Rotamer outliers | 0.63 |
| $C^{\beta}$ outliers | 0 |
| Clash score | 2.7 |

[a]Values in parentheses refer to the highest resolution shell; [b] $R_{work} = (\Sigma_{hkl} ||F_{obs}(hkl)| - |F_{calc}(hkl)||) / (\Sigma_{hkl} |F_{obs}(hkl)|) \times 100$ %; [c] $R_{free}$ is calculated as above from a random sample containing 5% of the total number of independent reflections measured; [d] Maximum-likelihood estimate by PHENIX; [e] Calculated from isotropic or equivalent isotropic B-values; [i] Calculated with MolProbity[88].

## 2. SYNTHESIS

**6-bromo-4-hydroxyquinoline-3-carboxylate (1)**

Diethyl 2-(ethoxymethylene) malonate (11.7 mL; 58.13 mmol) and 4-bromoaniline (10 g; 58.13 mmol) were heated to 145 ºC for 23 h. The solvent was then evaporated affording an off-white solid. $Ph_2O$ (25 ml) was added and the reaction heated to 245 ºC. After 6 h, no more intermediate was detected by TLC (EtOAc:Hexane 20:80). Upon cooling to rt, a precipitate was formed, and hexane was added to induce more precipitation. The precipitate was filtered, washed with EtOAc and dried in vacuum to afford the title compound as an off-white solid (6.9 g; 40% yield). $^1H$ NMR (300 MHz, $d^6$-DMSO): δ 8.60 (s, 1H), 8.22 (d, $J$ = 2.4 Hz, 1H), 7.82 (dd, $J$ = 8.7, 2.4 Hz, 1H), 7.58 (d, $J$ = 8.7 Hz, 1H), 4.20 (q, $J$ = 7.1 Hz, 2H), 1.27 (t, $J$ = 7.1 Hz, 3H).

**Ethyl 6-bromo-4-chloro-3-quinolinecarboxylate (2)**

6-bromo-4-hydroxy-3-quinolinecarboxylate (13.4 g; 45.25 mmol) was suspended in $SOCl_2$ (130 mL; 1.792 mol) and the mixture heated to 80 ºC. After 5 h, a clear yellow solution was obtained. The solvent was evaporated and the solid co-evaporated with DCM (5x) to remove residual HCl. It was dried in vacuum to afford the title compound as a light-yellow solid (14.4 g; 100% yield). $^1H$ NMR (300 MHz, $CDCl_3$): δ 9.37 (s, 1H), 8.71 (d, $J$ = 2.0 Hz, 1H), 8.56 (d, $J$ = 9.0 Hz, 1H), 8.12 (dd, $J$ = 9.0, 2.0 Hz, 1H), 4.53 (q, $J$ = 7.1 Hz, 2H), 1.47 (t, $J$ = 7.1 Hz, 3H).

*General Procedure A: Nucleophilic Aromatic Substitution*

**Ethyl 6-bromo-4-((4-methyl-3-nitrophenyl)amino)quinoline-3-carboxylate (3a)**

Ethyl 6-bromo-4-chloro-3-quinolinecarboxylate **2** (800 mg; 2.543 mmol**)** and 4-methyl-5-nitroaniline (387 mg; 2.543 mmol) were mixed in dioxane (15 mL) and heated to 90 ºC. After

7 h, TLC analysis (50% EtOAc/Hexane) no longer detected starting materials. The yellow suspension was cooled to rt, diluted with $H_2O$ and NaOH (1M) added until pH=8 was reached. EtOAc was added and the phases were separated. The aqueous phase was further extracted with EtOAc (2x) and the combined organics were washed with brine and dried over $MgSO_4$. After filtration, the solvent was evaporated to afford the title compound as a bright-yellow solid (980 mg; 90% yield). $^1$H NMR (300 MHz, $CDCl_3$): δ 10.38 (s, 1H), 9.29 (s, 1H), 7.90 (d, *J* = 9.4 Hz, 1H), 7.74 (dq, *J* = 4.3, 2.2 Hz, 2H), 7.65 (d, *J* = 2.5 Hz, 1H), 7.25 (d, *J* = 8.3 Hz, 1H), 7.08 (dd, *J* = 8.3, 2.5 Hz, 1H), 4.45 (q, *J* = 7.1 Hz, 2H), 2.58 (s, 3H), 1.46 (t, *J* = 7.1 Hz, 3H).

**Ethyl 6-bromo-4-((3-methyl-5-nitrophenyl)amino)quinoline-3-carboxylate (3b)**

Prepared using General Procedure A, reacting intermediate **2** with with 3-methyl-5-nitroaniline. Compound **3b** was isolated as a yellow solid (730 mg; 89% yield). $^1$H NMR (300 MHz, $CDCl_3$): δ 10.35 (s, 1H), 9.30 (s, 1H), 7.92 (d, J = 8.8 Hz, 1H), 7.79-7.73 (m, 3H), 7.61 (s, 1H), 7.11 (s, 1H), 4.45 (q, J = 7.1 Hz, 2H), 2.39 (s, 3H), 1.45 (t, J = 7.1 Hz, 3H). $^{13}$C NMR (75.5 MHz, $CDCl_3$): δ 167.9, 151.5, 150.2, 149.6, 149.0, 143.5, 141.2, 135.1, 132.1, 128.4, 127.5, 120.8, 119.7, 119.2, 113.2, 109.1, 61.9, 14.4. HRMS (ESI): *m/z* $[M + H]^+$ calc. for $C_{19}H_{17}BrN_3O_4$: 430.0397; found: 430.0401.

**Ethyl 6-bromo-4-((2-methyl-5-nitrophenyl)amino)quinoline-3-carboxylate (3c)**

Prepared using General Procedure A, reacting intermediate **2** with 2-methyl-5-nitroaniline. The reaction required heating at 90 ºC for 23 h followed by 4 h at 110 ºC. Compound **3c** was isolated as a yellow solid (730 mg, 89% yield). $^1$H NMR (300 MHz, $d^6$-DMSO): δ 9.29 (s, 1H), 8.87 (s, 1H), 8.52 – 8.47 (m, 1H), 7.98 – 7.90 (m, 3H), 7.66 (d, J = 2.4 Hz, 1H), 7.60 (d, J = 8.5 Hz, 1H), 3.85 (q, J = 7.1 Hz, 2H), 2.44 (s, 3H), 1.04 (t, J = 7.1 Hz, 3H). HRMS (ESI): *m/z* $[M + H]^+$ calc. for $C_{19}H_{17}BrN_3O_4$: 430.0397; found: 430.0400. $^{13}$C NMR could not be acquired

because the compound is not sufficiently soluble in $d^6$-DMSO, $d^6$-acetone, $d^3$-acetonitrile or $d^4$-methanol.

**Ethyl 6-bromo-4-((3-nitrophenyl)amino)quinoline-3-carboxylate (3d)**

Prepared using General Procedure A and reacting intermediate **2** with with 3-nitroaniline for 8 h. Compound **3d** was isolated as an orange solid (640 mg: 81% yield). $^1$H NMR (300 MHz, $CDCl_3$): δ 10.41 (s, 1H), 9.32 (s, 1H), 8.01 – 7.91 (m, 2H), 7.86 (t, J = 2.2 Hz, 1H), 7.79 – 7.70 (m, 2H), 7.51 – 7.42 (m, 1H), 7.28 – 7.23 (m, 1H), 4.47 (q, J = 7.1 Hz, 2H), 1.47 (t, J = 7.2 Hz, 3H). $^{13}$C NMR (75.5 MHz, $CDCl_3$): δ 167.9, 151.5, 150.1, 149.5, 149.0, 143.7, 135.1, 132.1, 130.0, 128.3, 126.6, 120.8, 119.4, 119.0, 115.9, 109.4, 62.0, 14.3. HRMS (ESI): *m/z* $[M + H]^+$ calc. for $C_{18}H_{15}BrN_3O_4$: 416.0240; found: 416.0245.

**Ethyl 6-bromo-4-((4-methoxy-3-nitrophenyl)amino)quinoline-3-carboxylate (3e)**

Prepared using the General Procedure A and reacting intermediate **2** with 4-methoxy-3-nitroaniline. Compound **3e** was isolated as an orange solid (780 mg; 92% yield). $^1$H NMR (300 MHz, $CDCl_3$): δ 10.43 (s, 1H), 9.26 (s, 1H), 7.88 (d, J = 9.3 Hz, 1H), 7.73 – 7.70 (m, 2H), 7.59 (d, J = 2.7 Hz, 1H), 7.22 – 7.18 (m, 1H), 7.04 (d, J = 9.0 Hz, 1H), 4.45 (q, J = 7.1 Hz, 2H), 3.98 (s, 3H), 1.46 (t, J = 7.1 Hz, 3H). $^{13}$C NMR (75.5 MHz, $CDCl_3$): δ 168.2, 151.7, 151.1, 150.4, 149.7, 139.7, 135.2, 134.9, 132.1, 128.4, 128.1, 120.4, 119.7, 118.9, 114.6, 107.8, 61.8, 57.1, 14.4. HRMS (ESI): *m/z* $[M + H]^+$ calc. for $C_{19}H_{17}BrN_3O_5$: 446.0346; found: 446.0347.

**Ethyl 7-bromo-4-(4-methyl-3-nitrophenylamino)quinoline-3-carboxylate (3a')**

Prepared using General Procedure A and reacting intermediate **2'** with 4-methyl-5-nitroaniline for 3 h. Compound **3f** was isolated as a yellow solid (1.85 g; 85% yield). $^1$H NMR (300 MHz, $CDCl_3$): δ 10.54 (s, 1H), 9.31 (s, 1H), 8.26 (d, *J* = 2.0 Hz, 1H), 7.69 (d, *J* = 2.4 Hz,

1H), 7.48 (d, $J$ = 9.1 Hz, 1H), 7.34 (dd, $J$ = 9.1, 2.0 Hz, 1H), 7.28 (m, 1H), 7.13 (dd, $J$ = 8.3, 2.4 Hz, 1H), 4.48 (q, $J$ = 7.1 Hz, 2H), 2.60 (s, 3H), 1.49 (t, $J$ = 7.1 Hz, 3H). $^{13}$C NMR (75.5 MHz, $CDCl_3$): δ 168.0, 152.1, 151.8, 151.3, 149.6, 141.5, 133.7, 132.4, 129.6, 128.8, 127.3, 126.4, 126.2, 117.9, 117.7, 108.0, 61.9, 20.1, 14.4. HRMS (ESI): *m/z* $[M + H]^+$ calc. for $C_{19}H_{17}BrN_3O_4$: 430.0397; found: 430.0393.

**Ethyl 7-bromo-4-(3-methyl-5-nitrophenylamino)quinoline-3-carboxylate (3b')**

Prepared using General Procedure A and reacting intermediate **2'** with with 3-methyl-5-nitroaniline for 17 h.The crude was washed with cold EtOAc to remove unreacted aniline. Compound **3b'** was isolated as an orange solid (740 mg: 58% yield). $^1$H NMR (300 MHz, $CDCl_3$) δ 10.41 (s, 1H), 9.31 (s, 1H), 8.35 – 8.08 (m, 1H), 7.79 (s, 1H), 7.63 (s, 1H), 7.46 (d, $J$ = 9.1 Hz, 1H), 7.37 – 7.28 (m, 1H), 7.10 (s, 1H), 4.46 (q, $J$ = 7.0 Hz, 2H), 2.39 (s, 3H), 1.46 (t, $J$ = 7.1 Hz, 3H). HRMS (ESI): *m/z* $[M + H]^+$ calc. for $C_{19}H_{17}BrN_3O_4$: 430.0397; found: 430.0390.

**Ethyl 7-bromo-4-(3-nitrophenylamino)quinoline-3-carboxylate (3d')**

Prepared using General Procedure A and reacting intermediate **2'** with with 3-nitroaniline for 17h. Compound **3d'** was isolated as an orange solid (2.52 g: 95% yield). $^1$H NMR (300 MHz, $CDCl_3$): δ 10.41 (s, 1H), 9.31 (s, 1H), 8.24 (brs, 1H), 7.79 (s, 1H), 7.63 (s, 1H), 7.46 (d, $J$ = 9.1 Hz, 1H), 7.34 – 7.28 (m, 1H), 7.10 (s, 1H), 4.46 (q, $J$ = 7.1 Hz, 2H), 2.39 (s, 3H), 1.46 (t, $J$ = 7.1 Hz, 3H). $^{13}$C NMR (75.5 MHz, $CDCl_3$): δ 168.0, 152.3, 151.7, 151.2, 149.1, 144.0, 132.9, 130.3, 129.0, 127.2, 126.9, 126.3, 119.0, 118.2, 116.0, 108.8, 62.0, 14.4. HRMS (ESI): *m/z* $[M + H]^+$ calc. for $C_{18}H_{15}BrN_3O_4$: 416.0240; found: 416.0244.

*General Procedure B: Ester to Alcohol Reduction*

**(6-bromo-4-((4-methyl-3-nitrophenyl)amino)quinolin-3-yl)methanol (4a)**

Sodium borohydride (5.44 g; 143.99 mmol; 15 equiv.) was added portionwise to a stirred solution of **3a** (4.13 g; 9.599 mmol) in EtOH (35 mL) at 0 ºC. After 15h, TLC analysis (50% EtOAc/Hexane) showed disappearance of starting material. The orange solution was cooled in an ice-bath and quenched with $NH_4Cl$ aq.. The mixture was partitioned between $H_2O$ and EtOAc. The phases were separated, and the aqueous phase further extracted with EtOAc (2x). The combined organics were washed with brine, dried over $MgSO_4$ and evaporated to dryness to afford the title compound as an orange solid (3.73 g; 100% yield).

**(6-bromo-4-((3-methyl-5-nitrophenyl)amino)quinolin-3-yl)methanol (4b)**

Prepared using the procedure described for **4a**. Compound **4b** was obtained as an orange solid (630 mg; 100%). $^1H$ NMR (300 MHz, $d^6$-DMSO): δ 9.07 (s, 1H), 9.00 (s, 1H), 8.24 (s, 1H), 8.00 (d, J = 8.9 Hz, 1H), 7.87 (d, J = 8.9 Hz, 1H), 7.48 (s, 1H), 7.28 (s, 1H), 6.85 (s, 1H), 5.47 (t, J = 5.4 Hz, 1H), 4.48, (d, J = 5.4 Hz, 2H), 2.31 (s, 3H). $^{13}C$ NMR (75.5 MHz, $d^6$-DMSO): δ 152.2, 148.6, 146.9, 146.1, 140.6, 139.9, 132.3, 131.7, 128.7, 125.5, 125.3, 121.3, 119.6, 114.3, 106.5, 58.2, 20.9. HRMS (ESI): *m/z* $[M + H]^+$ calc. for $C_{17}H_{15}BrN_3O_3$: 388.0291; found: 388.0293.

**(6-bromo-4-((2-methyl-5-nitrophenyl)amino)quinolin-3-yl)methanol (4c)**

Prepared using the procedure described for **4a**. Compound **4c** was obtained as an orange solid (250 mg; 68% yield). $^1H$ NMR (300 MHz, $d^6$-DMSO): δ 9.03 (s, 1H), 8.20 (d, J = 2.1 Hz, 1H), 8.09 (s, 1H), 8.00 (d, J = 8.9 Hz, 1H), 7.87 (dd, J = 8.9, 2.2 Hz, 1H), 7.68 (dd, J = 8.3, 2.3 Hz, 1H), 7.50 (dd, J = 8.3, 0.9 Hz, 1H), 6.88 (d, J = 2.4 Hz, 1H), 5.47 (t, J = 5.5 Hz, 1H), 4.39, (d,

J = 5.5 Hz, 2H), 2.50 (s, 3H). $^{13}$C NMR (75.5 MHz, d$^6$-DMSO): δ 152.0, 147.0, 146.5, 144.4, 141.5, 134.5, 132.4, 131.7, 131.5, 128.2, 125.7, 125.2, 119.5, 115.0, 109.2, 58.3, 18.4. HRMS (ESI): *m/z* $[M + H]^+$ calc. for $C_{17}H_{15}BrN_3O_3$: 388.0291; found: 388.0293.

**(6-bromo-4-((3-nitrophenyl)amino)quinolin-3-yl)methanol (4d)**

Prepared using the procedure described for **4a**. Purification was carried out by column chromatography over silica-gel (eluent: MeOH:DCM 0:100 to 5:95) to give compound **4d** as a yellow solid (250 mg; 42% yield). $^1$H NMR (300 MHz, d$^6$-DMSO): δ 9.06 (d, J = 5.4 Hz, 2H), 8.21 (d, J = 2.0 Hz, 1H), 7.99 (d, J = 8.9 Hz, 1H), 7.87 (d, J = 8.9 Hz, 1H), 7.64 (d, J = 8.0 Hz, 1H), 7.49 – 7.42 (m, 2H), 7.00 (d, J = 8.0 Hz, 1H), 5.47 (t, J = 5.1 Hz, 1H), 4.48 (d, J = 5.1 Hz, 2H). $^{13}$C NMR (75.5 MHz, d$^6$-DMSO): δ 152.3, 148.6, 147.0, 146.3, 139.9, 132.4, 131.7, 130.5, 128.8, 125.5, 125.3, 120.9, 119.7, 113.7, 109.1, 58.2. HRMS (ESI): *m/z* $[M + H]^+$ calc. for $C_{16}H_{13}BrN_3O_3$: 374.0135; found: 374.0134.

**(6-bromo-4-((4-methoxy-3-nitrophenyl)amino)quinolin-3-yl)methanol (4e)**

Prepared using the procedure described for **4a**. Compound **4e** was obtained as an orange solid (600 mg; 93% yield). $^1$H NMR (300 MHz, d$^6$-DMSO): δ 8.97 (s, 1H), 8.67 (s, 1H), 8.26 (d, J = 2.1 Hz, 1H), 7.94 (d, J = 8.9 Hz, 1H), 7.83 (dd, J = 8.9, 2.1 Hz, 1H), 7.32 – 7.18 (m, 2H), 7.00 (dd, J = 9.0, 2.8 Hz, 1H), 5.40 (t, J = 5.4 Hz, 1H), 4.42 (d, J = 5.4 Hz, 2H), 3.85 (s, 3H). $^{13}$C NMR (75.5 MHz, d$^6$-DMSO): δ 152.2, 147.0, 146.0, 141.1, 139.3, 138.2, 132.1, 131.7, 131.7, 126.5, 125.5, 124.6, 122.3, 119.2, 115.5, 112.3, 58.5, 56.9. HRMS (ESI): *m/z* $[M + H]^+$ calc. for $C_{17}H_{15}BrN_3O_4$: 404.0240; found: 404.0244.

**(7-bromo-4-(4-methyl-3-nitrophenylamino)quinolin-3-yl)methanol** (**4a'**)

Prepared using the procedure described for **4a**. Compound **4a'** was isolated as an orange solid (770 mg; 100% yield). $^{1}$H NMR (300 MHz, d$^{6}$-DMSO): δ 9.00 (s, 1H), 8.83 (s, 1H), 8.23 (d, *J* = 2.0 Hz, 1H), 7.88 (d, *J* = 9.0 Hz, 1H), 7.68 (dd, *J* = 9.0, 2.1 Hz, 1H), 7.32 – 7.21 (m, 2H), 6.86 (dd, *J* = 8.4, 2.5 Hz, 1H), 5.43 (t, *J* = 5.4 Hz, 1H), 4.51 (d, *J* = 5.4 Hz, 2H), 2.39 (s, 3H). $^{13}$C NMR (75.5 MHz, d$^{6}$-DMSO): δ 152.9, 149.2, 149.1, 144.2, 141.7, 133.4, 131.3, 129.2, 127.8, 125.8, 123.0, 122.5, 122.4, 120.2, 110.6, 58.3, 18.9. HRMS (ESI): *m/z* $[M + H]^{+}$ calc. for $C_{17}H_{15}BrN_{3}O_{3}$: 388.0291; found: 388.0293.

**(7-bromo-4-(3-methyl-5-nitrophenylamino)quinolin-3-yl)methanol (4b')**

Prepared using the procedure described for **4a**. Compound **4b'** was isolated as a yellow solid (620 mg; 96% yield). $^{1}$H NMR (300 MHz, d$^{6}$-DMSO): δ 9.03 (s, 1H), 8.94 (s, 1H), 8.24 (d, *J* = 2.0 Hz, 1H), 7.88 (d, *J* = 9.0 Hz, 1H), 7.68 (dd, *J* = 9.0, 2.0 Hz, 1H), 7.48 – 7.45 (m, 1H), 7.25 (m, 1H), 6.84 (brs, 1H), 5.47 (t, *J* = 5.4 Hz, 1H), 4.52 (d, *J* = 5.4 Hz, 2H), 2.29 (s, 3H). HRMS (ESI): *m/z* $[M + H]^{+}$ calc. for $C_{17}H_{15}BrN_{3}O_{3}$: 388.0291; found: 388.0294.

**(7-bromo-4-(3-nitrophenylamino)quinolin-3-yl)methanol (4d')**

Prepared using the procedure described for **4a**. Compound **4d'** was isolated as an orange solid (810 mg; 91% yield). $^{1}$H NMR (300 MHz, d$^{6}$-DMSO): δ 9.03 (s, 2H), 8.25 (d, *J* = 2.0 Hz, 1H), 7.87 (d, *J* = 9.0 Hz, 1H), 7.69 (dd, *J* = 9.0, 2.1 Hz, 1H), 7.62 (ddd, *J* = 8.1, 2.2, 0.9 Hz, 1H), 7.48 – 7.44 (m, 1H), 7.41 (d, *J* = 8.1 Hz, 1H), 5.82 (s, 1H), 5.51 (t, *J* = 5.5 Hz, 1H), 4.52 (d, *J* = 5.5 Hz, 2H). HRMS (ESI): *m/z* $[M + H]^{+}$ calc. for $C_{16}H_{13}BrN_{3}O_{3}$: 374.0135; found: 374.0134.

*General Procedure C: Alcohol Oxidation to Aldehyde*

**6-bromo-4-((4-methyl-3-nitrophenyl)amino)quinoline-3-carbaldehyde (5a)**

The alcohol **4a** (2.34 g; 6.029 mmol) was suspended in DCM (150 ml) and the mixture cooled to 0ºC. DMP (3.83 g; 9.041 mmol; 1.5 equiv.) was added portionwise and the reaction warmed to rt. After 2h, TLC analysis (5% MeOH in DCM) showed that the reaction was completed. The solution was cooled to 0ºC, and NaOH (1 M) was slowly added. The mixture was stirred for 15 min at rt. $H_2O$ was added and the phases were separated. The aqueous phase was further extracted with DCM (3x). The combined organics were washed with brine, dried over MgSO4 and taken to dryness to afford the title compound as a yellow solid (1.75 g; 75% yield).

**6-bromo-4-((3-methyl-5-nitrophenyl)amino)quinoline-3-carbaldehyde (5b)**

Prepared using the procedure described for **5a**. Compound **5b** was obtained as an orange solid (420 mg; 72% yield). $^1$H NMR (300 MHz, $CDCl_3$): δ 11.26 (s, 1H), 10.10 (s, 1H), 8.90 (s, 1H), 7.97–7.88 (m, 2H), 7.83–7.74 (m, 2H), 7.66 (d, *J* = 2.1 Hz, 1H), 7.34–7.27 (m, 1H), 2.46 (s, 3H). $^{13}$C NMR (75.5 MHz, $CDCl_3$): δ 193.4, 155.0, 150.2, 149.7, 149.0, 141.6 (2), 136.0, 132.2, 129.5, 128.7, 121.4, 119.2, 118.9, 115.3, 113.7, 21.5. HRMS (ESI): *m/z* $[M + H]^+$ calc. for $C_{17}H_{13}BrN_3O_3$: 386.0135; found: 386.0142.

**6-bromo-4-((2-methyl-5-nitrophenyl)amino)quinoline-3-carbaldehyde (5c)**

Prepared using the procedure described for **5a**. Compound **5c** was obtained as a yellow solid (165 mg; 72% yield). $^1$H NMR (300 MHz, $d^6$-DMSO): δ 10.18 (s, 1H), 9.94 (s, 1H), 8.97 (s, 1H), 8.07-8.02 (m, 2H), 7.93-7.88 (m, 3H), 7.67 (m, 1H), 2.42 (s, 3H). $^1$H NMR (300 MHz, $CDCl_3$): δ 11.28 (s, 1H), 10.11 (s, 1H), 8.90 (s, 1H), 8.11 (dd, J = 8.4, 2.1 Hz, 1H), 7.91 (d, J =

8.9 Hz, 2H), 7.75 (dd, J = 9.0, 2.1 Hz, 1H), 7.55 (d, J = 8.4 Hz, 1H), 7.45 (d, J = 1.9 Hz, 1H), 2.48 (s, 3H). HRMS (ESI): *m/z* $[M + H]^+$ calc. for $C_{17}H_{13}BrN_3O_3$: 386.0135; found: 386.0136.

**6-bromo-4-((3-nitrophenyl)amino)quinoline-3-carbaldehyde (5d)**

Prepared using the procedure described for **5a**. Compound **5d** was obtained as an orange solid (200 mg; 65% yield). $^1$H NMR (300 MHz, $CDCl_3$): δ 11.27 (s, 1H), 10.12 (s, 1H), 8.93 (s, 1H), 8.14 (dd, J = 8.1, 2.2 Hz, 1H), 8.03 (t, J =2.2 Hz, 1H), 7.93 (d, J = 9.0 Hz, 1H), 7.78 (dd, J = 9.0, 2.1 Hz, 1H), 7.64 (d, J = 2.1 Hz, 1H), 7.60-7.53 (m, 1H), 7.45 (d, J = 8.0 Hz, 1H). $^{13}$C NMR (75.5 MHz, $CDCl_3$): δ 193.4, 154.9 150.1, 149.7, 149.1, 142.0, 136.0, 132.2, 130.6, 128.8, 128.6, 120.8, 119.2, 119.1, 118.0, 113.9. HRMS (ESI): *m/z* $[M + H]^+$ calc. for $C_{16}H_{11}BrN_3O_3$: 371.9978; found: 371.9986.

**6-bromo-4-((4-methoxy-3-nitrophenyl)amino)quinoline-3-carbaldehyde (5e)**

Prepared using the procedure described for **5a**. Compound **5e** was obtained as an orange solid (370 mg; 70% yield). $^1$H NMR (300 MHz, $CDCl_3$): δ 11.33 (s, 1H), 10.07 (s, 1H), 8.84 (s, 1H), 7.88 (dd, J = 8.9, 2.1 Hz, 1H), 7.77-7.70 (m, 2H), 7.63 (d, J = 2.1 Hz, 1H), 7.38 (dd, J = 8.8, 2.7 Hz, 1H), 7.14 (d, J = 8.9 Hz, 1H), 4.02 (s, 3H). $^{13}$C NMR (75.5 MHz, $CDCl_3$): δ 193.3, 155.2, 151.6, 151.0, 149.7, 139.7, 135.8, 133.0, 132.1, 129.9, 128.7, 121.5, 119.0, 118.8, 114.8, 112.9, 57.1. HRMS (ESI): *m/z* $[M + H]^+$ calc. for $C_{17}H_{13}BrN_3O_4$: 402.0084; found: 402.0088.

**7-bromo-4-(4-methyl-3-nitrophenylamino)quinoline-3-carbaldehyde (5a')**

Prepared using the procedure described for **5a**. Compound **5a'** was obtained as a brown solid (220 mg; 85% yield). $^1$H NMR (300 MHz, $CDCl_3$): δ 11.29 (s, 1H), 10.06 (s, 1H), 8.85 (s, 1H), 8.18 (d, *J* = 2.0 Hz, 1H), 7.80 (d, *J* = 2.4 Hz, 1H), 7.40 - 7.32 (m, 2H), 7.30 – 7.24 (m, 2H), 2.62 (s, 3H). $^{13}$C NMR (75.5 MHz, $CDCl_3$): δ 193.3, 155.9, 151.8, 151.6, 149.7, 139.8, 134.1,

132.9, 131.4, 128.8, 127.9, 127.5, 127.4, 119.5, 116.6, 113.3, 20.2. HRMS (ESI): *m/z* $[M + H]^+$ calc. for $C_{17}H_{13}BrN_3O_3$: 386.0135; found: 386.0135.

**7-bromo-4-(3-methyl-5-nitrophenylamino)quinoline-3-carbaldehyde (5b')**

Prepared using the procedure described for **5a**. Compound **5b'** was obtained as an orange solid (480 mg; 82% yield). $^1H$ NMR (300 MHz, $d^6$-DMSO): δ 10.37 (s, 1H), 10.11 (s, 1H), 9.03 (s, 1H), 8.22 (d, $J$ = 2.1 Hz, 1H), 7.92 (d, $J$ = 9.1 Hz, 1H), 7.78 – 7.75 (m, 2H), 7.67 (dd, $J$ = 9.0, 2.1 Hz, 1H), 7.38 (s, 1H), 2.34 (s, 3H). HRMS (ESI): *m/z* $[M + H]^+$ calc. for $C_{17}H_{13}BrN_3O_3$: 386.0135; found: 386.0127.

**7-bromo-4-(3-nitrophenylamino)quinoline-3-carbaldehyde (5d')**

Prepared using the procedure described for **5a**. Compound **5d'** was obtained as a brown solid (790 mg; 99% yield). $^1H$ NMR (300 MHz, $CDCl_3$): δ 11.32 (s, 1H), 10.10 (s, 1H), 8.92 (s, 1H), 8.23 (d, $J$ = 2.0 Hz, 1H), 8.17 – 8.05 (m, 1H), 8.03 – 8.01 (m, 1H), 7.55 (t, $J$ = 8.1 Hz, 1H), 7.50 – 7.41 (m, 1H), 7.38 (d, $J$ = 9.1 Hz, 1H), 7.29 (dd, $J$ = 9.1, 2.0 Hz, 1H). HRMS (ESI): *m/z* $[M + H]^+$ calc. for $C_{16}H_{11}BrN_3O_3$: 371.9978; found: 371.9975.

*General Procedure D: Horner-Wadsworth-Emmons (HWE) cyclization*

**9-bromo-1-(4-methyl-3-nitrophenyl)benzo[h][1,6]naphthyridin-2(1H)-one (6a)**

The aldehyde **5a** (1.74 g; 4.5 mmol), triethylphosphonoacetate (894 µL; 4.5 mmol) and $K_2CO_3$ (1.87 g; 13.516 mmol; 3 equiv.) were mixed in dry EtOH (30 ml) in a sealed tube under Argon. The mixture was heated to 100ºC overnight. After 16h, the reaction was cooled to rt and the solvent evaporated. The crude was partitioned between $H_2O$ and EtOAc. The aqueous phase was further extracted with EtOAc (3x) and the combined organics washed with brine,

dried over $MgSO_4$ and taken to dryness to afford the title compound as a dark brown solid (1.62 g; 88% yield).

**9-bromo-1-(3-methyl-5-nitrophenyl)benzo[h][1,6]naphthyridin-2(1H)-one (6b)**

Prepared using the procedure described for **6a**. Compound **6b** was obtained as a brown solid (320 mg; 74% yield). $^1$H NMR (300 MHz, $CDCl_3$): δ 8.97 (s, 1H), 8.33 (s, 1H), 8.09 – 7.88 (m, 3H), 7.67 (dd, *J* = 8.9, 2.1 Hz, 1H), 7.53 (s, 1H), 6.95 (d, *J* = 9.5 Hz, 1H), 6.80 (d, *J* = 2.1 Hz, 1H), 2.58 (s, 3H). $^{13}$C NMR (75.5 MHz, $CDCl_3$): δ 162.9, 150.8, 149.4, 148.2, 142.7, 141.2, 140.6, 139.7, 135.6, 133.5, 132.6, 127.5, 125.1, 122.9, 121.4, 120.3, 118.44, 113.8, 21.5. HRMS (ESI): *m/z* $[M + H]^+$ calc. for $C_{19}H_{13}BrN_3O_3$: 410.0135; found: 410.0132.

**9-bromo-1-(2-methyl-5-nitrophenyl)benzo[h][1,6]naphthyridin-2(1H)-one (6c)**

Prepared using the procedure described for **6a**. Compound **6c** was obtained as a dark brown solid (165 mg; 68% yield). $^1$H NMR (300 MHz, $CDCl_3$): δ 9.02 (s, 1H), 8.45 (dd, *J* = 8.5, 2.4 Hz, 1H), 8.11 – 7.97 (m, 3H), 7.74 – 7.68 (m, 2H), 7.00 (d, *J* = 9.5 Hz, 1H), 6.80 (d, *J* = 2.0 Hz, 1H), 2.21 (s, 3H). HRMS (ESI): *m/z* $[M + H]^+$ calc. for $C_{19}H_{13}BrN_3O_3$: 410.0135; found: 410.0133.

**9-bromo-1-(3-nitrophenyl)benzo[h][1,6]naphthyridin-2(1H)-one (6d)**

Prepared using the procedure described for **6a**. Compound **6d** was obtained as a dark brown solid (150 mg; 71% yield). $^1$H NMR (300 MHz, $CDCl_3$): δ 9.01 (s, 1H), 8.53 (dd, *J* = 8.3, 2.1 Hz, 1H), 8.21 (t, *J* = 2.1 Hz, 1H), 8.00 (dd, *J* = 10.4, 9.2 Hz, 2H), 7.87 (t, *J* = 8.1 Hz, 1H), 7.80 – 7.64 (m, 2H), 6.97 (d, *J* = 9.5 Hz, 1H), 6.80 (d, *J* = 2.1 Hz, 1H). HRMS (ESI): *m/z* $[M + H]^+$ calc. for $C_{18}H_{11}BrN_3O_3$: 395.9978; found: 395.9976.

**9-bromo-1-(4-methoxy-3-nitrophenyl)benzo[h][1,6]naphthyridin-2(1H)-one (6e)**

Prepared using the procedure described for **6a**. Compound **6e** was obtained as a dark brown solid (255 mg; 65% yield). $^1$H NMR (300 MHz, d$^6$-DMSO): δ 9.17 (s, 1H), 8.32 (d, *J* = 9.5 Hz, 1H), 8.17 (d, *J* = 2.5 Hz, 1H), 7.98 (d, *J* = 8.9 Hz, 1H), 7.82 (m, 2H), 7.69 (d, *J* = 9.0 Hz, 1H), 6.96 (d, *J* = 9.4 Hz, 1H), 6.83 (d, *J* = 2.0 Hz, 1H), 4.07 (s, 3H). HRMS (ESI): *m/z* $[M + H]^+$ calc. for $C_{19}H_{13}BrN_3O_4$: 426.0084; found: 426.0090.

**8-bromo-1-(4-methyl-3-nitrophenyl)benzo[h][1,6]naphthyridin-2(1H)-one (6a')**

Prepared using the procedure described for **6a**. Compound **6a'** was obtained as a brown solid (220 mg; 96% yield). $^1$H NMR (300 MHz, d$^6$-DMSO): δ 9.17 (s, 1H), 8.31 (d, *J* = 9.5 Hz, 1H), 8.23 (m, 2H), 7.81 – 7.68 (m, 2H), 7.39 (dd, *J* = 9.4, 2.3 Hz, 1H), 6.94 (d, *J* = 9.5 Hz, 1H), 6.73 (d, *J* = 9.4 Hz, 1H), 2.68 (s, 3H). HRMS (ESI): *m/z* $[M + H]^+$ calc. for $C_{19}H_{13}BrN_3O_3$: 410.0135; found: 410.0134.

**8-bromo-1-(3-methyl-5-nitrophenyl)benzo[h][1,6]naphthyridin-2(1H)-one (6b')**

Prepared using the procedure described for **6a**. Compound **6b'** was obtained as a brown solid (345 mg; 81% yield). $^1$H NMR (300 MHz, d$^6$-DMSO): δ 9.18 (s, 1H), 8.36 – 8.27 (m, 3H), 8.24 (d, *J* = 2.3 Hz, 1H), 7.76 (brs, 1H), 7.36 (dd, *J* = 9.5, 2.3 Hz, 1H), 6.95 (d, *J* = 9.4 Hz, 1H), 6.69 (d, *J* = 9.4 Hz, 1H), 2.48 (s, 3H). HRMS (ESI): *m/z* $[M + H]^+$ calc. for $C_{19}H_{13}BrN_3O_3$: 410.0135; found: 410.0132.

**8-bromo-1-(3-nitrophenyl)benzo[h][1,6]naphthyridin-2(1H)-one (6d')**

Prepared using the procedure described for **6a**. Compound **6d'** was obtained as a brown solid (500 mg; 60% yield). $^1$H NMR (300 MHz, d$^6$-DMSO): δ 9.18 (s, 1H), 8.49 (m, 2H), 8.32 (d, *J* = 9.5 Hz, 1H), 8.24 (d, *J* = 2.3 Hz, 1H), 7.91 – 7.89 (m, 2H), 7.34 (dd, *J* = 9.4, 2.3 Hz, 1H),

6.95 (d, $J$ = 9.5 Hz, 1H), 6.65 (d, $J$ = 9.4 Hz, 1H). HRMS (ESI): $m/z$ $[M + H]^+$ calc. for $C_{18}H_{11}BrN_3O_3$: 395.9978; found: 395.9975

*General Procedure E: Suzuki Coupling*

**N-(4-(1-(4-methyl-3-nitrophenyl)-2-oxo-1,2-dihydrobenzo[h][1,6]naphthyridin-9-yl)phenyl)methanesulfonamide (7a)**

The bromo-quinoline **6a** (280 mg; 0.68 mmol), 4-(methanesulfonylamino) phenyl boronic acid pinacol ester (243 mg; 0.82 mmol; 1.2 equiv.), $PdCl_2(PPh_3)_2$ (48 mg; 0.068 mmol; 0.1 equiv.) and $Na_2CO_3$ (1 mL; 2 M, 2.049 mmol; 3 equiv.) were mixed in dioxane (3 mL) under Argon. The mixture was heated to 90 ºC overnight. After 16 h, TLC analysis (MeOH:DCM 5:95) showed that the reaction was completed. The mixture was cooled to RT and filtered through a Celite pad. The pad was further washed with EtOH and MeOH/DCM (10%) until no more product was detected by TLC. The solvent was evaporated and the crude applied in a silica column with a gradient up to 2% MeOH in DCM. The desired fractions were collected and evaporated to dryness to afford the title compound as a yellow solid (455 mg; 69%).

**N-(4-(1-(3-methyl-5-nitrophenyl)-2-oxo-1,2-dihydrobenzo[h][1,6]naphthyridin-9-yl)phenyl)methanesulfonamide (7b)**

Prepared using the procedure described for **7a**. Compound **7b** was obtained as a dark yellow solid (160 mg: 64% yield). $^1H$ NMR (300 MHz, $d^6$-DMSO): δ 9.92 (brs, 1H), 9.15 (s, 1H), 8.45 – 8.28 (m, 3H), 8.10 (d, $J$ = 8.6 Hz, 1H), 7.98 (dd, $J$ = 8.7, 1.9 Hz, 1H), 7.89 (s, 1H), 7.20 (d, $J$ = 8.6 Hz, 2H), 7.15 – 7.02 (m, 3H), 6.95 (d, $J$ = 9.5 Hz, 1H), 3.03 (s, 3H), 2.5 (s, 3H). $^1H$ NMR (300 MHz, $CDCl_3$): δ 9.01 (s, 1H), 8.29 (s, 1H), 8.19 (d, $J$ = 8.7 Hz, 1H), 8.09 (m, 1H), 8.03 (d, $J$ = 9.5 Hz, 1H), 7.86 (dd, $J$ = 8.7, 1.9 Hz, 1H), 7.59 (s, 1H), 7.19 (d, $J$ = 8.6 Hz, 2H), 7.12

(d, $J$ = 1.9 Hz, 1H), 7.04 (d, $J$ = 8.6 Hz, 2H), 6.96 (d, $J$ = 9.4 Hz, 1H), 6.57 (s, 1H), 3.07 (s, 4H), 2.56 (s, 3H). HRMS (ESI): $m/z$ $[M + H]^+$ calc. for $C_{26}H_{21}N_4O_5S$: 501.1227; found: 501.1224.

**N-(4-(1-(2-methyl-5-nitrophenyl)-2-oxo-1,2-dihydrobenzo[h][1,6]naphthyridin-9-yl)phenyl)methanesulfonamide (7c)**

Prepared using the procedure described for **7a**. Compound **7c** was obtained as an orange solid (145 mg; 77% yield). $^1$H NMR (300 MHz, d$^6$-acetone): δ 9.14 (s, 1H), 8.74 (brs, 1H), 8.53 – 8.44 (m, 2H), 8.34 (d, $J$ = 9.5 Hz, 1H), 8.15 (d, $J$ = 8.6 Hz, 1H), 8.03 – 7.91 (m, 2H), 7.38 – 7.31 (m, 2H), 7.23 – 7.13 (m, 3H), 6.95 (d, $J$ = 9.5 Hz, 1H), 3.04 (s, 3H), 2.23 (s, 3H). HRMS (ESI): $m/z$ $[M + H]^+$ calc. for $C_{26}H_{21}N_4O_5S$: 501.1227; found: 501.1223.

**N-(4-(1-(3-nitrophenyl)-2-oxo-1,2-dihydrobenzo[h][1,6]naphthyridin-9-yl)phenyl)methanesulfonamide (7d)**

Prepared using the procedure described for **7a**. Compound **7d** was obtained as a yellow solid (85 mg; 51% yield). $^1$H NMR (300 MHz, d$^6$-DMSO): δ 9.93 (s, 1H), 9.15 (s, 1H), 8.62 (s, 1H), 8.58 – 8.47 (m, 1H), 8.34 (d, $J$ = 9.5 Hz, 1H), 8.10 (d, $J$ = 8.7 Hz, 1H), 7.97 (m, 3H), 7.18 (d, $J$ = 8.2 Hz, 2H), 7.10 – 6.99 (m, 3H), 6.96 (d, $J$ = 9.4 Hz, 1H), 3.03 (s, 3H). HRMS (ESI): $m/z$ $[M + H]^+$ calc. for $C_{25}H_{19}N_4O_5S$: 487.1071; found: 487.1071.

**N-(4-(1-(4-methoxy-3-nitrophenyl)-2-oxo-1,2-dihydrobenzo[h][1,6]naphthyridin-9-yl)phenyl)methanesulfonamide (7e)**

Prepared using the procedure described for **7a**. Compound **7e** was obtained as a dark yellow solid (120 mg; 42% yield). $^1$H NMR (300 MHz, d$^6$-acetone): δ 9.06 (s, 1H), 8.83 – 8.74 (brs, 1H), 8.24 (d, $J$ = 9.5 Hz, 1H), 8.15 – 8.09 (m, 2H), 7.99 (dd, $J$ = 8.7, 1.9 Hz, 1H), 7.82 (dd, $J$

= 8.9, 2.6 Hz, 1H), 7.64 (d, *J* = 9.0 Hz, 1H), 7.40 (d, *J* = 8.6 Hz, 2H), 7.30 (d, *J* = 2.2 Hz, 1H), 7.27 (d, *J* = 2.1 Hz, 1H), 7.21 (dd, *J* = 1.9, 0.6 Hz, 1H), 6.88 (d, *J* = 9.5 Hz, 1H), 4.14 (s, 3H), 3.06 (d, *J* = 0.9 Hz, 3H). HRMS (ESI): m/z [M + H]+ calc. for $C_{26}H_{21}N_4O_6S$: 517.1176; found: 517.1176.

**N-(4-(1-(4-methyl-3-nitrophenyl)-2-oxo-1,2-dihydrobenzo[h][1,6]naphthyridin-8-yl)phenyl)methanesulfonamide (7a')**

Prepared using the procedure described for **7a**. Compound **7a'** was obtained as an orange solid (76 mg; 21%). $^{1}$H NMR (300 MHz, d$^{6}$-DMSO): δ 9.97 (s, 1H), 9.16 (s, 1H), 8.33 – 8.30 (m, 2H), 8.26 (s, 1H), 7.84 – 7.77 (m, 4H), 7.57 (dd, *J* = 9.3, 2.2 Hz, 1H), 7.30 (d, *J* = 8.7 Hz, 2H), 6.92 – 6.86 (m, 2H), 3.04 (s, 3H), 2.69 (s, 3H). HRMS (ESI): *m/z* $[M + H]^+$ calc. for $C_{26}H_{21}N_4O_5S$: 501.1227; found: 501.1227.

**N-(4-(1-(3-methyl-5-nitrophenyl)-2-oxo-1,2-dihydrobenzo[h][1,6]naphthyridin-8-yl)phenyl)methanesulfonamide (7b')**

Prepared using the procedure described for **7a**. Compound **7b'** was obtained as an orange solid (400 mg; 95%). HRMS (ESI): *m/z* $[M + H]^+$ calc. for $C_{26}H_{21}N_4O_5S$: 501.1227; found: 501.1232.

**N-(4-(1-(3-nitrophenyl)-2-oxo-1,2-dihydrobenzo[h][1,6]naphthyridin-8-yl)phenyl)methanesulfonamide (7d')**

Prepared using the procedure described for **7a**. Compound **7d'** was obtained as an orange solid (90 mg; 31%). $^{1}$H NMR (300 MHz, d$^{6}$-DMSO): δ 9.92 (brs, 1H), 9.16 (s, 1H), 8.52 – 8.49 (m, 2H), 8.34 – 8.29 (m, 2H), 7.95 – 7.93 (m, 2H), 7.79 (d, *J* = 8.8 Hz, 2H), 7.52 (dd, *J* = 9.3,

2.2 Hz, 1H), 7.29 (d, *J* = 8.8 Hz, 2H), 6.91 (d, *J* = 9.4 Hz, 1H), 6.76 (d, *J* = 9.3 Hz, 1H), 3.03 (s, 3H). HRMS (ESI): *m/z* $[M + H]^+$ calc. for $C_{25}H_{19}N_4O_5S$: 487.1071; found: 487.1069.

**Ethyl 4-(4-methyl-3-nitrophenylamino)-6-(4-(methylsulfonamido)phenyl)quinoline-3-carboxylate (Intermediate 1 – Scheme S3)**

Prepared using the procedure described for **7a**, from intermediate **3a**. Compound **INT1** was obtained as a yellow solid (265 mg; 73%). $^1H$ NMR (300 MHz, $CDCl_3$): δ 10.52 (s, 1H), 9.29 (s, 1H), 8.10 (d, *J* = 8.7 Hz, 1H), 7.87 (dd, *J* = 8.7, 2.0 Hz, 1H), 7.72 (d, *J* = 2.0 Hz, 1H), 7.67 (d, *J* = 2.5 Hz, 1H), 7.59 – 7.41 (m, 2H), 7.20 - 7.16 (m, 5H), 4.46 (q, *J* = 7.1 Hz, 2H), 3.03 (s, 3H), 2.58 (s, 3H), 1.47 (t, *J* = 7.1 Hz, 3H). HRMS (ESI): *m/z* $[M + H]^+$ calc. for $C_{26}H_{25}N_4O_6S$: 521.1489; found: 521.1486.

**N-(4-(1-(4-methyl-3-nitrophenyl)-2-oxo-1,2-dihydrobenzo[h][1,6]naphthyridin-9-yl)phenyl)butane-1-sulfonamide (7f – precursor of 14)**

Prepared using General Procedure E, reacting intermediate **6a** with 4-(butylsulfonamido)phenylboronic acid. Compound **7f** was isolated as a yellow solid (200 mg; 53% yield). $^1H$ NMR (300 MHz, $d^6$-DMSO): δ 9.98 (s, 1H), 9.13 (s, 1H), 8.44 – 8.26 (m, 2H), 8.09 (d, *J* = 8.7 Hz, 1H), 7.98 (d, *J* = 8.6 Hz, 1H), 7.82-7.74 (m, 2H), 7.24 (d, *J* = 8.2 Hz, 2H), 7.09 (d, *J* = 8.2 Hz, 2H), 6.95 (d, *J* = 8.2 Hz, 2H), 3.12 (t, *J* = 7.9 Hz, 3H), 2.65 (s, 3H), 1.65 (m, 2H), 1.36 (m, 2H), 0.84 (t, *J* = 7.2 Hz, 3H). HRMS (ESI): *m/z* $[M + H]^+$ calc. for $C_{29}H_{27}N_4O_5S$: 543.1697; found: 543.1694.

**N-(3-(1-(4-methyl-3-nitrophenyl)-2-oxo-1,2-dihydrobenzo[h][1,6]naphthyridin-9-yl)phenyl)methanesulfonamide (7g – precursor of 15)**

Prepared using General Procedure E, reacting intermediate **6a** with 3-(methanesulfonylamino)phenylboronic acid pinacol ester. Compound **7g** was isolated as an orange solid (235 mg; 67% yield). $^1$H NMR (300 MHz, d$^6$-DMSO): δ 9.81 (s, 1H), 9.16 (s, 1H), 8.34 (d, *J* = 9.5 Hz, 1H), 8.29 (m, 1H), 8.15 (d, *J* = 8.6 Hz, 1H), 7.91 (dd, *J* = 8.7, 1.9 Hz, 1H), 7.79 (d, *J* = 1.3 Hz, 2H), 7.35 (t, *J* = 7.8 Hz, 1H), 7.27 – 7.15 (m, 2H), 7.05 (d, *J* = 1.8 Hz, 1H), 6.95 (d, *J* = 9.4 Hz, 1H), 6.58 (dt, *J* = 8.0, 1.2 Hz, 1H), 3.00 (s, 3H), 2.66 (s, 3H). HRMS (ESI): *m/z* $[M + H]^+$ calc. for $C_{26}H_{21}N_4O_5S$: 501.1227; found: 501.1228.

**Methyl 4-(1-(4-methyl-3-nitrophenyl)-2-oxo-1,2-dihydrobenzo[h][1,6]naphthyridin-9-yl)phenylcarbamate (7h – precursor of 16)**

Prepared using General Procedure E, reacting intermediate **6a** with 4-(methoxycarbonylamino)benzeneboronic acid. Compound **7h** was isolated as an orange solid (166 mg; 50% yield). $^1$H NMR (300 MHz, d$^6$-DMSO): δ 9.82 (s, 1H), 9.13 (s, 1H), 8.33 (dd, *J* = 9.4, 1.5 Hz, 2H), 8.08 (dd, *J* = 8.6, 1.4 Hz, 1H), 8.04 – 7.93 (m, 1H), 7.86 – 7.72 (m, 2H), 7.56 – 7.46 (m, 2H), 7.06 (dd, *J* = 8.7, 1.5 Hz, 2H), 7.03 – 6.91 (m, 2H), 3.69 (s, 3H), 2.68 (s, 3H). HRMS (ESI): *m/z* $[M + H]^+$ calc. for $C_{27}H_{21}N_4O_5$: 481.1506; found: 481.1507.

**1-(4-methyl-3-nitrophenyl)-9-(pyridin-4-yl)benzo[h][1,6]naphthyridin-2(1H)-one (7i – precursor of 17)**

Prepared using General Procedure E, reacting intermediate **6a** with pyridine-4-boronic acid hydrate. Compound **7i** was isolated as an orange solid (90 mg; 45% yield). $^1$H NMR (300 MHz, d$^6$-DMSO): δ 9.21 (s, 1H), 8.64 – 8.56 (m, 2H), 8.40 – 8.31 (m, 2H), 8.20 – 8.14 (m, 1H), 8.11

(dd, $J$ = 8.7, 1.9 Hz, 1H), 7.87 – 7.75 (m, 3H), 7.15 – 7.12 (m, 2H), 6.98 (d, $J$ = 9.5 Hz, 1H), 2.66 (s, 3H). HRMS (ESI): *m/z* $[M + H]^+$ calc. for $C_{24}H_{17}N_4O_3$: 409.1295; found: 409.1293.

**5-(1-(4-methyl-3-nitrophenyl)-2-oxo-1,2-dihydrobenzo[h][1,6]naphthyridin-9-yl)picolinonitrile (7j – precursor of 18)**

Prepared using General Procedure E, reacting intermediate **6a** with 6-(cyanopyridin-3-yl)boronic acid. Compound **7i** was isolated as an orange solid (92 mg; 44% yield). $^1$H NMR (300 MHz, d$^6$-DMSO): δ 9.22 (s, 1H), 8.50 (m, 1H), 8.36 (d, $J$ = 9.5 Hz, 1H), 8.29 (s, 1H), 8.20 (d, $J$ = 8.6 Hz, 1H), 8.16 - 8.14 (m, 1H), 7.80 (m, 2H), 7.65 – 7.52 (m, 3H), 6.98 (d, $J$ = 9.5 Hz, 1H), 2.66 (s, 3H). HRMS (ESI): *m/z* $[M + H]^+$ calc. for $C_{25}H_{16}N_5O_3$: 434.1248; found: 434.1245.

**1-(4-methyl-3-nitrophenyl)-9-(6-(trifluoromethyl)pyridin-3-yl)benzo[h][1,6]naphthyridin-2(1H)-one (7k – precursor of 19)**

Prepared using General Procedure E, reacting intermediate **6a** with 2-Trifluoromethyl(pyridin-5-yl)boronic acid. Compound **7i** was isolated as an orange solid (110 mg; 47% yield). $^1$H NMR (300 MHz, d$^6$-DMSO): δ 9.22 (s, 1H), 8.53 (d, $J$ = 2.1 Hz, 1H), 8.36 (d, $J$ = 9.5 Hz, 1H), 8.29 (s, 1H), 8.20 (d, $J$ = 8.7 Hz, 1H), 8.13 (dd, $J$ = 8.7, 1.9 Hz, 1H), 7.95 (d, $J$ = 8.2 Hz, 1H), 7.83 (m, 1H), 7.65 – 7.54 (m, 3H), 6.97 (s, 1H), 2.62 (s, 3H). HRMS (ESI): *m/z* $[M + H]^+$ calc. for $C_{25}H_{16}F_3N_4O_3$: 477.1169; found: 477.1168.

*General Procedure F: Buchwald-Hartwig Coupling*

**1-(4-methyl-3-nitrophenyl)-9-(piperidin-1-yl)benzo[h][1,6]naphthyridin-2(1H)-one (7l - precursor of 20)**

The bromo-quinoline **6a** (250 mg; 0.609 mmol), piperidine (180 µL; 1.827 mmol; 3 equiv.), $Pd(OAc)_2$ (8.4 mg; 37.4 µmol; 0.06 equiv.), R-BINAP (46 mg: 73.1 µmol; 0.12 equiv.) and $Cs_2CO_3$ (595 mg,1.827 mmol; 3 equiv.) were mixed in dioxane (5 mL) under Argon. The mixture was heated to 90ºC overnight. After 24h, LCMS analysis showed that the reaction was completed. The mixture was cooled to rt and the solvent evaporated. The crude was partitioned between EtOAc and sat $NaHCO_3$. The phases were separated and the aqueous phase further extracted with EtOAc (2x). The combined organics were washed with brine and dried over $MgSO_4$. After filtration, the solvent was evaporated to afford the title compound as a yellow solid. The compound was used without further purification (180 mg; 71% yield). $^1H$ NMR (300 MHz, $d^6$-DMSO): δ 8.87 (s, 1H), 8.26 – 8.18 (m, 2H), 7.84 (d, $J$ = 9.2 Hz, 1H), 7.76 (d, $J$ = 8.2 Hz, 1H), 7.70 (dd, $J$ = 8.2, 2.1 Hz, 1H), 7.50 - 7.46 (m, 1H), 6.85 (d, $J$ = 9.4 Hz, 1H), 6.23 (d, $J$ = 2.6 Hz, 1H), 2.74 (m, 4H), 2.60 (s, 3H), 1.45 (m, 6H). HRMS (ESI): *m/z* $[M + H]^+$ calc. for $C_{24}H_{23}N_4O_3$: 415.1765; found: 415.1763.

**1-(4-methyl-3-nitrophenyl)-9-morpholinobenzo[h][1,6]naphthyridin-2(1H)-one (7m – precursor of 21)**

Prepared using General Procedure F, using morpholine as amine. Compound **7m** was isolated as an orange solid (290 mg; 95% yield). $^1H$ NMR (300 MHz, $d^6$-DMSO): δ 8.91 (s, 1H), 8.35 – 8.17 (m, 2H), 7.89 (d, $J$ = 9.2 Hz, 1H), 7.81 – 7.65 (m, 2H), 7.53 (dd, $J$ = 9.2, 2.6 Hz, 1H), 6.87 (d, $J$ = 9.4 Hz, 1H), 6.23 (d, $J$ = 2.6 Hz, 1H), 3.62 (m, 4H), 2.68 (m, 4H), 2.58 (s, 3H). HRMS (ESI): *m/z* $[M + H]^+$ calc. for $C_{23}H_{21}N_4O_4$: 417.1557; found: 417.1557.

**1-(4-methyl-3-nitrophenyl)-9-(4-(methylsulfonyl)piperazin-1-yl)benzo[h][1,6]naphthyridin-2(1H)-one (7n– precursor of 22)**

Prepared using General Procedure F, using 1-(Methylsulfonyl)piperazine as amine. Compound **7n** was isolated as a yellow solid (285 mg; 95% yield). $^1$H NMR (300 MHz, $CDCl_3$): δ 8.83 (s, 1H), 8.10 – 7.86 (m, 3H), 7.69 – 7.52 (m, 2H), 7.35 (dd, *J* = 9.3, 2.6 Hz, 1H), 6.90 (d, *J* = 9.3 Hz, 1H), 6.36 (d, *J* = 2.6 Hz, 1H), 3.25 (t, *J* = 5.0 Hz, 4H), 2.95 – 2.89 (m, 2H), 2.85 – 2.82 (m, 2H), 2.82 (s, 3H), 2.71 (s, 3H).HRMS (ESI): *m/z* $[M + H]^+$ calc. for $C_{24}H_{24}N_5O_5S$: 494.1493; found: 494.1492.

**9-(4-(dimethylamino)piperidin-1-yl)-1-(4-methyl-3-nitrophenyl)benzo[h][1,6]naphthyridin-2(1H)-one (7o – precursor of 23)**

Prepared using General Procedure F, using N,N-dimethylpiperidin-4-amine as amine. Compound **7o** was isolated as a yellow solid (215 mg; 77% yield). $^1$H NMR (300 MHz, $d^6$-DMSO): δ 8.87 (s, 1H), 8.23 (m, 2H), 7.85 (d, *J* = 9.2 Hz, 1H), 7.77 (d, *J* = 8.4 Hz, 1H), 7.70 (dd, *J* = 8.1, 2.1 Hz, 1H), 7.51 (m, 1H), 6.85 (d, *J* = 9.4 Hz, 1H), 6.25 (d, *J* = 2.6 Hz, 1H), 3.23 – 3.18 (m, 2H), 2.62 (s, 3H), 2.44 – 2.37 (m, 2H), 2.15 (s, 3H), 2.15 (m, 1H), 1.66 – 1.62 (m, 2H), 1.28 – 1.23 (m, 2H). HRMS (ESI): *m/z* $[M + H]^+$ calc. for $C_{26}H_{28}N_5O_3$: 458.2187; found: 458.2185.

**8-(4-(methylsulfonyl)piperazin-1-yl)-1-(3-nitrophenyl)benzo[h][1,6]naphthyridin-2(1H)-one (7d'n'- precursor of 27)**

Prepared using the General Procedure F, reacting intermediate **6d** with 1-(Methylsulfonyl)piperazine. Compound **7d'n'** was obtained as a brown solid (355 mg; 98%). HRMS (ESI): *m/z* $[M + H]^+$ calc. for $C_{23}H_{22}N_5O_5S$: 480.1336; found: 480.1330.

*General Procedure G: Nitro to amine reduction with Fe/$NH_4Cl$*

**N-(4-(1-(3-amino-4-methylphenyl)-2-oxo-1,2-dihydrobenzo[h][1,6]naphthyridin-9-yl)phenyl)methanesulfonamide (8a)**

Intermediate **7a** (335 mg; 0.67 mmol) was suspended in EtOH (40 mL) and heated to reflux. Fe (224 mg; 4.0 mmol; 6 equiv.) and $NH_4Cl$ (215 mg; 4.016 mmol; 6 equiv.) in $H_2O$ (20 mL) were added and the mixture heated to reflux. After 2h, TLC analysis (MeOH:DCM 1:9) showed that the reaction was completed. The hot mixture was filtered through a Celite pad and the pad further washed with EtOH and MeOH:DCM (2:8). The solvent was evaporated and the crude partitioned between $H_2O$ and EtOAc. The phases were separated and the aqueous phase was further extracted with EtOAc (3x). The combined organics were washed with brine, dried over $MgSO_4$ and taken to dryness to afford the title compound as an off-white solid (330 mg; 100% yield).

**N-(4-(1-(3-amino-5-methylphenyl)-2-oxo-1,2-dihydrobenzo[h][1,6]naphthyridin-9-yl)phenyl)methanesulfonamide (8b)**

Prepared using the procedure described for **8a**. Compound **8b** was obtained as a bright-yellow solid (85 mg; 70% yield). $^1$H NMR (300 MHz, d$^6$-DMSO): δ 9.90 (s, 1H), 9.07 (s, 1H), 8.25 (d, *J* = 9.4 Hz, 1H), 8.03 (q, *J* = 8.9 Hz, 2H), 7.70 (s, 1H), 7.35 – 7.21 (m, 4H), 6.87 (d, *J* = 9.5 Hz, 1H), 6.66 (s, 1H), 6.48 (s, 1H), 6.29 (s, 1H), 5.40 (s, 2H), 3.04 (s, 3H), 2.25 (s, 3H). HRMS (ESI): *m/z* $[M + H]^+$ calc. for $C_{26}H_{23}N_4O_3S$: 471.1485; found: 471.1484.

**N-(4-(1-(5-amino-2-methylphenyl)-2-oxo-1,2-dihydrobenzo[h][1,6]naphthyridin-9-yl)phenyl)methanesulfonamide (8c)**

Prepared using the procedure described for **8a**. Compound **8c** was obtained as an orange solid (70 mg; 68% yield). $^1$H NMR (300 MHz, d$^6$-DMSO): δ 9.91 (s, 1H), 9.11 (s, 1H), 8.30 (d, *J* = 9.5 Hz, 1H), 8.12 – 7.97 (m, 2H), 7.53 (d, *J* = 1.8 Hz, 1H), 7.32 (d, *J* = 8.7 Hz, 2H), 7.27 – 7.16 (m, 3H), 6.92 (d, *J* = 9.4 Hz, 1H), 6.79 (dd, *J* = 8.2, 2.3 Hz, 1H), 6.45 (d, *J* = 2.3 Hz, 1H), 5.29 (s, 2H), 3.05 (s, 3H), 1.80 (s, 3H). HRMS (ESI): *m/z* [M + H]$^+$ calc. for $C_{26}H_{23}N_4O_3S$: 471.1485; found 471.1485.

**N-(4-(1-(3-nitrophenyl)-2-oxo-1,2-dihydrobenzo[h][1,6]naphthyridin-9-yl)phenyl)methanesulfonamide (8d)**

Prepared using the procedure described for **8a**. Compound **8d** was obtained as a dark yellow solid (60 mg; 92% yield). $^1$H NMR (300 MHz, d$^6$-DMSO): δ 9.91 (s, 1H), 9.09 (s, 1H), 8.27 (d, *J* = 9.5 Hz, 1H), 8.11 – 7.95 (m, 2H), 7.60 (d, *J* = 1.9 Hz, 1H), 7.36 – 7.27 (m, 3H), 7.23 (d, *J* = 8.8 Hz, 2H), 6.92 – 6.79 (m, 2H), 6.57 (dd, *J* = 6.7, 1.2 Hz, 2H), 5.55 (brs, 2H), 3.04 (s, 3H). HRMS (ESI): *m/z* [M + H]$^+$ calc. for $C_{25}H_{21}N_4O_3S$: 457.1329; found: 457.1328.

**N-(4-(1-(3-amino-4-methoxyphenyl)-2-oxo-1,2-dihydrobenzo[h][1,6]naphthyridin-9-yl)phenyl)methanesulfonamide (8e)**

Prepared using the procedure described for **8a**. Compound **8e** was obtained as an orange solid (85 mg; 78% yield). $^1$H NMR (300 MHz, d$^6$-DMSO): δ 9.93 (s, 1H), 9.07 (s, 1H), 8.26 (d, *J* = 9.5 Hz, 1H), 8.05 (d, *J* = 8.7 Hz, 1H), 7.97 (dd, *J* = 8.7, 1.9 Hz, 1H), 7.37 (d, *J* = 1.9 Hz, 1H), 7.32 – 7.18 (m, 4H), 7.05 (d, *J* = 8.3 Hz, 1H), 6.88 (d, *J* = 9.4 Hz, 1H), 6.67 – 6.53 (m, 2H), 5.11 (s, 2H), 3.91 (s, 3H), 3.04 (s, 3H). HRMS (ESI): *m/z* [M + H]$^+$ calc. for $C_{26}H_{23}N_4O_4S$: 487.1435; found: 487.1433.

**N-(4-(1-(3-amino-4-methylphenyl)-2-oxo-1,2-dihydrobenzo[h][1,6]naphthyridin-8-yl)phenyl)methanesulfonamide (8a')**

Prepared using the procedure described for **8a**. Compound **8a'** was obtained as a bright-yellow solid (80 mg; 71% yield). $^1$H NMR (300 MHz, d$^6$-DMSO): δ 9.96 (s, 1H), 9.10 (s, 1H), 8.26 – 8.23 (m, 2H), 7.84 (d, *J* = 8.7 Hz, 2H), 7.52 (dd, *J* = 9.4, 2.2 Hz, 1H), 7.30 (d, *J* = 8.7 Hz, 2H), 7.15 (m, 2H), 6.84 (d, *J* = 9.4 Hz, 1H), 6.56 (d, *J* = 2.1 Hz, 1H), 6.48 (dd, *J* = 7.8, 2.1 Hz, 1H), 5.20 (s, 2H), 3.04 (s, 3H), 2.21 (s, 3H). HRMS (ESI): *m/z* $[M + H]^+$ calc. for $C_{26}H_{23}N_4O_3S$: 471.1485; found: 471.1483.

**N-(4-(1-(3-amino-5-methylphenyl)-2-oxo-1,2-dihydrobenzo[h][1,6]naphthyridin-8-yl)phenyl)methanesulfonamide (8b')**

Prepared using the procedure described for **8a**. Compound **8b'** was obtained as an orange solid (295 mg; 70% yield). $^1$H NMR (300 MHz, d$^6$-DMSO): δ 9.99 (s, 1H), 9.09 (s, 1H), 8.25 – 8.22 (m, 2H), 7.86 - 7.81 (m, 4H), 7.54 (dd, *J* = 9.4, 2.3 Hz, 1H), 7.30 (d, *J* = 8.5 Hz, 2H), 7.21 (d, *J* = 9.3 Hz, 1H), 6.84 (d, *J* = 9.4 Hz, 1H), 6.61 (s, 1H), 6.34 (s, 2H), 3.04 (s, 3H), 2.22 (s, 3H). HRMS (ESI): *m/z* $[M + H]^+$ calc. for $C_{26}H_{23}N_4O_3S$: 471.1485; found: 471.1483.

**N-(4-(1-(3-aminophenyl)-2-oxo-1,2-dihydrobenzo[h][1,6]naphthyridin-8-yl)phenyl)methanesulfonamide (8d')**

Prepared using the procedure described for **8a**. Compound **8d'** was obtained as a brown solid (140 mg; 85% yield). $^1$H NMR (300 MHz, d$^6$-DMSO): δ 9.09 (s, 1H), 8.26 – 8.22 (m, 2H), 7.79 – 7.77 (m, 2H), 7.50 (dd, *J* = 9.3, 2.2 Hz, 1H), 7.29 – 7.23 (m, 3H), 7.12 (d, *J* = 9.3 Hz, 1H), 6.85 – 6.78 (m, 2H), 6.53 – 6.50 (m, 2H), 5.42 (s, 2H), 2.97 (s, 3H). HRMS (ESI): *m/z* $[M + H]^+$ calc. for $C_{25}H_{21}N_4O_3S$: 457.1329; found: 457.1329.

**Ethyl 4-(3-amino-4-methylphenylamino)-6-(4-(methylsulfonamido)phenyl)quinoline-3-carboxylate (Intermediate 2 – Scheme S3)**

Prepared using the procedure described for **8a**. Compound **INT2** was obtained as a yellow solid (190 mg; 88%). $^1$H NMR (300 MHz, d$^6$-DMSO): δ 10.14 (s, 1H), 9.88 (s, 1H), 8.94 (s, 1H), 8.08 (d, *J* = 2.0 Hz, 1H), 8.04 – 7.96 (m, 1H), 7.92 (d, *J* = 8.7 Hz, 1H), 7.67 – 7.50 (m, 2H), 7.39 (d, *J* = 8.7 Hz, 2H), 7.23 (d, *J* = 8.7 Hz, 2H), 6.97 (d, *J* = 7.9 Hz, 1H), 6.45 (d, *J* = 2.2 Hz, 1H), 6.33 (dd, *J* = 7.9, 2.2 Hz, 1H), 4.19 (q, *J* = 7.1 Hz, 2H), 3.02 (s, 3H), 2.11 (s, 3H), 1.29 (t, *J* = 7.1 Hz, 3H). HRMS (ESI): *m/z* [M + H]$^+$ calc. for $C_{26}H_{27}N_4O_4S$: 491.1753; found: 491.1748.

**N-(4-(1-(3-amino-4-methylphenyl)-2-oxo-1,2-dihydrobenzo[h][1,6]naphthyridin-9-yl)phenyl)butane-1-sulfonamide (8f – precursor of 15)**

Prepared using the procedure described for **8a**. Compound **8f** was obtained as a bright-yellow solid (163 mg; 91% yield). $^1$H NMR (300 MHz, d$^6$-DMSO): δ 9.92 (s, 1H), 9.06 (s, 1H), 8.25 (d, *J* = 9.5 Hz, 1H), 8.04 (d, *J* = 8.6 Hz, 1H), 7.96 (dd, *J* = 8.7, 1.9 Hz, 1H), 7.70 – 7.48 (m, 2H), 7.39 (d, *J* = 1.9 Hz, 1H), 7.30 – 7.13 (m, 5H), 6.87 (d, *J* = 9.4 Hz, 1H), 6.61 (d, *J* = 2.1 Hz, 1H), 6.51 (dd, *J* = 7.8, 2.1 Hz, 1H), 3.12 (t, *J* = 7.4 Hz, 2H), 2.25 (s, 3H), 1.76 – 1.58 (m, 2H), 1.47 – 1.29 (m, 2H), 0.85 (t, *J* = 7.3 Hz, 3H). HRMS (ESI): *m/z* [M + H]$^+$ calc. for $C_{29}H_{29}N_4O_3S$: 513.1955; found: 513.1954.

**N-(3-(1-(3-amino-4-methylphenyl)-2-oxo-1,2-dihydrobenzo[h][1,6]naphthyridin-8-yl)phenyl)methanesulfonamide (8g – precursor of 16)**

Prepared using the procedure described for **8a**. Compound **8g** was obtained as a bright-yellow solid (75 mg; 94% yield). HRMS (ESI): *m/z* [M + H]$^+$ calc. for $C_{26}H_{23}N_4O_3S$: 471.1484; found: 471.1485.

**Methyl 4-(1-(3-amino-4-methylphenyl)-2-oxo-1,2-dihydrobenzo[h][1,6]naphthyridin-9-yl)phenylcarbamate (8h – precursor of 17)**

Prepared using the procedure described for **8a**. Compound **8h** was obtained as a bright-yellow solid (147 mg; 100% yield). $^1$H NMR (300 MHz, d$^6$-DMSO): δ 9.77 (s, 1H), 9.06 (s, 1H), 8.25 (d, *J* = 9.4 Hz, 1H), 8.08 – 7.91 (m, 2H), 7.51 (d, *J* = 8.3 Hz, 2H), 7.43 (d, *J* = 1.8 Hz, 1H), 7.22 – 7.18 (m, 3H), 6.87 (d, *J* = 9.4 Hz, 1H), 6.61 (d, *J* = 2.1 Hz, 1H), 6.51 (dd, *J* = 7.8, 2.1 Hz, 1H), 5.25 (brs, 2H), 3.70 (s, 3H), 2.27 (s, 3H). HRMS (ESI): *m/z* $[M + H]^+$ calc. for $C_{27}H_{23}N_4O_3$: 451.1765; found: 451.1763.

**1-(3-amino-4-methylphenyl)-9-(pyridin-4-yl)benzo[h][1,6]naphthyridin-2(1H)-one (8i – precursor of 18)**

Prepared using the procedure described for **8a**. During phase separation, the aqueous phase (pH~5) was adjusted to pH 7 with $NaHCO_3$. Compound **8i** was obtained as an orange solid (75 mg; 100% yield). $^1$H NMR (300 MHz, d$^6$-DMSO): δ 9.14 (s, 1H), 8.64 – 8.55 (m, 2H), 8.28 (d, *J* = 9.5 Hz, 1H), 8.11 (s, 2H), 7.51 (s, 1H), 7.31 – 7.23 (m, 2H), 7.20 (d, *J* = 8.2 Hz, 1H), 6.90 (d, *J* = 9.4 Hz, 1H), 6.66 (d, *J* = 2.1 Hz, 1H), 6.49 (dd, *J* = 7.8, 2.1 Hz, 1H), 5.33 (s, 2H), 2.25 (s, 3H). HRMS (ESI): *m/z* $[M + H]^+$ calc. for $C_{24}H_{19}N_4O$: 379.1553; found: 379.1552.

**5-(1-(3-amino-4-methylphenyl)-2-oxo-1,2-dihydrobenzo[h][1,6]naphthyridin-9-yl)picolinonitrile (8j – precursor of 19)**

Prepared using the procedure described for **8a**. Compound **8j** was obtained as an orange solid (79 mg; 97% yield). HRMS (ESI): *m/z* $[M + H]^+$ calc. for $C_{25}H_{18}N_5O$: 404.1506; found: 404.1507.

**1-(3-amino-4-methylphenyl)-9-(6-(trifluoromethyl)pyridin-3-yl)benzo[h][1,6]naphthyridin-2(1H)-one (8k – precursor of 20)**

Prepared using the procedure described for **8a**. Compound **8k** was obtained as an orange solid (99 mg; 100% yield). $^1$H NMR (300 MHz, d$^6$-DMSO): δ 9.15 (s, 1H), 8.67 (d, *J* = 2.1 Hz, 1H), 8.29 (d, *J* = 9.5 Hz, 1H), 8.13 (brs, 2H), 7.95 (m, 1H), 7.88 (m, 1H), 7.33 (s, 1H), 7.19 (d, *J* = 7.8 Hz, 1H), 6.92 (d, *J* = 9.4 Hz, 1H), 6.64 (d, *J* = 2.1 Hz, 1H), 6.51 (dd, *J* = 7.7, 2.1 Hz, 1H), 5.33 (s, 2H), 2.21 (s, 3H). HRMS (ESI): *m/z* $[M + H]^+$ calc. for $C_{25}H_{18}F_3N_4O$: 447.1427; found: 447.1426.

**1-(3-amino-4-methylphenyl)-9-(piperidin-1-yl)benzo[h][1,6]naphthyridin-2(1H)-one (8l – precursor of 21)**

Prepared using the procedure described for **8a**. Compound **8l** was obtained as a yellow solid (134 mg; 85% yield). HRMS (ESI): *m/z* $[M + H]^+$ calc. for $C_{24}H_{25}N_4O$: 385.2023; found: 385.2025.

**1-(3-amino-4-methylphenyl)-9-morpholinobenzo[h][1,6]naphthyridin-2(1H)-one (8m – precursor of 22)**

Prepared using the procedure described for **8a**. Compound **8m** was obtained as an orange solid (141 mg; 95% yield).HRMS (ESI): *m/z* $[M + H]^+$ calc. for $C_{23}H_{23}N_4O_2$: 387.1816; found: 387.1817.

**Synthesis of 1-(3-amino-4-methylphenyl)-9-(4-(methylsulfonyl)piperazin-1-yl)benzo[h][1,6]naphthyridin-2(1H)-one (8n)**

Prepared using the procedure described for **8a**. Compound **8n** was obtained as a yellow solid (285 mg; 88% yield). $^1$H NMR (300 MHz, $CDCl_3$): δ 8.79 (s, 1H), 7.94 (dd, *J* = 16.9, 9.3 Hz,

2H), 7.33 (dd, $J$ = 9.2, 2.6 Hz, 1H), 7.27 (m, 1H), 6.90 (d, $J$ = 9.4 Hz, 1H), 6.78 (d, $J$ = 2.6 Hz, 1H), 6.68 (d, $J$ = 6.4 Hz, 2H), 3.83 (s, 2H), 3.26 – 3.22 (m, 4H), 2.99 – 2.96 (m, 4H), 2.82 (s, 3H), 2.26 (s, 3H). HRMS (ESI): *m/z* $[M + H]^+$ calc. for $C_{24}H_{26}N_5O_3S$: 464.1751; found: 464.1748.

**1-(3-amino-4-methylphenyl)-9-(4-(dimethylamino)piperidin-1-yl)benzo[h][1,6]naphthyridin-2(1H)-one (8o – precursor of 23)**

Prepared using the procedure described for **8a**. The reaction required 12 eq. of Fe and $NH_4Cl$ and 5h reaction time at 80 ºC. During phase separation, the aqueous phase (pH~5) was adjusted to pH 8 with $NaHCO_3$. Compound **8o** was obtained as a yellow solid (85 mg; 46% yield). $^1H$ NMR (300 MHz, $d^6$-DMSO): δ 8.81 (s, 1H), 8.16 (d, $J$ = 9.4 Hz, 1H), 7.79 (d, $J$ = 9.1 Hz, 1H), 7.46 (dd, $J$ = 9.4, 2.4 Hz, 1H), 7.13 (d, $J$ = 7.8 Hz, 1H), 6.79 (d, $J$ = 9.4, 1H), 6.71 (brs, 1H), 6.57 (d, $J$ = 1.8 Hz, 1H), 6.49 – 6.37 (m, 1H), 5.22 (s, 2H), 3.34 – 3.30 (m, 2H), 2.50 (s, 3H), 2.42 – 2.36 (m, 2H), 2.16 (s, 6H), 2.16 (m, 1H), 1.70 – 1.66 (m, 2H), 1.30 – 1.22 (m, 2H). HRMS (ESI): *m/z* $[M + H]^+$ calc. for $C_{26}H_{30}N_5O$: 428.2445; found: 428.2442.

**1-(3-aminophenyl)-8-(4-(methylsulfonyl)piperazin-1-yl)benzo[h][1,6]naphthyridin-2(1H)-one (8d'n' – precursor of JS27)**

Prepared using the procedure described for **8a**. Compound **8d'n'** was obtained as an orange solid (225 mg; 71% yield). $^1H$ NMR (300 MHz, $CDCl_3$): δ 8.92 (s, 1H), 8.14 (d, $J$ = 9.4 Hz, 1H), 7.28 – 7.21 (m, 2H), 7.03 – 6.96 (m, 1H), 6.86 (d, $J$ = 9.8 Hz, 1H), 6.77 – 6.68 (m, 2H), 6.46 (brs, 2H), 5.43 (s, 2H), 3.43 (m, 4H,) 3.20 (m, 4H), 2.89 (s, 3H). HRMS (ESI): *m/z* $[M + H]^+$ calc. for $C_{23}H_{24}N_5O_3S$: 450.1594; found: 450.1600

*General Procedure H: Nitro to amine reduction with $SnCl_2$*

**1-(3-amino-4-methylphenyl)-9-bromobenzo[h][1,6]naphthyridin-2(1H)-one (Intermediate 3 – Scheme S4)**

Intermediate **6a** (1.03 g; 2.511 mmol) was suspended in EtOAc (50 mLl) and $SnCl_2$ (2.86 g; 15.06 mmol; 6 equiv.) was added. The mixture was heated to 85 ºC and after 2 h, the reaction cooled to rt and saturated $NaHCO_3$ aq. was added. The phases were separated and the aqueous phase further extracted with ethyl acetate (2x). The combined organics were washed with brine, dried over MgSO4, taken to dryness to afford **INT3** as a brownish solid (650 mg; 68% yield).

$^1$H NMR (300 MHz, d$^6$-DMSO): δ 9.11 (s, 1H), 8.25 (d, $J$ = 9.4 Hz, 1H), 7.91 (d, $J$ = 8.9 Hz, 1H), 7.78 (dd, $J$ = 8.9, 2.1 Hz, 1H), 7.18 (d, $J$ = 7.8 Hz, 1H), 6.99 (d, $J$ = 2.1 Hz, 1H), 6.90 (d, $J$ = 9.4 Hz, 1H), 6.53 (d, $J$ = 2.1 Hz, 1H), 6.45 (dd, $J$ = 7.7, 2.1 Hz, 1H), 5.24 (s, 2H), 2.22 (s, 3H). HRMS (ESI): *m/z* $[M + H]^+$ calc. for $C_{19}H_{15}BrN_3O$: 380.0393; found: 380.0390.

*General Procedure I: Acylation*

**N-(2-methyl-5-(9-(4-(methylsulfonamido)phenyl)-2-oxobenzo[h][1,6]naphthyridin-1(2H)-yl)phenyl)acrylamide (9a; BMX-IN-1)**

A stirred solution of **8a** (90 mg; 0.191 mmol) in dry THF (20 mL) was cooled in an ice-bath to -10ºC for 20 min. DIPEA (133 µL; 0.765 mmol; 4 equiv.) was added and the mixture stirred for 10 min at a temperature < 4 ºC. After 10 min, acryloyl chloride was added and the mixture further stirred at -10ºC for 10 min. and then 1h at rt. THF was then evaporated and the crude redissolved in EtOAc and washed three times with $NaHCO_3$ (4%). The organics were dried over $MgSO_4$ and taken to dryness. The crude was applied in a silica column and eluted with a gradient from 100:0 to 96:4 in DCM:MeOH. The desired fractions were collected and taken to

dryness to afford the title compound as a white solid. (20 mg; 20% yield). $^1$H NMR (300 MHz, d$^6$-DMSO): δ 9.85 (s, 1H), 9.78 (s, 1H), 9.11 (s, 1H), 8.30 (d, *J* = 9.4 Hz, 1H), 8.08 (d, *J* = 8.7 Hz, 1H), 7.98 (dd, *J* = 8.7, 1.9 Hz, 1H), 7.69 (s, 1H), 7.52 (d, *J* = 8.1 Hz, 1H), 7.23-7.20 (m, 6H), 6.91 (d, *J* = 9.4 Hz, 1H), 6.57 (dd, *J* = 17.2, 10.2 Hz, 1H), 6.19 (d, *J* = 17.2 Hz, 1H), 5.74 (d, *J* = 10.2 Hz, 1H), 3.01 (s, 3H), 2.42 (s, 3H). HRMS (ESI): *m/z* [M + H]$^+$ calc. for $C_{29}H_{25}N_4O_4S$: 525.1591; found: 525.1586. HPLC Purity: 98.9%.

**NOTE**: BMX-IN-1 was also acquired from Calbiochem and the commercial compound was used for *in vitro* experiments (including BMX and BTK $IC_{50}$ determination, kinetic measurements, target engagement and cellular assays).

**N-(3-methyl-5-(9-(4-(methylsulfonamido)phenyl)-2-oxobenzo[h][1,6]naphthyridin-1(2H)-yl)phenyl)acrylamide (9b)**

Prepared using the procedure described for **9a**. Compound **9b** was obtained as a light-yellow solid (8 mg; 10% yield). $^1$H NMR (300 MHz, d$^6$-DMSO): δ 10.42 (s, 1H), 9.92 (s, 1H), 9.12 (s, 1H), 8.31 (d, *J* = 9.5 Hz, 1H), 8.08 (d, *J* = 8.8 Hz, 1H), 8.00 (dd, *J* = 8.8, 1.9 Hz, 1H), 7.78 (s, 1H), 7.57 (brs, 1H), 7.44 (d, *J* = 1.9 Hz, 1H), 7.20 (brs, 4H), 7.09 (s, 1H), 6.92 (d, *J* = 9.4 Hz, 1H), 6.39 (dd, *J* = 17.0, 10.0 Hz, 1H), 6.22 (dd, *J* = 17.0, 2.2 Hz, 1H), 5.73 (dd, *J* = 9.9, 2.1 Hz, 1H), 3.03 (s, 3H), 2.36 (s, 3H). HRMS (ESI): *m/z* [M + H]$^+$ calc. for $C_{29}H_{25}N_4O_4S$: 525.1591; found: 525.1576. HPLC Purity: 93.1%.

**N-(4-methyl-3-(9-(4-(methylsulfonamido)phenyl)-2-oxobenzo[h][1,6]naphthyridin-1(2H)-yl)phenyl)acrylamide (9c)**

Prepared using the procedure described for **9a**. Compound **9c** was obtained as a light-yellow solid (40 mg; 55% yield). $^1$H NMR (300 MHz, d$^6$-DMSO): δ 10.40 (s, 1H), 9.91 (brs, 1H), 9.14 (s, 1H), 8.35 (d, *J* = 9.5 Hz, 1H), 8.10 (d, *J* = 8.7 Hz, 1H), 8.01 (dd, *J* = 8.7, 1.9 Hz, 1H), 7.83

(dd, $J$ = 8.4, 2.2 Hz, 1H), 7.72 (d, $J$ = 2.1 Hz, 1H), 7.55 (d, $J$ = 8.4 Hz, 1H), 7.32 (d, $J$ = 1.8 Hz, 1H), 7.19 (m, 4H), 6.96 (d, $J$ = 9.4 Hz, 1H), 6.39 (dd, $J$ = 17.0, 10.0 Hz, 1H), 6.22 (dd, $J$ = 17.0, 2.2 Hz, 1H), 5.74 (dd, $J$ = 10.0, 2.2 Hz, 1H), 3.03 (s, 3H), 1.91 (s, 3H). HRMS (ESI): *m/z* $[M + H]^+$ calc. for $C_{29}H_{25}N_4O_4S$: 525.1591; found: 525.1587. HPLC Purity: 99.5%.

**N-(3-(9-(4-(methylsulfonamido)phenyl)-2-oxobenzo[h][1,6]naphthyridin-1(2H)-yl)phenyl)acrylamide (9d)**

Prepared using the procedure described for **9a**. Compound **9d** was obtained as a light-yellow solid (27 mg; 50% yield). $^1$H NMR (300 MHz, d$^6$-DMSO): δ 10.50 (s, 1H), 9.91 (s, 1H), 9.12 (s, 1H), 8.31 (d, $J$ = 9.4 Hz, 1H), 8.08 (d, $J$ = 8.7 Hz, 1H), 8.03 – 7.91 (m, 2H), 7.83 (brs, 1H), 7.63 (t, $J$ = 8.1 Hz, 1H), 7.34 (brs, 1H), 7.18 (m, 5H), 6.92 (d, $J$ = 9.4 Hz, 1H), 6.42 (dd, $J$ = 16.9, 10.0 Hz, 1H), 6.26 (dd, $J$ = 16.9, 2.2 Hz, 1H), 5.77 (dd, $J$ = 9.7, 1.9 Hz, 1H), 3.03 (s, 3H). HRMS (ESI): *m/z* $[M + H]^+$ calc. for $C_{28}H_{23}N_4O_4S$: 511.1435; found: 511.1433. HPLC Purity: 98.0%.

**N-(2-methoxy-5-(9-(4-(methylsulfonamido)phenyl)-2-oxobenzo[h][1,6]naphthyridin-1(2H)-yl)phenyl)acrylamide (9e)**

Prepared using the procedure described for **9a**. Compound **9e** was obtained as a light-yellow solid (38 mg; 43% yield). $^1$H NMR (300 MHz, d$^6$-DMSO): δ 9.73 (s, 1H), 9.10 (s, 1H), 8.29 (d, $J$ = 9.5 Hz, 1H), 8.19 – 8.10 (m, 1H), 8.07 (d, $J$ = 8.6 Hz, 1H), 7.97 (d, $J$ = 8.7 Hz, 1H), 7.36 – 7.20 (m, 8H), 6.91 (d, $J$ = 9.4 Hz, 1H), 6.74 (dd, $J$ = 17.0, 10.2 Hz, 1H), 6.13 (dd, $J$ = 17.0, 2.1 Hz, 1H), 5.68 (dd, $J$ = 10.3, 2.1 Hz, 1H), 4.00 (s, 3H), 3.01 (s, 3H). HRMS (ESI): *m/z* $[M + H]^+$ calc. for $C_{29}H_{25}N_4O_5S$: 541.1540; found 541.1542. HPLC Purity: 97.0%.

**N-(2-methyl-5-(9-(4-(methylsulfonamido)phenyl)-2-oxobenzo[h][1,6]naphthyridin-1(2H)-yl)phenyl)but-2-enamide (10)**

Prepared using the procedure described for **9a**. Compound **10** was obtained as a pale-yellow solid (30 mg; 41%). $^{1}$H NMR (300 MHz, d$^{6}$-DMSO): δ 9.86 (s, 1H), 9.55 (s, 1H), 9.10 (s, 1H), 8.29 (d, *J* = 9.5 Hz, 1H), 8.02 (q, *J* = 8.6 Hz 2H), 7.69 (m, 1H), 7.49 (d, *J* = 8.1 Hz, 1H), 7.21 (brs, 6H), 6.91 (d, *J* = 9.4 Hz, 1H), 6.73 (dd, *J* = 15.1, 7.2 Hz, 1H), 6.27 (d, *J* = 15.3 Hz, 1H), 3.02 (s, 3H), 2.41 (s, 3H), 1.83 (s, 3H). HRMS (ESI): *m/z* $[M + H]^+$ calc. for $C_{30}H_{27}N_4O_4S$: 539.1748; found: 539.1746. HPLC Purity: 99.3%.

**3-methyl-N-(2-methyl-5-(9-(4-(methylsulfonamido)phenyl)-2-oxobenzo[h][1,6]naphthyridin-1(2H)-yl)phenyl)but-2-enamide (11)**

Prepared using the procedure described for **9a**. Compound **11** was obtained as a pale-yellow solid (20 mg; 32%). $^{1}$H NMR (300 MHz, d$^{6}$-DMSO): δ 9.39 (s, 1H), 9.10 (s, 1H), 8.29 (d, J = 9.5 Hz, 1H), 8.08 – 7.96 (m, 2H), 7.72 (brs, 1H), 7.47 (d, J = 8.1 Hz, 1H), 7.16 (m, 6H), 6.91 (d, J = 9.4 Hz, 1H), 6.03 (s, 1H), 2.98 (s, 3H), 2.40 (s, 3H), 2.04 (s, 3H), 1.84 (s, 3H). HRMS (ESI): *m/z* $[M + H]^+$ calc. for $C_{31}H_{29}N_4O_4S$: 553.1904; found: 553.1909. HPLC Purity: 99.3%.

**N-(5-(9-(4-(butylsulfonamido)phenyl)-2-oxobenzo[h][1,6]naphthyridin-1(2H)-yl)-2-methylphenyl)acrylamide (14)**

Prepared using the procedure described for **9a**. Compound **14** was obtained as a pale-yellow solid (33 mg; 20%). $^{1}$H NMR (300 MHz, d$^{6}$-DMSO): δ 9.92 (s, 1H), 9.81 (s, 1H), 9.11 (s, 1H), 8.30 (d, *J* = 9.4 Hz, 1H), 8.07 (d, *J* = 8.6 Hz, 1H), 7.98 (d, *J* = 9.0 Hz, 1H), 7.69 (s, 1H), 7.50 (d, *J* = 8.1 Hz, 1H), 7.19 (brs, 6H), 6.91 (d, *J* = 9.5 Hz, 1H), 6.57 (dd, *J* = 17.1, 9.4 Hz, 1H), 6.19 (d, *J* = 17.0 Hz, 1H), 5.74 (d, *J* = 10.1 Hz, 1H), 3.09 (t, *J* = 7.8 Hz, 2H), 2.40 (s, 3H), 1.74

– 1.54 (m, 2H), 1.35 (q, *J* = 7.4 Hz, 2H), 0.83 (t, *J* = 7.3 Hz, 3H). HRMS (ESI): *m/z* $[M + H]^+$ calc. for $C_{32}H_{31}N_4O_4S$: 567.2061; found 567.2060. HPLC Purity: 95.2%.

**N-(2-methyl-5-(9-(3-(methylsulfonamido)phenyl)-2-oxobenzo[h][1,6]naphthyridin-1(2H)-yl)phenyl)acrylamide (15)**

Prepared using the procedure described for **9a**. Compound **15** was purified by semi-prep HPLC, with an XBridge BEH C18 OBD column (130 Å, 5 µm, 10 mm x 100 mm) with a gradient 25:75 until 50:50 with a mixture (95:5 ACN:$NaHCO_3$ 10 mM) : ($NaHCO_3$ 10 mM). The title compound was obtained as a white solid (8 mg; 4%). $^1H$ NMR (300 MHz, $CDCl_3$): δ 8.98 (s, 1H), 8.68 (s, 1H), 8.17 (d, *J* = 8.7 Hz, 1H), 8.07 – 7.94 (m, 2H), 7.88 (dd, *J* = 8.7, 1.9 Hz, 1H), 7.54 – 7.32 (m, 5H), 7.25 (m, 1H), 7.09 (dd, *J* = 8.1, 2.2 Hz, 1H), 6.98 – 6.84 (m, 2H), 6.56 (dd, *J* = 16.9, 1.3 Hz, 1H), 6.36 (dd, *J* = 16.9, 10.2 Hz, 1H), 5.85 (dd, *J* = 10.2, 1.3 Hz, 1H), 2.98 (s, 3H), 2.34 (s, 3H). HRMS (ESI): *m/z* $[M + H]^+$ calc. for $C_{29}H_{25}N_4O_4S$: 525.1591; found 525.1594. HPLC Purity: 94.3%.

**Methyl 4-(1-(3-acrylamido-4-methylphenyl)-2-oxo-1,2-dihydrobenzo[h][1,6]naphthyridin-9-yl)phenylcarbamate (16)**

Prepared using the procedure described for **9a**. Compound **16** was obtained as a pale-yellow solid (55 mg; 36%). $^1H$ NMR (300 MHz, $d^6$-DMSO): δ 9.75 (s, 2H), 9.09 (s, 1H), 8.29 (d, *J* = 9.5 Hz, 1H), 8.15 – 7.90 (m, 2H), 7.69 (d, *J* = 2.0 Hz, 1H), 7.53 - 7.46 (m, 3H), 7.32 – 7.08 (m, 4H), 6.90 (d, *J* = 9.4 Hz, 1H), 6.58 (dd, *J* = 17.0, 10.1 Hz, 1H), 6.19 (dd, *J* = 17.0, 2.0 Hz, 1H), 5.73 (d, *J* = 10.4 Hz, 1H), 3.68 (s, 3H), 2.44 (s, 3H). HRMS (ESI): *m/z* $[M + H]^+$ calc. for $C_{30}H_{25}N_4O_4$: 505.1870; found 505.1869. HPLC Purity: 97.8%.

**N-(2-methyl-5-(2-oxo-9-(pyridin-4-yl)benzo[h][1,6]naphthyridin-1(2H)-yl)phenyl)acrylamide (17)**

Prepared using the procedure described for **9a**. Compound **17** was obtained as a yellow solid (29 mg; 20%). $^1$H NMR (300 MHz, d$^6$-DMSO): δ 9.76 (s, 1H), 9.17 (s, 1H), 8.56 (s, 2H), 8.32 (d, *J* = 9.5 Hz, 1H), 8.16-8.12 (m, 2H), 7.73 (s, 1H), 7.52 (d, *J* = 8.1 Hz, 1H), 7.38 (s, 1H), 7.26-7.20 (m, 3H), 6.94 (d, *J* = 9.6 Hz, 1H), 6.57 (dd, *J* = 16.5, 9.6 Hz, 1H), 6.20 (d, *J* = 16.5 Hz, 1H), 5.74 (d, *J* = 9.3 Hz, 1H), 2.43 (s, 3H). HRMS (ESI): *m/z* $[M + H]^+$ calc. for $C_{27}H_{21}N_4O_2$: 433.1659; found: 433.1656. HPLC Purity: 95.8%.

**N-(5-(9-(6-cyanopyridin-3-yl)-2-oxobenzo[h][1,6]naphthyridin-1(2H)-yl)-2-methylphenyl)acrylamide (18)**

Prepared using the procedure described for **9a**. Compound **18** was obtained as a light-yellow solid (14 mg; 17%). $^1$H NMR (300 MHz, d$^6$-DMSO): δ 9.75 (s, 1H), 9.19 (s, 1H), 8.61 (dd, *J* = 2.3, 0.8 Hz, 1H), 8.32 (d, *J* = 9.5 Hz, 1H), 8.16 - 8.13 (m, 2H), 8.06 (dd, *J* = 8.2, 0.8 Hz, 1H), 7.85 (dd, *J* = 8.2, 2.3 Hz, 1H), 7.67 (d, *J* = 2.2 Hz, 1H), 7.51 (d, *J* = 8.1 Hz, 1H), 7.25 – 7.22 (m, 2H), 6.94 (d, *J* = 9.4 Hz, 1H), 6.55 (dd, *J* = 17.0, 10.1 Hz, 1H), 6.17 (dd, *J* = 17.0, 2.0 Hz, 1H), 5.73 (dd, *J* = 10.1, 2.0 Hz, 1H), 2.42 (s, 3H). HRMS (ESI): *m/z* $[M + H]^+$ calc. for $C_{28}H_{20}N_5O_2$: 458.1612; found: 458.1610. HPLC Purity: 95.3%.

**N-(2-methyl-5-(2-oxo-9-(6-(trifluoromethyl)pyridin-3-yl)benzo[h][1,6]naphthyridin-1(2H)-yl)phenyl)acrylamide (19)**

Prepared using the procedure described for **9a**. Compound **19** was obtained as a yellow solid (20 mg; 18%). $^1$H NMR (300 MHz, d$^6$-DMSO): δ 9.77 (s, 1H), 9.18 (s, 1H), 8.66 (s, 1H), 8.32 (d, *J* = 9.4 Hz, 1H), 8.17-8.14 (m, 2H), 7.87 (s, 2H), 7.67 (d, *J* = 2.2 Hz, 1H), 7.51 (d, *J* = 8.0 Hz, 1H), 7.25-7.22 (m, 2H), 6.94 (d, *J* = 9.4 Hz, 1H), 6.55 (dd, *J* = 17.1, 10.2 Hz, 1H), 6.17

(dd, $J$ = 17.1, 2.2 Hz, 1H), 5.73 (d, $J$ = 10.2 Hz, 1H), 2.39 (s, 3H). HRMS (ESI): *m/z* $[M + H]^+$ calc. for $C_{28}H_{20}F_3N_4O_2$: 501.1533; found: 501.1534. HPLC Purity: 98.3%.

**N-(2-methyl-5-(2-oxo-9-(piperidin-1-yl)benzo[h][1,6]naphthyridin-1(2H)-yl)phenyl)acrylamide (20)**

Prepared using the procedure described for **9a**. Compound **20** was further purified by preparative TLC eluting with DCM:MeOH (96:4) to afford the title compound as a yellow solid (70 mg; 34%). $^1H$ NMR (300 MHz, $CDCl_3$): δ 8.76 (s, 1H), 8.26 (s, 1H), 8.03 – 7.79 (m, 3H), 7.38 – 7.27 (m, 2H), 6.97 - 6.90 (m, 2H), 6.62 (d, $J$ = 2.6 Hz, 1H), 6.33 – 6.26 (m, 2H), 5.67 (t, $J$ = 5.9 Hz, 1H), 2.74 (m, 4H), 2.29 (s, 3H), 1.48 (m, 6H). HRMS (ESI): *m/z* $[M + H]^+$ calc. for $C_{27}H_{27}N_4O_2$: 439.2129; found: 439.2127. HPLC Purity: 98.2%.

**N-(2-methyl-5-(9-morpholino-2-oxobenzo[h][1,6]naphthyridin-1(2H)-yl)phenyl)acrylamide (21)**

Prepared using the procedure described for **9a**. Compound **21** was further purified by preparative TLC eluting with DCM:MeOH (95:5) to afford the title compound as a light-yellow solid (36 mg; 29%). $^1H$ NMR (300 MHz, $CDCl_3$): δ 8.80 (s, 1H), 8.17 (s, 1H), 7.97 (t, $J$ = 9.4 Hz, 2H), 7.87 (s, 1H), 7.38 – 7.28 (m, 2H), 6.99 - 6.92 (m, 2H), 6.64 (d, $J$ = 2.6 Hz, 1H), 6.41 – 6.26 (m, 2H), 5.70 (m, 1H), 3.68 (t, $J$ = 4.8 Hz, 4H), 2.88 – 2.62 (m, 4H), 2.28 (s, 3H). HRMS (ESI): *m/z* $[M + H]^+$ calc. for $C_{26}H_{25}N_4O_3$: 441.1921; found: 441.1920. HPLC Purity: 97.0%.

**N-(2-methyl-5-(9-(4-(methylsulfonyl)piperazin-1-yl)-2-oxobenzo[h][1,6]naphthyridin-1(2H)-yl)phenyl)acrylamide (22)**

Prepared using the procedure described for **9a**. Compound **22** was obtained as a yellow solid (45 mg; 25%). $^1H$ NMR (300 MHz, $CDCl_3$): δ 8.83 (s, 1H), 8.11 – 7.95 (m, 4H), 7.35 – 7.30

(m, 2H), 6.96 (d, $J$ = 9.3 Hz, 2H), 6.68 (d, $J$ = 2.6 Hz, 1H), 6.34 – 6.30 (m, 2H), 5.71 (m, 1H), 3.29 – 3.02 (m, 4H), 2.97 – 2.93 (m, 4H), 2.76 (s, 3H), 2.29 (s, 3H). HRMS (ESI): *m/z* [M + H]$^+$ calc. for $C_{27}H_{28}N_5O_4S$: 518.1857; found: 518.1859. HPLC Purity: 96.7%.

**N-(5-(9-(4-(dimethylamino)piperidin-1-yl)-2-oxobenzo[h][1,6]naphthyridin-1(2H)-yl)-2-methylphenyl)acrylamide (23)**

Prepared using the procedure described for **9a**. Compound **23** was obtained as a light-yellow solid (9 mg; 10%). $^1$H NMR (300 MHz, $CDCl_3$): δ 8.77 (s, 1H), 8.25 (s, 1H), 8.09 – 7.84 (m, 3H), 7.42 – 7.27 (m, 2H), 6.96 – 6.91 (m, 2H), 6.64 (d, $J$ = 2.6 Hz, 1H), 6.40 – 6.23 (m, 2H), 5.75 – 5.58 (m, 1H), 3.30 (t, $J$ = 12.1 Hz, 2H), 2.43 – 2.29 (m, 3H), 2.29 (s, 3H), 2.26 (s, 6H), 1.70 - 1.66 (m, 2H), 1.44 – 1.38 (m, 2H). HRMS (ESI): *m/z* [M + H]$^+$ calc. for $C_{29}H_{32}N_5O_2$: 482.2551; found: 482.2551. HPLC Purity: 99.5%.

**N-(2-methyl-5-(8-(4-(methylsulfonamido)phenyl)-2-oxobenzo[h][1,6]naphthyridin-1(2H)-yl)phenyl)acrylamide (JS24)**

Prepared using the procedure described for **9a**. Compound **JS24** was obtained as a yellow solid (35 mg; 43%). $^1$H NMR (300 MHz, $d^6$-DMSO): δ 9.98 (s, 1H), 9.68 (s, 1H), 9.13 (s, 1H), 8.37 – 8.23 (m, 2H), 7.83 (d, $J$ = 8.6 Hz, 2H), 7.67 (s, 1H), 7.58 – 7.41 (m, 2H), 7.29 (d, $J$ = 8.7 Hz, 2H), 7.19 (dd, $J$ = 8.0, 2.2 Hz, 1H), 6.98 (d, $J$ = 9.3 Hz, 1H), 6.88 (d, $J$ = 9.4 Hz, 1H), 6.56 (dd, $J$ = 17.1, 10.1 Hz, 1H), 6.19 (dd, $J$ = 17.0, 2.1 Hz, 1H), 5.74 (d, $J$ = 10.1 Hz, 1H), 3.03 (s, 3H), 2.40 (s, 3H). HRMS (ESI): *m/z* [M + H]$^+$ calc. for $C_{29}H_{25}N_4O_4S$: 525.1591; found 525.1591. HPLC Purity: 95.1%.

## $^{1}$H NMR of JS24 in d[6]-DMSO:

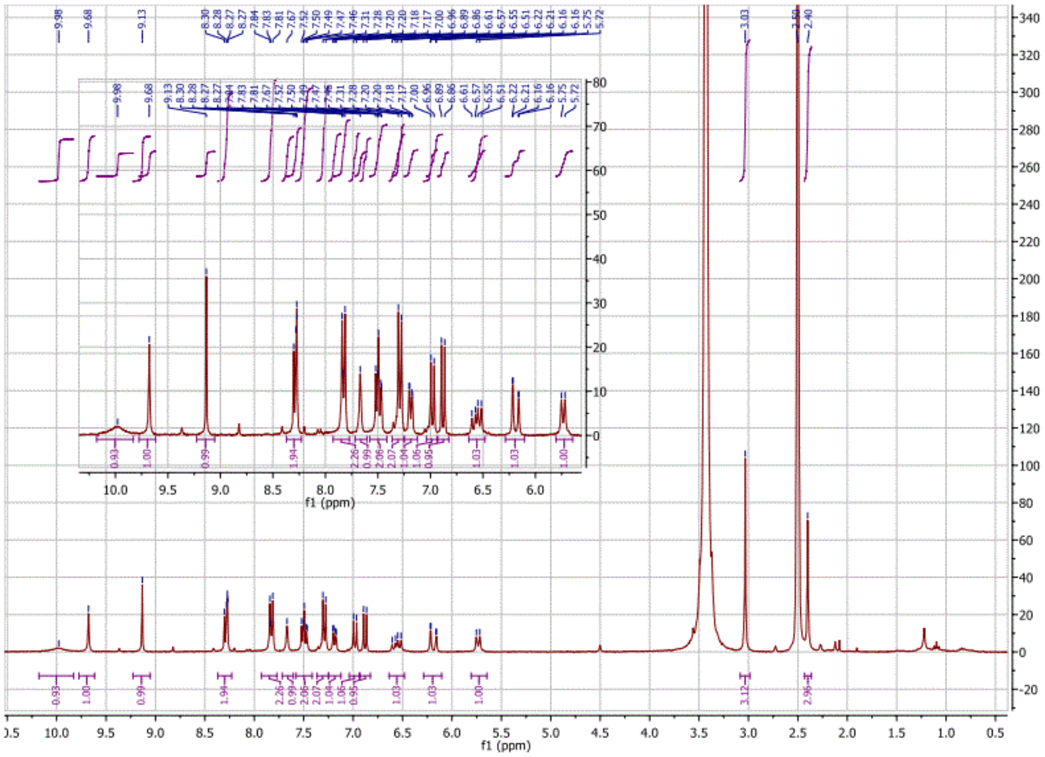

## HPLC trace of JS24:

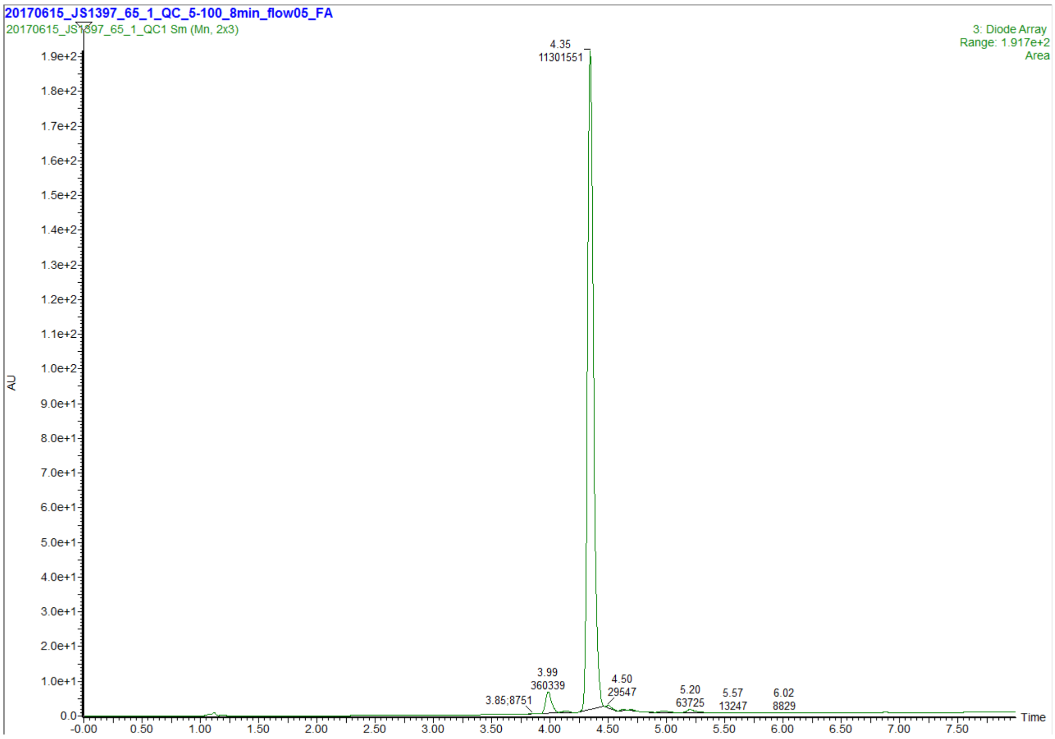

**N-(3-methyl-5-(8-(4-(methylsulfonamido)phenyl)-2-oxobenzo[h][1,6]naphthyridin-1(2H)-yl)phenyl)acrylamide (JS25)**

Prepared using the procedure described for **9a**. Compound **JS25** was further purified by preparative TLC eluting with DCM:MeOH (97:3) to afford the title compound as a yellow solid (10 mg; 9%). $^{1}H$ NMR (300 MHz, $d^6$-DMSO): δ 10.39 (s, 1H), 9.98 (brs, 1H), 9.14 (s, 1H), 8.31 – 8.28 (m, 2H), 7.84 (d, *J* = 8.4 Hz, 2H), 7.74 (s, 1H), 7.59 – 7.54 (m, 2H), 7.28 (d, *J* = 8.3 Hz, 2H), 7.01 – 6.96 (m, 2H), 6.89 (d, *J* = 9.4 Hz, 1H), 6.43 (dd, *J* = 17.0, 10.0 Hz, 1H), 6.23 (dd, *J* = 17.0, 2.1 Hz, 1H), 5.76 (dd, *J* = 9.9, 2.1 Hz, 1H), 3.03 (s, 3H), 2.37 (s, 3H). HRMS (ESI): *m/z* $[M + H]^+$ calc. for $C_{29}H_{25}N_4O_4S$: 525.1591; found 525.1595. HPLC Purity: 97.1%.

**$^{1}H$ NMR of JS25 in $d^6$-DMSO:**

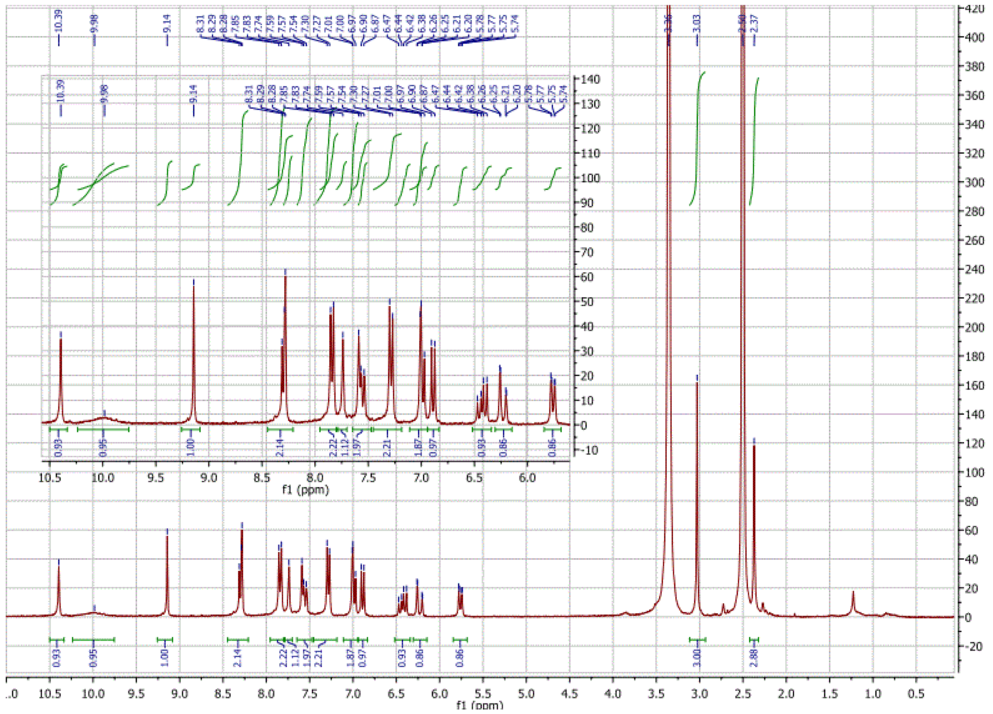

## HPLC trace of JS25:

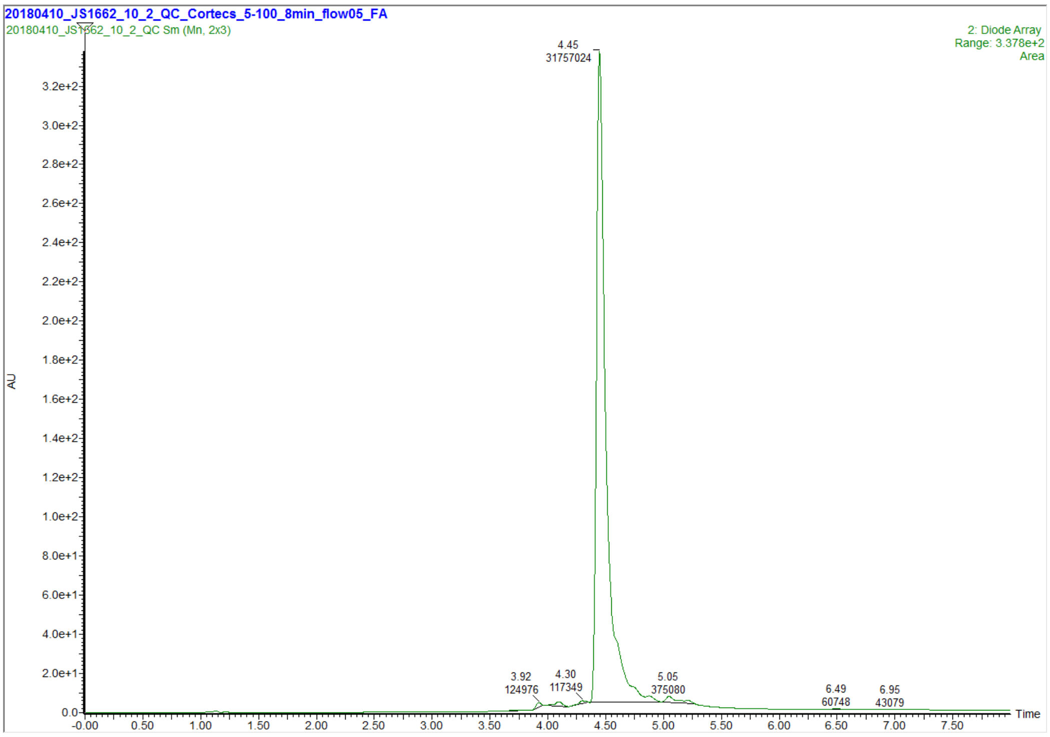


## N-(3-(8-(4-(methylsulfonamido)phenyl)-2-oxobenzo[h][1,6]naphthyridin-1(2H)-yl)phenyl)acrylamide (JS26)

Prepared using the procedure described for **9a**. Compound **JS26** was obtained as a light-yellow solid (20 mg; 18%). $^{1}$H NMR (300 MHz, d$^{6}$-DMSO): δ 10.42 (s, 1H), 9.94 (brs, 1H), 9.15 (s, 1H), 8.32 – 8.28 (m, 2H), 7.89 (d, *J* = 8.2 Hz, 1H), 7.85 – 7.76 (m, 3H), 7.61 (t, *J* = 8.1 Hz, 1H), 7.52 (dd, *J* = 9.3, 2.2 Hz, 1H), 7.29 (d, *J* = 8.7 Hz, 2H), 7.17 (dd, *J* = 7.9, 1.1 Hz, 1H), 6.91 (dd, *J* = 9.4, 8.5 Hz, 2H), 6.43 (dd, *J* = 17.0, 10.0 Hz, 1H), 6.24 (dd, *J* = 17.0, 2.1 Hz, 1H), 5.77 (dd, *J* = 9.9, 2.0 Hz, 1H), 3.03 (s, 3H). HRMS (ESI): *m/z* $[M + H]^{+}$ calc. for $C_{28}H_{23}N_{4}O_{4}S$: 511.1435; found: 511.1439. HPLC Purity: 98.0%.

## $^1$H NMR of JS26 in d$^6$-DMSO:

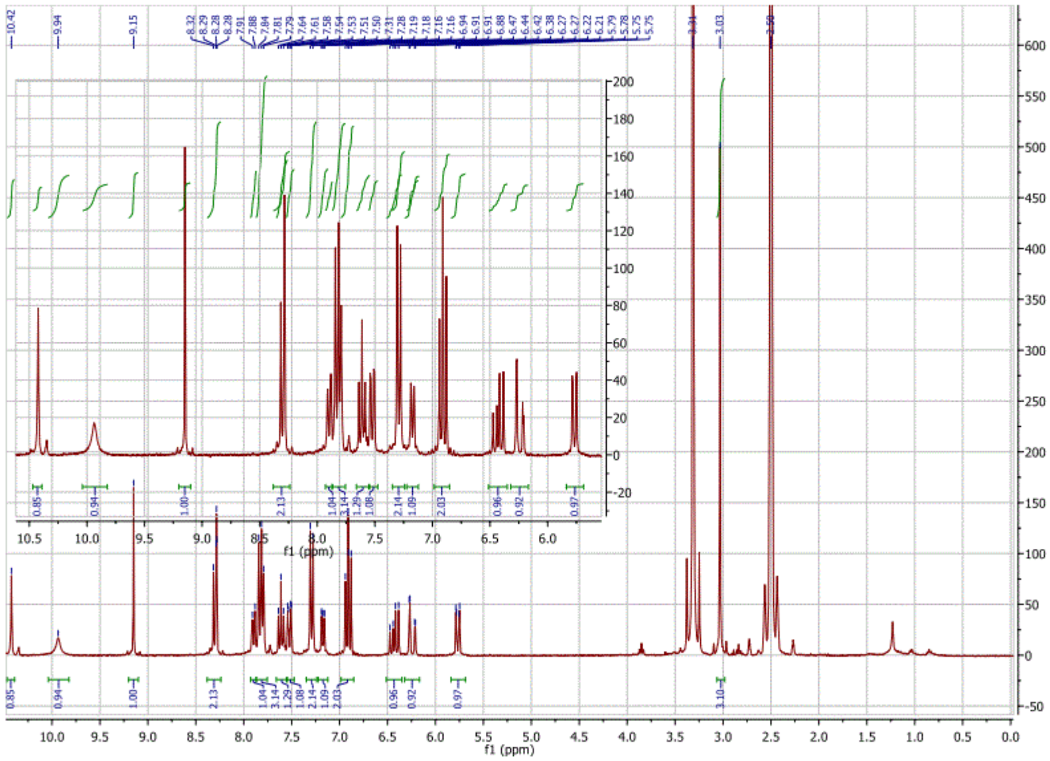


## HPLC trace of JS26:

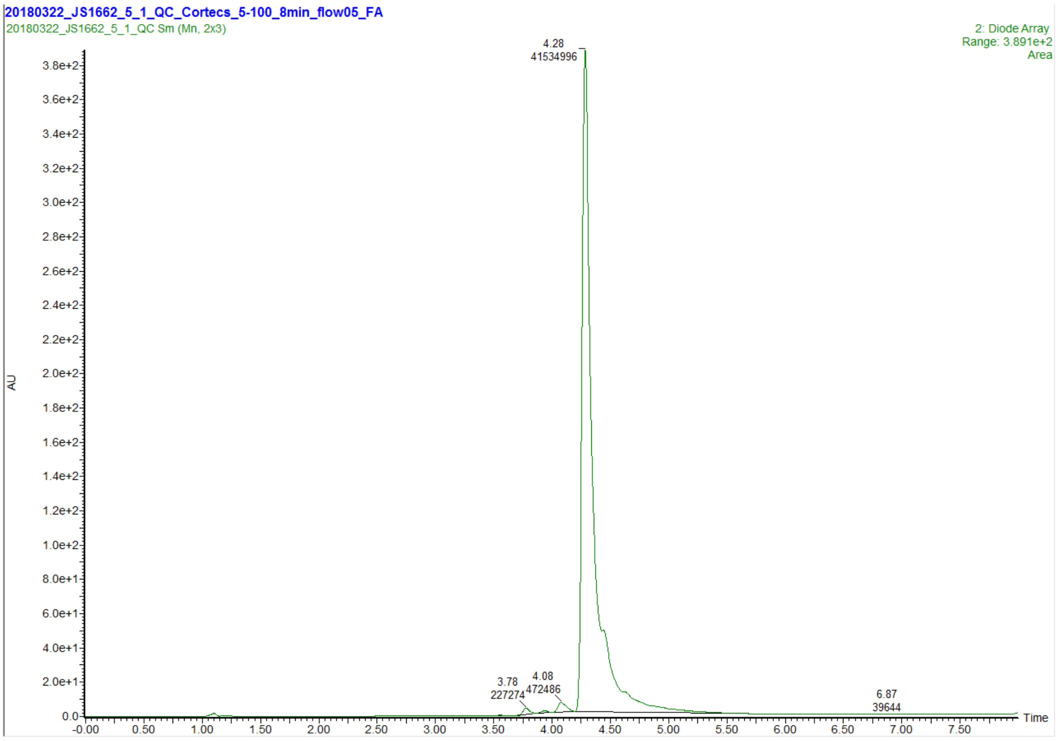

**N-(3-(8-(4-(methylsulfonyl)piperazin-1-yl)-2-oxobenzo[h][1,6]naphthyridin-1(2H)-yl)phenyl)acrylamide (JS27)**

Prepared using the procedure described for **9a**. Compound **JS27** was obtained as a light-yellow solid (10 mg; 6%). $^{1}$H NMR (300 MHz, d$^{6}$-DMSO): δ 10.44 (s, 1H), 8.97 (s, 1H), 8.19 (d, *J* = 9.4 Hz, 1H), 7.86 (m, 1H), 7.74 (t, *J* = 2.0 Hz, 1H), 7.59 (t, *J* = 7.9 Hz, 1H), 7.30 (d, *J* = 2.8 Hz, 1H), 7.12 (ddd, *J* = 7.8, 2.0, 1.0 Hz, 1H), 6.99 (dd, *J* = 9.9, 2.9 Hz, 1H), 6.74 (d, *J* = 9.4 Hz, 1H), 6.64 (d, *J* = 9.8 Hz, 1H), 6.43 (dd, *J* = 16.9, 10.0 Hz, 1H), 6.24 (dd, *J* = 17.0, 2.1 Hz, 1H), 5.78 (dd, *J* = 10.0 Hz, 2.0 Hz, 1H), 3.45 (t, *J* = 5.1 Hz, 4H), 3.19 (t, *J* = 5.1 Hz, 4H), 2.89 (s, 3H). HRMS (ESI): *m/z* $[M + H]^{+}$ calc. for $C_{26}H_{26}N_{5}O_{4}S$: 504.1700; found: 504.1703. HPLC Purity: 96.8%.

**$^{1}$H NMR of JS27 in d$^{6}$-DMSO:**

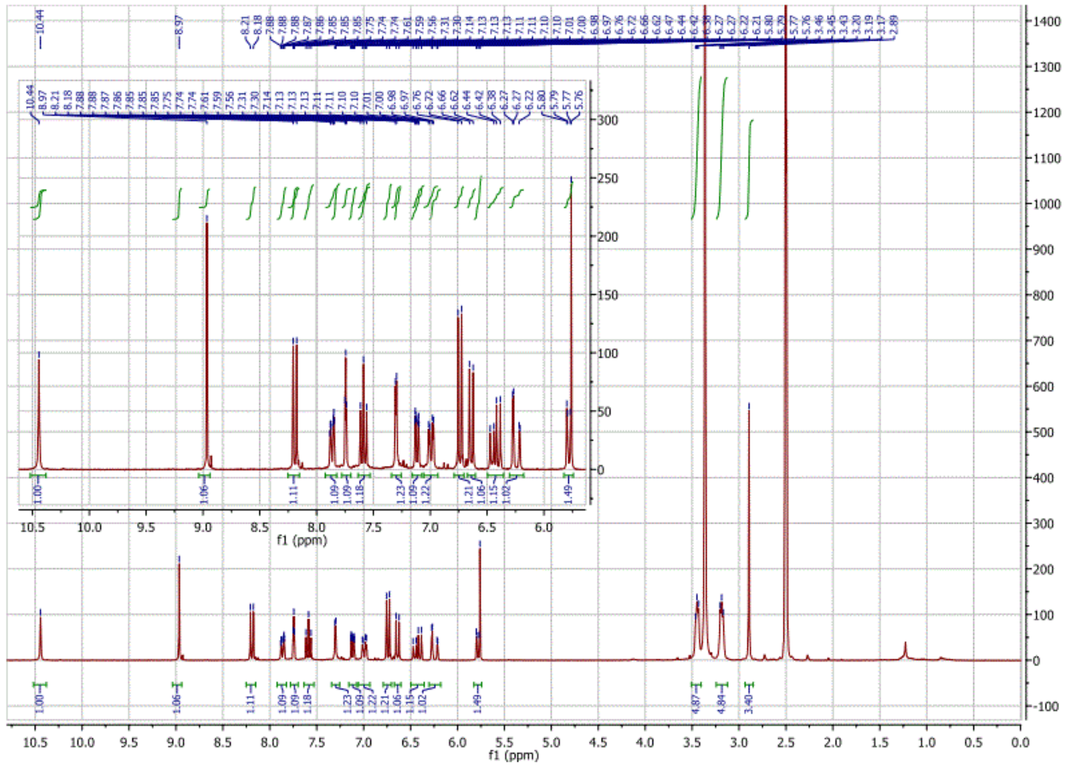

**HPLC trace of JS27:**

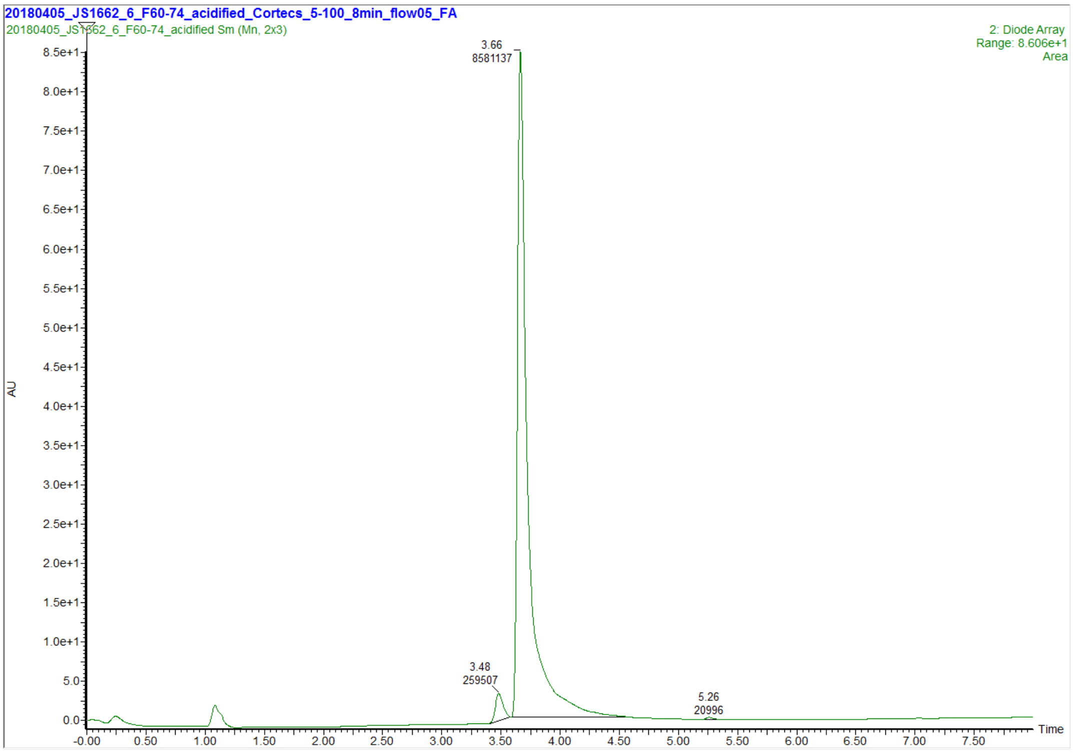


**Ethyl 4-(3-acrylamido-4-methylphenylamino)-6-(4-(methylsulfonamido)phenyl)quinoline-3-carboxylate (28)**

Prepared using the procedure described for **9a**. Compound **28** was obtained as a yellow solid (10 mg; 10%). $^{1}H$ NMR (300 MHz, $CDCl_3$): δ 10.59 (s, 1H), 9.20 (s, 1H), 8.05 – 7.93 (m, 1H), 7.85 - 7.75 (m, 3H), 7.46 (s, 1H), 7.23 – 7.06 (m, 5H), 6.81 (d, *J* = 7.8 Hz, 1H), 6.47 – 6.18 (m, 2H), 5.73 (d, *J* = 9.6 Hz, 1H), 4.44 (q, *J* = 7.1 Hz, 2H), 3.01 (s, 3H), 2.33 (s, 3H), 1.45 (t, *J* = 7.1 Hz, 3H). HRMS (ESI): *m/z* $[M + H]^+$ calc. for $C_{29}H_{29}N_4O_5S$: 545.1853; found 545.1849. HPLC Purity: 95.5%.

**N-(5-(9-bromo-2-oxobenzo[h][1,6]naphthyridin-1(2H)-yl)-2-methylphenyl)acrylamide (29)**

Prepared using the procedure described for **9a**. Compound **29** was obtained as an off-white solid (40 mg; 85%). $^{1}H$ NMR (300 MHz, $d^6$-DMSO): δ 9.68 (s, 1H), 9.15 (s, 1H), 8.29 (d, *J* =

9.5 Hz, 1H), 7.94 (d, $J$ = 8.8 Hz, 1H), 7.79 (dd, $J$ = 8.8, 2.1 Hz, 1H), 7.66 (s, 1H), 7.53 (d, $J$ = 8.0 Hz, 1H), 7.19 (dd, $J$ = 8.0, 2.2 Hz, 1H), 6.94 (d, $J$ = 9.4 Hz, 1H), 6.84 (d, $J$ = 2.1 Hz, 1H), 6.59 (dd, $J$ = 17.0, 10.1 Hz, 1H), 6.20 (dd, $J$ = 17.0, 2.1 Hz, 1H), 5.74 (dd, $J$ = 10.0, 2.1 Hz, 1H), 2.42 (s, 3H). HRMS (ESI): *m/z* $[M + H]^+$ calc. for $C_{22}H_{17}BrN_3O_2$: 434.0499; found: 434.0497. HPLC Purity: 92.2%.

*General Procedure J: Alkylation*

**Methyl 4-(2-methyl-5-(9-(4-(methylsulfonamido)phenyl)-2-oxobenzo[h][1,6]naphthyridin-1(2H)-yl)phenylamino)but-2-enoate (13)**

To a stirred solution of **8a** (73 mg; 0.155 mmol) and $K_2CO_3$ (28 mg; 0.20 mmol; 1.3 equiv.) in DMF (2 mL) at 0 ºC, methyl-4-bromocrotonate (28 mg; 0.16 mmol; 1 equiv.) in DMF (2 ml) was slowly added over 1h, and the mixture stirred at 0ºC. The reaction was allowed to warm to rt overnight and after 20h, TLC analysis (5% MeOH in DCM) showed complete consumption of starting material. The solvent was evaporated and the crude partitioned between EtOAc and saturated $NaHCO_3$ aq. The phases were separated and the aqueous phase further extracted with EtOAc (2x). The combined organics were dried over $MgSO_4$ and taken to dryness. The crude applied in a silica column and eluted with a gradient from 100:0 to 97:3 (DCM:MeOH). The desired fractions were collected and taken to dryness to afford the title compound as a light-yellow solid. (30 mg; 34% yield). $^1$H NMR (300 MHz, d$^6$-DMSO): δ 9.09 (s, 1H), 8.26 (d, J = 9.5 Hz, 1H), 8.08 – 8.00 (m, 2H), 7.48 – 7.39 (m, 4H), 7.33 (d, J = 8.6 Hz, 1H), 7.15 (d, J = 7.6 Hz, 1H), 6.89 (d, J = 9.4 Hz, 1H), 6.79 – 6.72 (m, 1H), 6.66 (d, J = 2.0 Hz, 1H), 6.48 (dd, J = 7.7, 2.0 Hz, 1H), 6.05 (d, J = 15.7 Hz, 1H), 5.38 (m, 0.5H), 5.28 (brs, 2H), 4.57 (d, J = 4.5 Hz, 1H), 4.42 (dt, J = 14.3, 7.3 Hz, 0.5H), 3.65 (s, 3H), 3.10 (s, 3H), 2.21

(s, 3H). HRMS (ESI): *m/z* $[M + H]^+$ calc. for $C_{31}H_{29}N_4O_5S$: 569.1853; found: 569.1853. HPLC Purity: 99.5% (2 isomers with 1:5.3 ratio).

**N-(4-(1-(3-(3-cyanoallylamino)-4-methylphenyl)-2-oxo-1,2-dihydrobenzo[h][1,6]naphthyridin-9-yl)phenyl)methanesulfonamide (12)**

Prepared using the procedure described for **13**. Compound **12** was obtained as a pale-yellow solid (25 mg; 39%). $^1$H NMR (300 MHz, d$^6$-DMSO): δ 9.10 (s, 1H), 8.27 (d, *J* = 9.4 Hz, 1H), 8.08 – 8.03 (m, 2H), 7.47 – 7.35 (m, 4H), 7.33 (d, *J* = 8.5 Hz, 1H), 7.17 (d, *J* = 7.8 Hz, 2H), 6.89 (dd, *J* = 9.4, 2.3 Hz, 2H), 6.67 (m, 1H), 6.49 (dd, *J* = 7.8, 2.3 Hz, 1H), 5.87 (dd, *J* = 31.2, 13.6 Hz, 0.5H), 5.30 (brs, 2H), 4.64 (d, *J* = 6.6 Hz, 0.5H), 4.54 (d, *J* = 5.0 Hz, 0.5H), 4.26 (dt, *J* = 13.5, 6.5 Hz, 0.5H), 3.11 (s, 3H), 2.20 (s, 3H). HRMS (ESI): *m/z* $[M + H]^+$ calc. for $C_{31}H_{25}N_5O_3S$: 536.1751; found: 536.1751. HPLC Purity: 99.0% (2 isomers with 1:1.65 ratio).

## 3. METHODS

### *In silico* cLog and LogS:

cLogP and LogS were calculated using SwissADME software.[3] cLog P is a consensus value obtained as the arithmetic mean of five freely available predictive models (XLOGP3,[4] WLOGP,[5] MLOGP,[6,7] SILICOS-IT[8] and iLOGP[9]) and LogS is the arithmetic mean of two topological methods (ESOL model[1] and Ali *et al.*[2]).

### Dynamic Light Scattering:

Dynamic light scattering (Zetasizer Nano S, Malvern, UK) was used to determine compound colloidal aggregation. The particle sizes were measured at 25 °C. A 10 mM stock solution of test compounds was prepared in DMSO, following dilution to deionized and filtered water to obtain an analyte solution of 10 μM (0.1% DMSO). Colloidal aggregation was measured through sequential dilutions at 10 μM, 1 μM and 0.1 μM.

### Multidimensional scaling:

BTK and BMX ligands, as well as the annotated activities, were collected from ChEMBLv24. Data was pre-processed as previously reported[10] and the CATS2 descriptors were calculated[11] (MOE, chemical computing group implementation). Dimensionality was reduced through multidimensional scaling algorithm.

### Artificial Membrane Permeability (PAMPA):

The PAMPA Evolution™ instrument was used to determine permeability, at Pion Inc. In PAMPA, a sandwich is formed such that each composite well is divided into two chambers, separated by a 125 μm thick microfilter disc (0.45 μm pores), coated with Pion GIT-0 phospholipid mixture. The effective permeability, Pe (x$10^{-6}$ cm/sec), of each compound was measured at pH 6.8 in the donor compartment using low-binding, low UV Prisma buffer. The drug-free acceptor compartment was filled with acceptor sink buffer containing a scavenger at

the start of the test. The proprietary scavenger mimics serum proteins and blood circulation, thus creating sink conditions. Aqueous solutions of studied compounds are prepared by diluting and thoroughly mixing 3 $\mu$L of DMSO stock in 600 $\mu$L of Prisma HT buffer. Final concentration of organic solvent (DMSO) in aqueous buffer is ≤ 0.5% (v/v). The reference solution is identical to the donor at time zero, so that any surface adsorption effects from the plastic ware is compensated. The PAMPA sandwich was assembled and allowed to incubate for ~15 hours. The solutions in the donor compartment were un-stirred within duration of the experiment. Thus, the thickness of the aqueous boundary layer expected to be about 1000 $\mu$m. The sandwich was then separated, and both the donor and receiver compartments were assayed for the amount of drug present by comparison with the UV spectrum obtained from reference standards. Mass balance was used to determine the amount of material remaining in the membrane filter and on the plastic (%R). All values are reported as the average of quadruplicates.

**BMX Biochemical Activity Assay:**

BMX kinase activity ($IC_{50}$) was performed at CEREP-France. Briefly, the inhibition of the human recombinant Bmx kinase is quantified by measuring the phosphorylation of the substrate biotinyl-βAβAβAEEEPQYEEIPIYLELLP using a human recombinant enzyme expressed in insect cells and the HTRF detection method. The test compound, reference compound or water (control) are preincubated for 5 min at room temperature with the enzyme (10 ng) in a buffer containing 50 mM Hepes/NaOH (pH 7.4), 10 mM $MnCl_2$, 3.6 mM DTT, 40 $\mu$M Na3VO4 and 0.005% Tween 20. Thereafter, the reaction is initiated by adding 0.4 $\mu$M of the substrate biotinyl-βAβAβAEEEPQYEEIPIYLELLP and 0.7 $\mu$M ATP, and the mixture is incubated for 60 min at room temperature. For control basal measurements, the enzyme is omitted from the reaction mixture. Following incubation, the reaction is stopped by adding 33 mM EDTA. The fluorescence acceptor (XL665-labeled streptavidine) and the fluorescence

donor (anti-phospho-tyrosine-66K antibody labeled with europium cryptate) are then added. After 60 min, the fluorescence transfer is measured at λex=337 nm, λem=620 nm and λem=665 nm using a microplate reader (Rubystar, BMG). The enzyme activity is determined by dividing the signal measured at 665 nm by that measured at 620 nm (ratio). The results are expressed as a percent inhibition of the control enzyme activity.

**BTK Biochemical Activity Assay:**

BTK kinase activity ($IC_{50}$) was performed at DiscoverX through a radiometric assay with the KinaseProfiler platform. BTK is incubated with 8 mM MOPS pH 7.0, 0.2 mM EDTA, 250 μM KVEKIGEGTYGVVYK (Cdc2 peptide), 10 mM MgAcetate, and [gamma-33P]-ATP (10 μM). The reaction is initiated by the addition of the Mg/ATP mixture. After incubation for 40 min at room temperature, the reaction is stopped by the addition of phosphoric acid to a concentration of 0.5%. 10 μL of the reaction is then spotted onto a P30 filtermat and washed four times for 4 minutes in 0.425% phosphoric acid and one in methanol prior to drying and scintillation counting.

**Differential Scanning Fluorimetry:**

DSF was performed in MicroAmp™ EnduraPlate™ Optical 96-Well Clear Reaction Plates with Barcode (Applied Biosystems, Life Technologies, California, USA) using a QuantStudio 7 Flex Real-Time PCR System (Applied Biosystems). Pre-incubation of the protein with the compound for 2 hours at 4ºC was required prior to DSF experiments. The final reaction mixture (20 μL of total volume) contained 4 μg of $His_6$-BMX, 4-fold of Protein Thermal Shift™ Dye (Applied Biosystems) diluted in protein buffer solution, and 100 μM of compound. The temperature was increased from 25ºC to 90ºC with an increment rate of 0.016ºC/s. Excitation and emissions filters were applied for Protein Thermal Shift™ Dye (470nm and 520nm,

respectively) and for ROX reference dye (580nm and 623nm, respectively). The melting temperatures were obtained by taking the midpoint of each transition.

**Surface Plasmon Resonance:**

The kinetic and affinity parameters of protein-compound interaction were evaluated by SPR. Experiments were carried out in a Biacore 4000 instrument (Biacore AB, GE Healthcare Life Sciences, Uppsala, Sweden) at 25 °C. $His_6$-BMX protein was diluted to 10 $\mu$g/mL in sodium acetate pH 5.5, in the presence of 5 $\mu$M staurosporine and immobilized onto CM5 (Series S) sensor chips, using the standard amine coupling procedure. Prior to immobilization, the carboxymethylated surface of the chip was activated with 400 mM 1-ethyl-3-(3-dimethylaminopropyl)-carbodiimide and 100 mM N-hydroxysuccinimide for 10 min. HBS-N (10 mM HEPES pH7.4, 150mM NaCl) was used as the background buffer. Protein was coupled to the surface after 2 to 10 min injection times, at a flow rate of 10 $\mu$L/min, in order to reach 1500 to 3500 response units (RU). The remaining activated carboxymethylated groups were blocked with a 7 min injection of 1 M ethanolamine pH 8.5. Compounds were pre-diluted in DMSO to 50x the desired highest tested concentration and diluted afterwards in running buffer (20 mM HEPES pH 7.4, 150 mM NaCl, 1 mM DTT, 0.1 mM EGTA, 0.05 % (v/v) TWEEN-20, 5 mM $MgCl_2$) in order to reach 2% of DMSO concentration. A DMSO solvent correction (1% - 3%) was performed to account for variations in bulk signal and to achieve high-quality data. Each compound was injected over immobilized $His_6$-BMX for 220 s (30 $\mu$L $min^{-1}$; association phase) followed by 600 to 2000 s of buffer flow (dissociation phase) at a maximum concentration of 0.5 $\mu$M or 10 $\mu$M for high and low affinity binders, respectively, and diluted five times in 2-fold dilution series. All sensorgrams were processed by first subtracting the binding response recorded from the control surface (reference spot), followed by subtracting the buffer blank injection from the reaction spot. All datasets were fit to a simple 1:1 Langmuir

interaction model with the provided Biacore 4000 Evaluation software, to determine kinetic rate constants ($k_{on}$, $k_{off}$) or steady-state affinity ($K_{Dss}$).

**Kinetic characterization using an ADP-GLO™ kinase assay:**

The BMX enzyme system (Catalog. V4512) and the ADP-Glo™ kinase assay were purchased from Promega Corporation (USA). In each kinase reaction, the concentration of BMX was set to 4.5 ng/$\mu$L. The peptide substrate Poly (4:1 Glu, Tyr) and ATP concentrations were set to 0.25 mg/mL and 50 $\mu$M, respectively. BMX was pre-incubated with different inhibitor concentrations (10-fold serial dilutions, starting at 1 mM), over different time periods (2 - 60 min), before initiating the kinase reactions. Reactions were started by adding 2.5X Poly E4Y1/ATP mixture. The reactions were carried out in a 384-well plate, stopping all at the same time with the addition of 5 $\mu$L of ADP-Glo™ reagent, to consume the remaining ATP within 40 min. Then, 10 $\mu$L of kinase detection reagent was added into the wells and incubated for 30 min to produce a luminescence signal. The signal was measured using an Infinite M200 Microplate Reader (Tecan Group Ltd, Switzerland), with an integration time of 0.250s. The observed rate constants for inhibition ($k_{obs}$), at different inhibitor concentrations, were determined from the slope of a semi-logarithmic plot of inhibition versus time, and re-plotted against inhibitor concentration (nM). The experimental values were fitted into a hyperbolic function using GraphPad Prism 8 (GSL Biotech LLC, USA), to obtain the $K_I$, $k_{inact}$, and $k_{inact}/K_I$, as described previously.[12]

**Native Mass spectrometric analysis:**

For native MS analysis, protein sample was buffer exchanged to 200 mM ammonium acetate, pH7.6 and analyzed on a modified Q-exactive hybrid quadrupole-Orbitrap mass spectrometer (Thermo Fisher Scientific) using gold-coated glass needles. Typical native MS settings are a source fragmentation voltage of 50 V and capillary temperature of 30 °C. Denaturing MS

analysis of drug conjugated protein was performed by liquid chromatography-MS (LCMS) using a Dionex UltiMate 3000 RSLC Nano system coupled with a LTQ Orbitrap XL hybrid ion trap-Orbitrap spectrometer (Thermo Fisher Scientific). The protein sample was directly loaded onto a C18 trap cartridge (Acclaim PepMap100, C18, 1 mm × 5 mm Thermo Scientific), desalted with 100% buffer A (100% $H_2O$ and 0.1% formic acid) at a flow rate of 10 $\mu$l/min for 10 min, eluted and separated onto a C18 column (Acclaim PepMap100, C18, 75 $\mu$m × 15 cm, Thermo Scientific) with a linear gradient from 0% to 100% buffer B (50% isopropanol, 45% acetonitrile, 5% $H_2O$ and 0.1% formic acid) at a flow rate of 300 nl/min in 50 min. Typical MS conditions were a spray voltage of 1.8 kV and capillary temperature of 300 °C. The LTQ-Orbitrap XL was set up in positive ion mode with ion trap scanning (m/z 335-2000). The proteomics analysis of drug conjugated protein was performed on the same LCMS system with minor changes. Tryptic digested peptides were loaded to a C18 trap cartridge, desalted with 100% buffer A (100% $H_2O$ and 0.1% formic acid) at 20 $\mu$l/min for 5 min and separated on a C18 analytical column with a linear gradient from 0% to 60% buffer B (80% acetonitrile, 20% $H_2O$ and 0.1% formic acid) at flow rate of 300 nl/min. LTQ-XL was operated in data-dependent acquisition mode with one full MS scan followed by 5 MS/MS scans with collision-induced dissociation. For full MS scan, the mass range was set to 335 to 2000 *m/z* at a resolution of 60000. For tandem MS scan, the CID normalized energy was 35%.

**Crystallization, data collection, and structure determination of BMX catalytic domain in complex with the inhibitor JS24:**

The guidelines for plasmid construction and vector cloning are described by Muckelbauer *et al.*.[13] The expression of BMX protein using Sf-9 cells, as well as the purification process, were optimized to improve sample quality at the end of purification, in order to increase the likelihood of protein crystallization.[14] The purified BMX tyrosine kinase was concentrated to a final concentration of 10 mg/mL and pre-incubated for 2 h at 20 ºC with a 2-fold concentration

of the inhibitor **JS24**. The trials were carried out using the sitting-drop vapor diffusion method on the Mosquito® LCP crystallization robot (TP Labtech Ltd, Hertfordshire, UK). The drops consisted of 0.150 $\mu$L of the reservoir solution mixed with an equal volume of the protein sample, equilibrated against a 45 $\mu$L reservoir. The crystals appeared after 2 days in a lead condition consisting of 0.2 M imidazole-malate buffer, pH 5.5, with 42% v/v PEG 600. The better-shaped crystals were analyzed at the European Synchrotron Radiation Facility (ESRF) in Grenoble, France. An X-ray diffraction data set to 2.0 Å was collected at ESRF beamline ID30A-3 with a Dectris EIGER X 4M detector from a cryocooled crystal at 100 K. The diffraction data were processed with AutoPROC and XDS,[15] and the data processing and refinement statistics are summarized in Supporting Information (Table S4). Two diffraction datasets were obtained: in the first, a spherical region of reciprocal space to 2.2 Å resolution was defined, and in the second a triaxial ellipsoidal region to a maximal resolution of 1.95 Å was selected with the STARANISO module of AutoPROC.[15] The structure of hBMX in complex with ligand **JS24** was determined by molecular replacement with PHASER[16] as implemented in the CCP4 program suite[17,18] using the PDB entry 3SXS[18] as a search model, without including ligands and water molecules. Two independent copies of the search model were located in the crystal structure, and model rebuilding was carried out with BUCANEER[19] and COOT.[20] Initial structure refinement was undertaken with REFMAC.[21] The stereochemical restraint dictionary for ligand **JS24** was created with JLIGAND[22] and the ligand was manually fitted into the electron density using COOT. Refinement was continued with PHENIX,[23] alternating with manual model editing in COOT between refinements against $\sigma_A$-weighted $2|F_o|-|F_c|$ and $|F_o|-|F_c|$ electron density maps. In the final refinement cycles, hydrogen atoms were added and refined in calculated positions, Translation-Libration-Screw rigid-body anisotropic atomic displacement parameters were refined, water molecules added automatically and the relative weights between the crystallographic and stereochemical energy terms optimized.

Each BMX molecule was divided into 4 rigid-body segments, estimated from the TLSMD server[24] using the isotropic atomic displacement parameters from a previous refinement run. The final refinement was carried out to 2.0 Å against the STARANISO dataset and the statistics are included in ESI (Table S5). Figures were prepared with PYMOL.[25]

**Molecular Dynamics simulations on BMX covalently linked to JS24 and JS27:**

The X-ray structure of **JS24** bound to BMX was used as starting structure in the MD simulations. Parameters for ligands **JS24** and **JS27** were generated with the antechamber module of Amber18,[26] using the general Amber force field (GAFF),[27] with partial charges set to fit the electrostatic potential generated with HF/6-31G(d) by RESP.[28] The charges were calculated according to the Merz-Singh-Kollman scheme using Gaussian 09.[29] The ff14SB force field, which is an evolution of the Stony Brook modification of the Amber 99 force field (ff99SB),[30] was used for the protein. Each complex was immersed in a water box with a 10 Å buffer of TIP3P water molecules.[31] The system was neutralized by adding explicit counter ions. A two-stage geometry optimization approach was performed. The first stage minimizes only the positions of solvent molecules and ions, and the second stage is an unrestrained minimization of all the atoms in the simulation cell. The systems were then gently heated by incrementing the temperature from 0 to 300 K under a constant pressure of 1 atm and periodic boundary conditions. Harmonic restraints of 30 kcal·mol$^{-1}$ were applied to the solute, and the Andersen temperature coupling scheme[32] was used to control and equalize the temperature. The time step was kept at 1 fs during the heating stages, allowing potential inhomogeneities to self-adjust. Water molecules are treated with the SHAKE algorithm such that the angle between the hydrogen atoms is kept fixed. Long-range electrostatic effects are modelled using the particle-mesh-Ewald method.[33] An 8 Å cutoff was applied to van der Waals interactions. Each system was equilibrated for 2 ns with a 2-fs time-step at a constant volume and temperature of

300 K. Production trajectories were then run for additional 0.5 μs under the same simulation conditions.

**Kinase Selectivity:**

Kinome selectivity was performed with DiscoverX KINOMEscan™ technology.

**Target Engagement Intracellular Kinase Assay:**

In-cell target engagement was performed at the Reaction Biology Corporation (USA) using the NanoBRET™ technology. Very briefly, HEK296 cells, purchased from ATCC, were transfected with BMX and treated in duplicate with test compounds, BMX-IN-1 or JS25, and with the reference compound Dasatinib, for 1 hour of incubation. Compounds were diluted 10 times with 3-fold dilution, starting at 1 $\mu$M. Curve fits were performed only when % NanoBret signal at the highest concentration of compounds was less than 55%. The $IC_{50}$ values were determined using the GraphPad Prism 8 (USA).

**Cellular activity Assay:** *LNCaP and PC-3 Cell Growth Assay*

Cells were seeded in white, opaque-bottom 96-well plates at 5000 cells/well (LNCaP) or 2000 cells/well (PC-3) in a total volume of 100 $\mu$L of culture media. Serial diluted compounds (2-fold) in 100 $\mu$L media were added to the cells 24 hours later. After 96 hours incubation cellular viability was assessed by CellTiter-Glo® (Promega) according to the manufacturer's instructions. The values were normalized to vehicle and $IC_{50}$ was calculated using GraphPad Prism 8 (USA).

**Anti-proliferative co-treatment assays in LNCaP:**

LNCaP cells were seeded in 96 well-plates at 5000 cells/well in in a total volume of 100 $\mu$L of culture media and incubated for 24 hours to allow for attachment. After incubation, cells were treated in triplicate, in a combinatorial-fashion with JS24 (2 $\mu$M and 3 $\mu$M), JS25 (5 $\mu$M

and 6 $\mu$M), JS26 (6 $\mu$M), AKT1/2 (1 $\mu$M and 2 $\mu$M), Flutamide (25 $\mu$M and 50 $\mu$M), and PI3K inhibitor (3 $\mu$M and 3.5 $\mu$M).

**Cellular activity Assay:** *Multi cell line Growth Assay*

Compound activity was profiled against 14 human cell lines from different tissues in a 384-well format, opaque white assay plates at 500-1000 cells per well using a semi-automated system. Cells were incubated at 37 ºC and 5% $CO_2$. Compound stocks were plated in a 384-well format in 11-point and 2-fold concentration ranges. Compounds were pin-transferred into duplicate assay plates and incubated for72h. ATP levels were assessed by CellTiter-Glo® (Promega) according to the manufacturer's instructions. The values were normalized to vehicle and $GI_{50}$ was calculated using GraphPad Prism 8. When ambiguous fit was observed curves were top (100%) and bottom (0%) constrained and $GI_{50}$ was determined with 4-P least squares fit. In these cases SD is not calculated by GraphPad Prism 8.

**Propidium iodide assay:**

LNCaP cells were seeded in 24 well-plates at 8000 cells/well in in a total volume of 500 $\mu$L of culture media and incubated for 24 hours to allow for attachment. After this time, 5 $\mu$M of each compound diluted in culture medium was added to the cells. After 64 hours of treatment, cells were harvested after trypsinization (TrypLE Express, LifeTechnologies, USA) into round-bottom FACs tubes, and washed with 10 % FBS in PBS. Cells were then re-suspended in 5 $\mu$g/ml of propidium iodide diluted in wash buffer and analyzed directly after 15 minutes using an LSR Fortessa X-20 flow cytometer equipped with a 488 nm laser and a 670LP and 695/40BP combination of filters (BD Biosciences, USA). Results are shown as percentages of controls (i.e. vehicles) and represent average ± SD (of triplicates).

**BMX degradation assay in PC3 cells: JS25** and **BMX-IN-1** were tested at 10 $\mu$M concentration against PC3 cells, plated on 6-well plates, a day after attachment. DMSO was used as a control vehicle. At 24 h and 72 h of incubation, cells were washed in 1X PBS and lysed in 100 $\mu$L RIPA Buffer (in house) supplemented with EDTA-free Protease Inhibitor Cocktail (Merk) and DNase I (Merk). The lysis procedure was performed by re-suspending cells continuously, followed by a incubation of 30 min on ice. BMX antibody (Abcam) and tubulin antibody (Cell signaling Technology) were used in the Western blot. Band intensity was measured in ImageJ.

**Accession Codes:**

The final refined coordinates and observed structure factors for The PDB access code for the structure of **JS24** bound to BMX, were submitted to the Worldwide Protein Data Bank with accession code 6I99.

## 4. REFERENCES


1. J. S. Delaney, *J. Chem. Inf. Model*, 2004, **44**, 1000–1005.

2. J. Ali, P. Camilleri, M. B. Brown, A. J. Hutt and S. B. Kirton, *J. Chem. Inf. Model.*, 2012, **52**, 420–428.

3. A. Daina, O. Michielin and V. Zoete, *Sci. Rep.*, 2017, **7**, 42717.

4. T. Cheng, Y. Zhao, X. Li, F. Lin, Y. Xu, X. Zhang, Y. Li, R. Wang and L. Lai, *J. Chem. Inf. Model.*, 2007, **47**, 2140–2148.

5. S. A. Wildman and G. M. Crippen, *J. Chem. Inf. Model.*, 1999, **39**, 868–873.

6. I. Moriguchi, S. Hirono, Q. Liu, I. Nakagome and Y. Matsushita, *Chem. Pharm. Bull.*, 1992, **40**, 127–130.

7. I. Moriguchi, S. Hirono, I. Nakagome and H. Hirano, *Chem. Pharm. Bull.*, 1994, **42**, 976–978.

8. http://silicos-it.be.s3-website-eu-west-1.amazonaws.com/software/filter-it/1.0.2/filter-it.html.

9. A. Daina, O. Michielin and V. Zoete, *J. Chem. Inf. Model.*, 2014, **54**, 3284–3301.

10. T. Rodrigues, M. Werner, J. Roth, E. H. G. da Cruz, M. C. Marques, P. Akkapeddi, S. A. Lobo, A. Koeberle, F. Corzana, E. N. da Silva Júnior, O. Werz and G. J. L. Bernardes, *Chem. Sci.*, 2018, **9**, 6899–6903.

11. M. Reutlinger, C. P. Koch, D. Reker, N. Todoroff, P. Schneider, T. Rodrigues and G. Schneider, *Mol. Inform.*, 2013, **32**, 133–138.

12. B. -F. Krippendorff, R. Neuhaus, P. Lienau, A. Reichel and W. Huisinga, *J. Biomol. Screen.*, 2009, **14**, 913–923.

12. J. Muckelbauer, J. S. Sack, N. Ahmed, J. Burke, C. Y. Chang, M. Gao, J. Tino, D. Xie and A. J. Tebben, *Chem. Biol. Drug Des.*, 2011, **78**, 739–748.

13. U. T. Rüegg and B. Gillian, *Trends Pharmacol. Sci.*, 1989, **10**, 218–220.

14. B. B. Sousa, M. F. Q. Sousa, M. C. Marques, J. D. Seixas, J. A. Brito, P. M. Matias, G. J. L. Bernardes and A. Roldão, *Bioprocess Biosyst. Eng.*, 2020, DOI: 10.1007/s00449-020-02421-6.

15. C. Vonrhein, C. Flensburg, P. Keller, A. Sharff, O. Smart, W. Paciorek, T. Womack and G. Bricogne, *Acta Crystallogr. D Biol. Crystallogr.*, 2011, **67**, 293–302.

16. A. J. McCoy, R. W. Grosse-Kunstleve, P. D. Adams, M. D. Winn, L. C. Storoni and R. J. Read, *J. Appl. Crystallogr.*, 2007, **40**, 658674.

17. L. Potterton, S. McNicholas, E. Krissinel, J. Gruber, K. Cowtan, P. Emsley, G. N. Murshudov, S. Cohen, A. Perrakis and M. Noble, *Acta Crystallogr. D Biol. Crystallogr.*, 2004, **60**, 2288–2294.

18. E. Potterton, P. Briggs, M. Turkenburg and E. Dodson, *Acta Crystallogr. D Biol. Crystallogr.*, 2003, **59**, 1131–1137.

19. K. Cowtan, Acta Crystallogr. D Biol. Crystallogr., 2006, **62**, 1002–1011.

20. P. Emsley and K. Cowtan, *Acta Crystallogr. D Biol. Crystallogr.*, 2004, **60**, 2126–2132.

21. G. N. Murshudov, A. A. Vagin and E. J. Dodson, *Acta Crystallogr. D Biol. Crystallogr.*, 1997, **53**, 240–255.

22. A. A. Lebedev, P. Young, M. N. Isupov, O. V. Moroz, A. A. Vagin and G. N. Murshudov, *Acta Crystallogr. D Biol. Crystallogr.*, 2012, **68**, 431–440.

23. P. D. Adams, P. V. Afonine, G. Bunkoczi, V. B. Chen, I. W. Davis, N. Echols, J. J. Headd, L. W. Hung, G. J. Kapral, R. W. Grosse-Kunstleve, A. J. McCoy, N. W. Moriarty, R.

Oeffner, R. J. Read, D. C. Richardson, J. S. Richardson, T. C. Terwilliger and P. H. Zwart, *Acta Crystallogr. D Biol. Crystallogr.*, 2010, **66**, 213–221.

24. J. Painter and E. A. Merritt, *J. Appl. Crystallogr.*, 2006, **39**, 109–111.

25. W. L. DeLano, *CCP4 Newsletter Prot. Crystallogr.*, 2002, **40**, 82-92.

26. D.A. Case, S.R. Brozell, D.S. Cerutti, Cheatham, T.E. III, Cruzeiro, V.W.D.; Darden, T.A.; Duke, R.E.; Ghoreishi, D.; Gohlke, H.; Goetz, A.W.; Greene, D.; Harris, R.; Homeyer, N.; Izadi, S.; Kovalenko, A.; Lee, T.S.; LeGrand, S.; Li, P.; Lin, C.; Liu, J.; Luchko, T.; Luo, R. ; Mermelstein, D.J.; Merz, K.M.; Miao, Y.; Monard, G.; Nguyen, H.; Omelyan, I.; Onufriev, A.; Pan, F.; Qi, R.; Roe, D.R.; Roitberg, A.; Sagui, C.; Schott-Verdugo, S.; Shen, J.; Simmerling, C.L.; Smith, J.; Swails, J.; Walker, R.C.; Wang, J.; Wei, H.; Wolf, R.M.; Wu, X.; Xiao, L.; York D.M.; Kollman P.A. (2018), AMBER 2018, University of California, San Francisco.

27. J. Wang, R. M. Wolf, J. W. Caldwell, P. A. Kollman and D. A. Case, *J. Comput. Chem.*, 2004, **25**, 1157–1174.

28. C. I. Bayly, P. Cieplak, W. Cornell and P. A. Kollman, *J. Phys. Chem.*, 1993, **97**, 10269–10280.

29. Gaussian 09, Revision A.02, M. J. Frisch, G. W. Trucks, H. B. Schlegel, G. E. Scuseria, M. A. Robb, J. R. Cheeseman, G. Scalmani, V. Barone, G. A. Petersson, H. Nakatsuji, X. Li, M. Caricato, A. Marenich, J. Bloino, B. G. Janesko, R. Gomperts, B. Mennucci, H. P. Hratchian, J. V. Ortiz, A. F. Izmaylov, J. L. Sonnenberg, D. Williams-Young, F. Ding, F. Lipparini, F. Egidi, J. Goings, B. Peng, A. Petrone, T. Henderson, D. Ranasinghe, V. G. Zakrzewski, J. Gao, N. Rega, G. Zheng, W. Liang, M. Hada, M. Ehara, K. Toyota, R. Fukuda, J. Hasegawa, M. Ishida, T. Nakajima, Y. Honda, O. Kitao, H. Nakai, T. Vreven, K. Throssell, J. A. Montgomery, Jr., J. E. Peralta, F. Ogliaro, M. Bearpark, J. J. Heyd, E. Brothers, K. N. Kudin, V. N. Staroverov, T. Keith, R. Kobayashi, J. Normand, K. Raghavachari, A. Rendell, J. C. Burant, S. S. Iyengar, J. Tomasi, M. Cossi, J. M. Millam, M. Klene, C. Adamo, R. Cammi, J. W. Ochterski, R. L. Martin, K. Morokuma, O. Farkas, J. B. Foresman, and D. J. Fox, Gaussian, Inc., Wallingford CT, 2016.

30. V. Hornak, R. Abel, A. Okur, B. Strockbine, A. Roitberg and C. Simmerling, *Proteins*, 2006, **65**, 712–725.

31. W. L. Jorgensen, J. Chandrasekhar, J. D. Madura, R. W. Impey and M. L. Klein, *J. Chem. Phys.* , 1983, **79**, 926–935.

32. T. A. Andrea, W. C. Swope and H. C. Andersen, *J. Chem. Phys.*, 1983, **79**, 4576–4584.

33. T. Darden, D. York and L. Pedersen, *J. Chem. Phys.*, 1993, **98**, 10089–10092.
